# Manipulating the type VI secretion system spike to shuttle passenger proteins

**DOI:** 10.1371/journal.pone.0228941

**Published:** 2020-02-26

**Authors:** Sarah Wettstadt, Alain Filloux

**Affiliations:** MRC Centre for Molecular Bacteriology and Infection, Department of Life Sciences, Imperial College London, London, United Kingdom; Universita degli Studi Roma Tre, ITALY

## Abstract

The type VI secretion system (T6SS) is a contractile injection apparatus that translocates a spike loaded with various effectors directly into eukaryotic or prokaryotic target cells. *Pseudomonas aeruginosa* can load either one of its three T6SSs with a variety of toxic bullets using different but specific modes. The T6SS spike, which punctures the bacterial cell envelope allowing effector transport, consists of a torch-like VgrG trimer on which sits a PAAR protein sharpening the VgrG tip. VgrG itself sits on the Hcp tube and all elements, packed into a T6SS sheath, are propelled out of the cell and into target cells. On occasion, effectors are covalent extensions of VgrG, PAAR or Hcp proteins, which are then coined “evolved” components as opposed to canonical. Here, we show how various passenger domains could be fused to the C terminus of a canonical VgrG, VgrG1a from *P*. *aeruginosa*, and be sent into the bacterial culture supernatant. There is no restriction on the passenger type, although the efficacy may vary greatly, since we used either an unrelated T6SS protein, β-lactamase, a covalent extension of an “evolved” VgrG, VgrG2b, or a Hcp-dependent T6SS toxin, Tse2. Our data further highlights an exceptional modularity/flexibility for loading the T6SS nano-weapon. Refining the parameters to optimize delivery of passenger proteins of interest would have attractive medical and industrial applications. This may for example involve engineering the T6SS as a delivery system to shuttle toxins into either bacterial pathogens or tumour cells which would be an original approach in the fight against antimicrobial resistant bacteria or cancer.

## Introduction

The type VI secretion system (T6SS) is a versatile nanoweapon, which bacteria like *Pseudomonas aeruginosa* use to inject effector proteins into target cells. It is proposed that T6SS activity enables this opportunistic human pathogen to establish a niche in any given polymicrobial environment or to modulate host cell responses. The expression and activity of the *P*. *aeruginosa* T6SS is tightly regulated *in vivo* and is notably negatively controlled by the RetS sensor and the RsmA post-transcriptional regulator. As such a *retS* or *rsmA* mutant would display high level of T6SS activity in laboratory grown conditions [1, 2]. The T6SS consists of a membrane complex anchored to the bacterial cytoplasmic membrane, a cytosolic membrane-bound baseplate and a contractile sheath growing into the cytosol [3–7]. Inside the sheath, which is composed of TssB and TssC proteins [8, 9], a tube formed of Hcp hexamers assembles [10]. On top of the Hcp tube and within the baseplate sits the T6SS spike consisting of a trimer of VgrG proteins and a conically shaped PAAR protein [11]. Upon sheath contraction, the Hcp tube together with the VgrG-PAAR spike are propelled out of the cell into the surrounding milieu or into the target cell [12]. The VgrG trimer forms a rigid structure due to the intertwining of the C-terminal hydrophobic β-sheets [13] with each last β-sheet binding to the hydrophobic surface of the PAAR protein [11]. This structural feature gives the T6SS spike a central function in the T6SS: The PAAR protein recruits the cognate VgrG to the baseplate [14] and the needle-like structure of the VgrG-PAAR spike facilitates the puncturing of target membrane thus allowing T6SS-related effectors to enter the target cell [11].

Dependent on their delivery mode, T6SS-delivered effector proteins are classified into two groups: specialised effectors or cargo effectors [15]. A specialised effector contains an N-terminal domain that is a structural component, like VgrG, PAAR or Hcp, that fulfils its role in the assembly of the T6SS machinery [16]. The C-terminal domain, however, is an extension consisting of an effector domain [17, 18]. As such, a specialized effector with a VgrG support is called an “evolved” VgrG. In contrast, a cargo effector interacts with a structural component non-covalently [19].

The effector delivery concept of cargo effectors interacting with Hcp, VgrG or PAAR, was coined “*á la carte*” and reflects on the idea that a given T6SS component, in particular a VgrG, specifically recruits and drives its cognate effector into the target cells [20]. For example, in *P*. *aeruginosa* the three VgrGs, VgrG1a, VgrG1b and VgrG1c were all shown to specifically deliver their cognate effectors Tse6, Tse7 and Tse5, respectively [20–22]. Furthermore, in *Agrobacterium tumefaciens*, *P*. *aeruginosa* and enteroaggregative *Escherichia coli* the cargo effectors Tde1, PldA/PldB and Tle1, respectively, were shown to specifically interact with the C-terminal parts of their cognate VgrG proteins leading to their deliveries [19, 23, 24]. Additionally, some T6SS effectors were identified that require additional proteins, so-called adaptors, from the DUF1795, DUF2169 or DUF4123 family for their deliveries [21, 23, 25–27]. Tde1 and Tse6 are such effectors whose deliveries depend on the presence of a cognate Tap1 (DUF4123) or EagT6 (DUF1795) protein, respectively [21, 23, 28]. Furthermore, T6SS effectors were shown to travel within the Hcp ring of the Hcp tube, which was shown for the effectors Tse1, Tse2 and Tse3 from *P*. *aeruginosa* [23, 29].

While canonical VgrGs are solely composed of the gp5- and gp27-like domains, named after the bacteriophage puncturing device [13], and canonical PAARs composed of the PAAR fold, “evolved” VgrGs and PAARs additionally contain C-terminal extension domains [11, 16]. Two studies reported that the extension domain of a VgrG would be dispensable for VgrG delivery by substituting the extension with a heterologous effector domain [30, 31]. Furthermore, a recent study showed that the cargo effector PldA is delivered by its cognate VgrG4b when it is covalently fused to this canonical VgrG protein [24]. These findings led to the general assumption that by delivering the VgrG spike into target cells, the covalently fused extension domain would be co-transported [32]. However, it is not understood yet, whether a VgrG protein can deliver any extension domain into target cells and whether there are any restrictions to this type of delivery mechanism.

Extension domains of evolved VgrG proteins usually harbour enzymatic activities, which in the cases of *P*. *aeruginosa*, *Vibrio cholerae*, *Burkholderia pseudomallei* or *Aeromonas hydrophila* are involved in host cell subversion to facilitate bacterial uptake [31, 33–35]. So far, two evolved VgrG with antibacterial extension domains have been characterised, namely VgrG-3 from *V*. *cholerae* [30, 36] and VgrG2b from *P*. *aeruginosa* [18]. The C-terminal extension domain of VgrG-3 was shown to degrade peptidoglycan which is thought to confer a competitive advantage against neighbouring bacteria [36], while the zinc-metallopeptidase domain of VgrG2b was proposed to interfere with enzymes involved in peptidoglycan remodelling, *e*.*g*. lytic transglycosylase and penicillin-binding proteins [18]. To avoid self-toxicity mediated by anti-bacterial toxins as well as toxicity of injected effectors from sibling cells, the bacteria need to produce immunity proteins. Thus, *V*. *cholerae* produces a cognate immunity protein TsaB which tightly interacts with VgrG-3 [36] while *P*. *aeruginosa* uses PA0261 as an immunity that directly interacts with VgrG2b [18].

In the present study, we have tried to broaden the spectrum of VgrG chimera that can be transported by the T6SS, and tested whether it would be generally possible to manipulate this system to deliver chosen effector domain. We thus modified the VgrG spike to assess how flexible the delivery mechanisms are. We selected T6SS effectors not naturally using VgrGs for delivery, T6SS effectors from a different bacterial species, or T6SS-unrelated domains/proteins. We show that in some cases canonical VgrGs can be modified into “evolved” forms to deliver different effector domains but it seems clear that this strategy has limitations. Overall, our work highlights a certain level of modularity of the T6SS spike and provides examples on how the system can be manipulated to drive secretion of grafted domains from different origins.

## Materials and methods

### Bacterial strains and growth conditions

Bacterial strains used in this study are described in Table 1. *P*. *aeruginosa* strains were grown in tryptone soy broth (TSB) or LB supplemented with antibiotics where appropriate (spectinomycin 2000 μg mL^-1^) at 37°C with agitation. *E*. *coli* strains were grown in LB broth supplemented with antibiotics where appropriate (streptomycin 50 μg mL^-1^, kanamycin 50 μg mL^-1^).

**Table 1:** Bacterial strains used in this study.

Strain	Characteristics	Source
**P. aeruginosa**		
PAKΔretS	Wild type *P*.* aeruginosa* PAK strain with a deletion in *retS* (PA4856)	Lab collection
PAKΔretSΔvgrG1b	PAKΔr*etS* with a deletion in *vgrG1b* (*PA0095*)	[37]
PAKΔretSΔvgrG1b Δtsei2	PAKΔr*etS* with deletions in *vgrG1b* (*PA0095*) and *tse2- tsi2* (*PA2702-PA2703*)	This study
PAKΔretSΔvgrG1b Δtsei2::lacZ	PAKΔr*etS* with deletions in *vgrG1b* (*PA0095*) and *tse2-tsi2 (PA2702-PA2703*), chromosomal insertion of *lacZ* at *att* site	This study
PAKΔretSΔvgrG1b Δtsei2ΔtssB1	PAKΔr*etS* with deletions in *vgrG1b* (*PA0095*), *tssB1* (*PA0083*), *tse2-tsi2 (PA2702-PA2703*)	This study
PAKΔretSΔvgrG1b Δtsei2::vgrG1a-tse2tsi2	PAKΔr*etS* with deletions in *vgrG1b* (*PA0095*) and *tse2-tsi2 (PA2702-PA2703)*, insertion of gene portion for *tse2* and *tsi2* before *vgrG1a* (*PA0091*) STOP codon	This study
PAKΔretSΔvgrG1b Δtsei2ΔtssB1::vgrG1a-tse2tsi2	PAKΔr*etS* with deletions in *vgrG1b* (*PA0095*), *tssB1* (*PA0083*), *tse2-tsi2 (PA2702-PA2703)*, insertion of gene portion for *tse2* and *tsi2* before *vgrG1a* (*PA0091*) STOP codon	This study
PAKΔretSΔvgrG1b Δtsei2::hcp1^S31Q^	PAKΔr*etS* with deletions in *vgrG1b* (*PA0095*) and *tse2-tsi2 (PA2702-PA2703*), point mutation (S31Q) in *hcp1* (*PA0085*)	This study
PAKΔretSΔvgrG1b Δtsei2::vgrG1a-tse2tsi2::hcp1^S31Q^	PAKΔr*etS* with deletions in *vgrG1b* (*PA0095*) and *tse2-tsi2 (PA2702-PA2703*), insertion of gene portion for *tse2* and *tsi2* before *vgrG1a* (*PA0091*) STOP codon, point mutation (S31Q) in *hcp1* (*PA0085*)	This study
PAKΔretSΔvgrG1b Δtsei2ΔtssB1::vgrG1a-tse2tsi2::hcp1^S31Q^	PAKΔr*etS* with deletions in *vgrG1b* (*PA0095*), *tssB1* (*PA0083*), *tse2-tsi2 (PA2702-PA2703*), insertion of gene portion for *tse2* and *tsi2* before *vgrG1a* (*PA0091*) STOP codon, point mutation (S31Q) in *hcp1* (*PA0085*)	This study
PAKΔretS::vgrG1a-bla_TEM-1_	PAKΔr*etS* with an insertion of *bla*_*TEM-1*_ before *vgrG1a (PA0091)* STOP codon	This study
PAKΔretS::vgrG1a-vgrG2b-CT	PAKΔr*etS* with an insertion of gene 821 bp of *vgrG2b*-*CT* before *vgrG1a (PA0091)* STOP codon	This study
PAKΔretSΔtssB1	PAKΔr*etS* with deletion in *tssB1* (*PA0083*)	[38]
PAKΔretSΔtssB1::vgrG1a- bla_TEM-1_	PAKΔr*etS* with deletion in *tssB1* (*PA0083*) and an insertion of *bla*_*TEM-1*_ before *vgrG1a (PA0091)* STOP codon	This study
PAKΔretSΔtssB1::vgrG1a-vgrG2b-CT	PAKΔr*etS* with deletions in *tssB1* (*PA0083*) and an insertion of gene 821 bp of *vgrG2b*-*CT* before *vgrG1a (PA0091)* STOP codon	This study
PAO1ΔrsmA::pldA-bla_TEM-1_	PAO1 with deletion in *rsmA* (*PA0905*) and an insertion of *bla*_*TEM-1*_ before *pldA (PA3487)* STOP codon	[1]
PAO1ΔrsmAΔtssB2	PAO1 with deletion in *rsmA* (*PA0905*) and *tssB2* (*PA1657*)	This study
PAO1ΔrsmAΔtssB2::vgrG4b-bla_TEM-1_	PAO1 with deletion in *rsmA* (*PA0905*), *tssB2* (*PA1657*) and an insertion of *bla*_*TEM-1*_ before *vgrG4b (PA3486)* STOP codon	This study
PAO1ΔrsmA::vgrG4b-bla_TEM-1_	PAO1 with deletion in *rsmA* (*PA0905*) and an insertion of *bla*_*TEM-1*_ before *vgrG4b (PA3486)* STOP codon	This study
**E. coli**		
DH5α	F–*endA1 glnV44 thi-1 recA1 relA1 gyrA96 deoR nupG purB20 φ80dlacZΔM15* Δ(*lacZYA-argF)U169*, *hsdR17*(rK–mK+), λ–	ThermoFisher
Sm10λpir	Host strain for Mini-CTX1 replication: *thi thr leu tonA lacY supE recA*::*RP4-2-Tc*::*Mu* (Km^R^) λpir	[39]
CC118λpir	Host strain for pKNG101 replication: Δ(*ara-leu) araD ΔlacX74 galE galK-phoA20 thi-1 rpsE rpoB argE (Sm*^*R*^*) recA1 Rfr λpir*	[40]
1047	Helper strain for conjugation: (Km^R^), *oriColE1 RK2- Mob+ RK2-Tra+*	[41]

### DNA manipulation

DNA purification was performed using PureLink Genomic DNA minikit (Life Technologies). Isolation of plasmid DNA was carried out using the QIAprep spin miniprep kit (Qiagen). Restriction endonucleases were used according to the manufacturer’s specifications (New England Biolabs or Roche). Oligonucleotides used are listed in Table 2 and were purchased from Sigma, United Kingdom. The genes or DNA fragments used for the construction of mutator plasmids and deletion mutants were amplified with KOD Hot Start DNA Polymerase (Novagen) as described by the manufacturer with the inclusion of 0.5 M betaine (Sigma). Colony PCR was performed with Taq polymerase (New England Biolabs). DNA sequencing was performed by GATC Biotech.

**Table 2:** Mutagenesis primers used in this study.

Construct	Primer name	Code	Sequence
pKNG101::hcp1^S31Q^	*hcp1* S31Q F	OAL3072	GCTGGCATGGCAATGGGGCATGTCCC
	*hcp1* S31Q R	OAL3073	GGGACATGCCCCATTGCCATGCCAGCACG
	*hcp1* exF	OAL3076	CAACATCAACCGCTCCTTCAAG
	*hcp1* exR	OAL3077	GCGGTGGAGTAGGTCTGTAC
pKNG101::vgrG1a-shuttles	*vgrG1a*_exF	OAL3735	CGAGGAGATCTGGACCGAC
	*vgrG1a*_exR	OAL3736	AAGGACATCGATCCTGCCG
pKNG101::vgrG1a-bla_TEM-1_	*vgrG1a-bla*_*TEM-1*__upF	OAL1347	GCTAGCCACCCAGAAACGCTGGTG
	*vgrG1a-bla*_*TEM-1*__dnR	OAL1348	GCTAGCTTACCAATGCTTAATCAGTGAG
pKNG101::vgrG1a-tse2tsi2	*vgrG1a-tse2tsi2*_upF	OAL1351	GCTAGCATGTCCTACGACTACGAGAA
	*vgrG1a-tse2tsi2*_dnR	OAL1352	GCTAGCCCGAAGACCATCTGTCGTTT
pKNG101::vgrG1a-vgrG2b-CT	*vgrG1a-vgrG2b-CT_*upF	OAL2731	AAGGGCACGCAGCGGCGGTT
	*vgrG1a-vgrG2b-CT_*upR	OAL2732	AACCGCCGCTGCGTGCCCTTCGCCG
	*vgrG1a-vgrG2b-CT_*dnF	OAL2733	GGGATACTGAGGCGGCGGCAT
	*vgrG1a-vgrG2b-CT_*dnR	OAL2734	ATGCCGCCGCCTCAGTATCCCGT
pKNG101::vgrG4b-bla_TEM-1_	*vgrG4b-bla*_*TEM-1*__upF	OAL2603	CTGGCCAAGGACGCGGCCAGCCTG
	*vgrG4b-bla*_*TEM-1*__upR	OAL2604	CAGGCTGGCCGCGTCCTTGGCCAG
	*vgrG4b-bla*_*TEM-1*__dnF	OAL2608	GCATTGGTAATCCATGTTGCAGAA
	*vgrG4b-bla*_*TEM-1*__dnR	OAL2607	CAACATGGATTACCAATGCTTAATCAG

### Construction of *P*. *aeruginosa* mutants

*P*. *aeruginosa* deletion mutants were constructed as described previously [42, 43] using the suicide plasmid pKNG101 [40, 44]. Briefly, to create PAO1Δ*gene-of-interest* (*GOI*), 500-bp DNA fragments of the 5’ (up) and 3’ (dn) ends of the target gene were obtained by PCR using PAO1 chromosomal DNA as a template with two pairs of oligonucleotides (upF/upR and dnF/dnR) (Table 2). To create chimeric genes (S1 Fig), splicing by overlap extension PCRs was performed and initiated by three single PCR fragments. Gene fragments containing approximately 500 bp upstream and downstream of the splice junction were amplified using the overlapping primers upR and dnF as well as an upstream (exF) and downstream (exR) primer from the *P*. *aeruginosa* genome. A third gene fragment containing the chimera of interest was generated using primers upF and dnR that are overlapping with upR and dnF, respectively. Subsequently, two overlap extension PCR steps were undertaken, employing an equimolar ratio of the upstream and downstream fragments as the DNA template. The gene fragments were cloned into pCR-BluntII-TOPO (Invitrogen), their sequences confirmed and sub-cloned into pKNG101 suicide vector (Table 3). The pKNG-derivatives were maintained in *E*. *coli* strain CC118λpir and mobilized into *P*. *aeruginosa* PAK using *E*. *coli* 1047 carrying the conjugative plasmid pRK2013 [41]. Clones, in which double recombination events occurred, resulting in the deletion of *GOI* or fusion to *GOI*, were isolated using counterselection on sucrose plates as previously described [42]. Gene deletion or fusion was verified by PCR using external primers and western blot analysis where appropriate.

**Table 3:** Plasmids used in this study.

Plasmid	Characteristics	Source
pRK2013	Self-transmissible helper plasmid for three-partner conjugations, KmR	[41]
pKNG101	Non-replicative suicide vector to alter *P*.* aeruginosa* chromosome. *ori6K*, *mobRK2*, *sacB*, Sm^R^	Lab collection
pKNG100::hcp1^S31Q^	pKNG101 suicide plasmid to point mutate serine31 to glutamine of *hcp1*, Sm^R^	This study
pKNG101::pldA-bla_TEM-1_	pKNG101 suicide plasmid to integrate *bla*_*TEM-1*_ to *pldA* gene, Sm^R^	[1]
pKNG101::vgrG1a-bla_TEM-1_	pKNG101 suicide plasmid to integrate *bla*_*TEM*-1_ to *vgrG1a* gene, Sm^R^	This study
pKNG101::vgrG1a-tse2tsi2	pKNG101 suicide plasmid to integrate *tse2-tsi2* to *vgrG1a* gene, Sm^R^	This study
pKNG101::vgrG1a-vgrG2b-CT	pKNG101 suicide plasmid to integrate *vgrG2b-CT* to *vgrG1a* gene, Sm^R^	This study
pKNG101::vgrG4b-bla_TEM-1_	pKNG101 suicide plasmid to integrate *bla*_*TEM*-1_ to *vgrG4b* gene, Sm^R^	This study
pKNG101 ΔrsmA	pKNG101 suicide plasmid to delete *rsmA*, a master regulator, Sm^R^	[1]
pKNG101 Δtse2tsi2	pKNG101 suicide plasmid to *tse2-tsi2*, an effector-immunity module, Sm^R^	[20]
pKNG101 ΔtssB1	pKNG101 suicide plasmid to delete *tssB1*, an essential sheath component of the H1-T6SS, Sm^R^	[38]
pKNG101 ΔtssB2	pKNG101 suicide plasmid to delete *tssB2*, an essential sheath component of the H2-T6SS, Sm^R^	[1]
pKNG101 ΔvgrG1b	pKNG101 suicide plasmid to delete *vgrG1b*, encoding one of the H1-T6SS-associated VgrGs, Sm^R^	[37]
pCR®-Blunt II-TOPO®	Subcloning vector for blunt-ended inserts, Km^R^	ThermoFisher
miniCTX::lacZ	Mini-CTX1 harbouring the *lacZ* with a constitutive promoter, Tc^R^	[45]

### Protein secretion assay

Secretion assays were performed similarly as previously described [37]. Bacterial suspension was diluted from overnight cultures in TSB to OD_600_ of 0.1 and grown at 37°C to an OD_600_ of 4, unless otherwise stated. A bacterial culture sample adjusted to OD_600_ of 1 was harvested by centrifugation and served as the whole cell sample. Simultaneously, 13 mL of culture was centrifuged at 4 000 *g* for 20 min at 4°C to separate the bacterial cells from culture supernatant. 10 ml of the supernatant was transferred into falcon tubes and centrifuged again; 7 mL of the uppermost supernatant was transferred into new tubes and centrifuged. To 1.8 mL supernatant fraction, we added 200 μl trichloroacetic acid to precipitate proteins overnight at 4°C. The protein precipitate was centrifugated at 16 000 *g* for 30 min at 4°C and washed with cold 90% (v/v) acetone before further centrifugation. After removing the supernatant, the washed pellet was air-dried for 30 min and resuspended in 1x Laemmli buffer to an OD_600_ equivalent of 10.

### Western blot analysis and SDS-PAGE

For SDS-PAGE analysis, cell extracts were loaded onto SDS polyacrylamide gels, migrated and transferred to a nitrocellulose membrane at 3 mA/cm^2^. Following transfer, membranes were incubated overnight in blocking buffer (5% milk powder, 0.1% Tween 20 in Tris-buffered saline, pH 8.0). Polyclonal antibodies against VgrG1abc were used at a dilution of 1:1000 [37], against the C-terminal extension domain of VgrG4b (VgrG4b^C^) at 1:1000, against Hcp1 at 1:1000 [37], against Hcp2 at 1:1000 [46], against Tse3 at 1:500 [37]. Monoclonal antibodies against the β subunit of RNA polymerase (RpoB, NeoClone) were used at 1:5000. Secondary antibodies conjugated to horseradish peroxidase were used at a dilution of 1:5000. Western blots were developed using Super-Signal West Pico Chemiluminescent Substrate (Pierce) and visualized on a LAS3000 Fuji Imager.

### Interbacterial competition assays

Interbacterial competition assays were conducted on solid media due to the contact-dependent killing of the T6SS. Prey *P*. *aeruginosa* strains contained the Mini-CTX-*lacZ* integrated at the *att* site, consequently giving rise to blue colonies on X-gal-containing media. Overnight cultures in TSB were collected by centrifugation at 8 000 *g* for 3 min before washing twice in 1 ml sterile PBS and normalised to OD600 of 3.0. The OD_600_ was measured again for confirmation and 100 μl of attacker and 20 μl prey strains were mixed. This mixture was centrifuged at 8 000 *g* for 3 min and 20 μl supernatant was removed to result in a competition mixture ratio of 5:1 of attacker and prey strains. 5 μl of each competition mix was spotted in duplicates onto LB-agar, the spots well dried and the Petri dish lids were secured using parafilm M (Bemis). Competition plates were inverted and incubated at 37°C for 5h for H1-T6SS-inducive killing or at 25°C for 24h for H2-T6SS-inducive killing.

The input competitions were serially diluted to 10^−7^, plated on selective media for both attacker and prey (LB agar with 100 μg mL^-1^ X-gal for blue/white *P*. *aeruginosa* prey/attacker differentiation) and grown overnight at 37°C to confirm the input ratios. Competition spots were gathered using 5 μl inoculation loops (VWR) and resuspended in 1 ml PBS. The competition output mixture was serially diluted to 10^−7^, plated on selective media and grown overnight at 37°C similarly to the input. Both attacker and prey colony forming units were enumerated on both input and output dilution plates. All competition assays were repeated three times unless otherwise stated and the mean cfu of survived prey strains obtained from all experiments with the standard deviation was plotted.

## Results

### A “canonical” VgrG can drive the secretion of an effector domain from an “evolved” VgrG

Several VgrG proteins carry C-terminal extension domains with effector functions and are thus called “evolved” VgrGs [31, 33–35]. In *P*. *aeruginosa* PAO1 only one *vgrG* gene out of ten found on the genome was identified to encode an evolved VgrG, namely VgrG2b (S2A Fig, top panel). VgrG2b carries a DUF2345 domain (bright green) as well as an about 260 aa long C-terminal extension domain (VgrG2b-CT, red underlined) that can be further distinguished into a transthyretin- (TTR-) like domain (bright green) and an enzymatic Zn-dependent metallopeptidase domain (red) [18, 33]. It was shown that the VgrG2-CT could have dual and trans-kingdom activity since it is translocated into HeLa cells and facilitates actin-dependent internalisation of *P*. *aeruginosa* [33] while it can intoxicate bacterial prey cells when delivered to the periplasm where it impacts peptidoglycan biogenesis and cell division [18].

**Fig 1:**
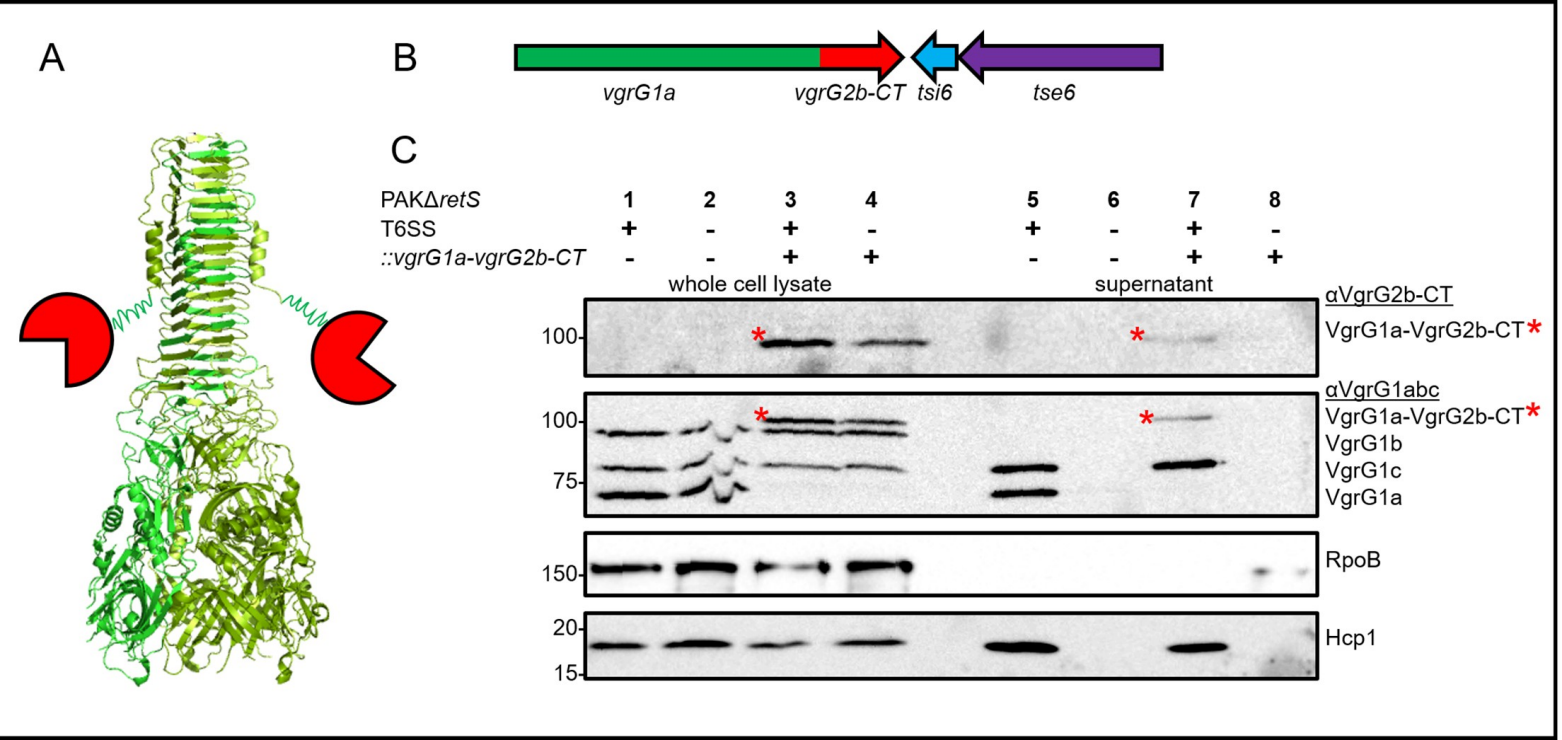
VgrG1a-VgrG2b-CT is secreted into the supernatant. (A) Schematic of an evolved VgrG. The green torch-like structure corresponds to the gp5-gp27-like domains, while the red circles depict the toxic C-terminal extension domains. (B) Design of *P*. *aeruginosa* PAK mutants in which the STOP codon of *vgrG1a* (green) was replaced on the chromosome with the gene sequence encoding VgrG2b-CT (red), while the downstream region of *vgrG1a* was unaltered. See S2 Fig for more details. (C) Representative figure of a western blot from a secretion assay using PAKΔ*retS* carrying an active (+) or inactive (-) H1-T6SS. Strains produce native VgrG1a (-) or the chimeric fusion VgrG1a-VgrG2b-CT (+). Antibodies (from top to bottom) against VgrG2b-CT, VgrG1abc, RpoB and Hcp1 were used as indicated on the right. Highlighted with red asterisks are the bands corresponding to the chimera VgrG1a-VgrG2b-CT.

Since delivery of VgrG2b-CT is dependent on VgrG2b as a vehicle in PAO1, we wanted to unravel whether a canonical VgrG could be used to deliver VgrG2b-CT and, thus, whether canonical VgrGs are transformable into “evolved” VgrGs, which has not yet been assessed. As a vehicle, we chose the canonical *P*. *aeruginosa* VgrG1a that has no C-terminal extension domain (S2A Fig) and whose secretion is significantly elevated in *P*. *aeruginosa* PAK carrying a deletion for the *retS* gene [37]. Hence, we designed a strategy to fuse VgrG2b-CT covalently at the C terminus of VgrG1a in PAK by replacing on the genome the STOP codon of *vgrG1a* with the gene sequence corresponding to *vgrG2b-*CT (S2B Fig). This led to the production of a chimera of VgrG1a carrying the C-terminal “evolved” domain of VgrG2b (VgrG1a-VgrG2b-CT, Fig 1A and 1B) which was engineered in a parental strain and its isogenic T6SS-inactive strain deleted for *tssB1* [37].

To investigate whether the VgrG1a-VgrG2b-CT chimera is transported into the bacterial culture supernatant, secretion assays were performed (Fig 1C). Remarkably, the chimeric VgrG1a-VgrG2b-CT could be detected at approximately 100 kDa when using a specific antibody directed against VgrG2b-CT (Fig 1C, top panel, lane 3, asterisk). When probed with an antibody against VgrG1abc (Fig 1C, second panel) it could be seen that the original VgrG1a is no longer detectable while a shift towards the 100 kDa protein is now clearly visible (Fig 1C, second panel, asterisk, lane 3), which overall confirms the identity and size of the VgrG1a-VgrG2b-CT chimera. The same band could be detected in the supernatant fractions of T6SS-active strains (Fig 1C, top and second panels, asterisks, lane 7), but not of T6SS-defective strains lacking the sheath protein TssB (Fig 1C, top and second panels, lane 8). The specificity of the T6SS-dependent behaviour is also shown by monitoring Hcp1 secretion (Fig 1C, bottom panel) and the absence of RNA polymerase RpoB in the culture supernatant (Fig 1C, third panel). This led us to the conclusion that VgrG1a can be modified to secrete a heterologous effector domain across the bacterial cell envelope in a T6SS-dependent manner, and that canonical proteins do not seem to have restrictions to bear extra domains at their C termini. Furthermore, it shows, that the extension domain of VgrG2b, VgrG2b-CT, does not necessarily require VgrG2b as its vehicle for delivery.

### The unrelated passenger protein Bla_TEM-1_ can be delivered *via* the T6SS

**Fig 2:**
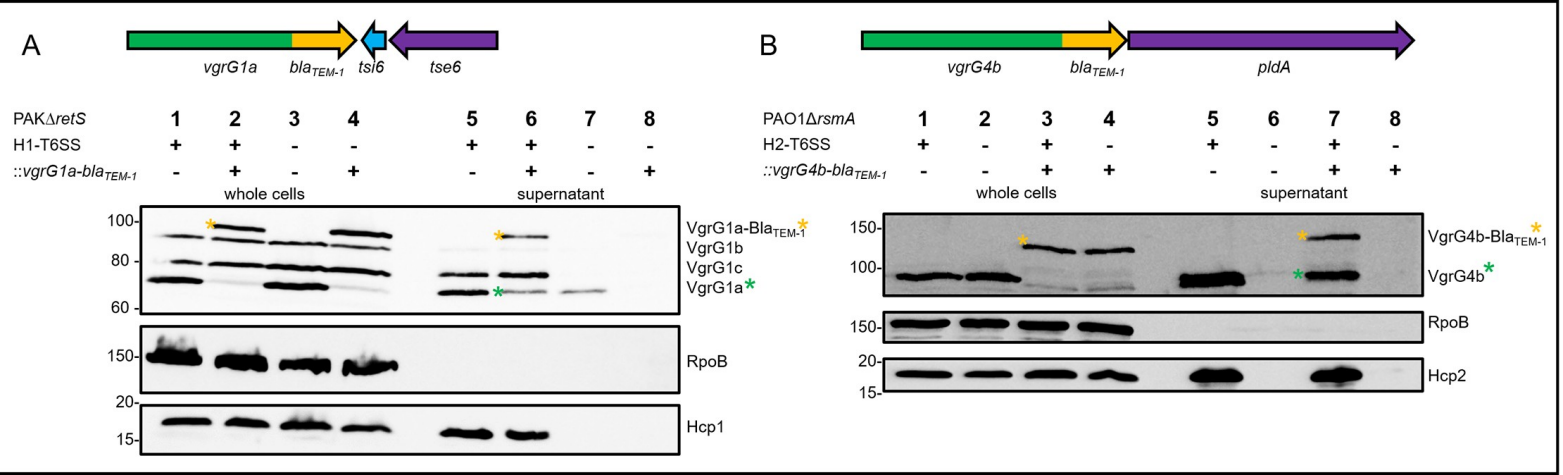
VgrG-Bla_TEM-1_ fusions can be secreted into the supernatant. Top panels: the gene encoding Bla_TEM-1_ (orange) was fused to (A) *vgrG1a* and (B) *vgrG4b*, while each downstream region on the chromosome was left unaltered. Bottom panels: representative figures of western blots of secretion assays using (A) PAKΔ*retS* carrying an active (+) or inactive (-) H1-T6SS and expressing native VgrG1a (-) or the chimeric VgrG1a-Bla_TEM-1_ (+), or (B) PAO1Δ*rsmA* carrying an active (+) or inactive (-) H2-T6SS encoding VgrG4b-Bla_TEM-1_ (+). Antibodies used (from top to bottom) are against (A) VgrG1abc, RpoB and Hcp1 or (B) VgrG4b^C^, RpoB and Hcp2, as indicated on the right. Highlighted with orange asterisks are bands corresponding to (A) VgrG1a-Bla_TEM-1_ or (B) VgrG4b-Bla_TEM-1_, while highlighted with green asterisks are degradation products corresponding to (A) native VgrG1a or (B) native VgrG4b.

Next, we wanted to assess whether a T6SS-unrelated passenger domain, the β-lactamase Bla_TEM-1_, can be channelled into the T6SS secretion machine of *P*. *aeruginosa*. For this, we chose the two canonical VgrGs, VgrG1a and VgrG4b (S2A Fig), that are associated with the H1-T6SS [37] and H2-T6SS [1], respectively. While the majority of H1-T6SS research was conducted in PAKΔ*retS* grown at 37°C [37], the H2-T6SS has been investigated in PAO1Δ*rsmA* grown at 25°C [1], which is why we used these two different strains in these experiments. We fused the mature Bla_TEM-1_, lacking its peptide signal, covalently to both VgrGs in a strategy similar to the one described for the VgrG1a-VgrG2b-CT fusion above (Fig 2, upper panels). To assess T6SS-dependent secretion, we additionally used an H1-T6SS- inactive strain, which is deleted for *tssB1* [37], and an H2-T6SS mutant deleted for *tssB2* [1].

Secretion assays of T6SS-active and T6SS-inactive strains encoding the VgrG1a-Bla_TEM-1_ (Fig 2A) and the VgrG4b-Bla_TEM-1_ (Fig 2B) fusions were performed under H1-T6SS and H2-T6SS-inducing conditions [1, 37], respectively. Using antibodies against VgrG1abc and VgrG4b we detected the original proteins at 72 kDa and 90 kDa (Fig 2, upper panels, lanes 1), respectively. The Bla_TEM-1_ domain adds 28 kDa, leading to the chimeric VgrG-Bla_TEM-1_ fusions to appear as larger products of expected molecular weight, marked with orange asterisks (Fig 2A, lane 2, top panel and Fig 2B, lane 3, top panel, respectively), while the two original proteins, VgrG1a and VgrG4b are no longer visible. The VgrG1a-Bla_TEM-1_ (Fig 2A, lane 6, top panel) as well as the VgrG4b-Bla_TEM-1_ (Fig 2B, lane 7, top panel) proteins can be found in the supernatant fractions of T6SS-active strains (orange asterisks), but not of T6SS-inactive strains (Fig 2, each lane 8). It is noteworthy, that in both cases, bands corresponding to the original VgrG1a and VgrG4b, respectively, are observable in the supernatant fractions of T6SS-active strains (green asterisks in Fig 2A, lane 6, top panel, and Fig 2B, lane 7, top panel, respectively). As these are not seen in the cell fraction (Fig 2A, lane 2, top panel and Fig 2B, lane 3, top panel), it is proposed that these bands might result from degradation and proteolytic cleavage of the Bla_TEM-1_ domain from the chimera once released extracellularly. Thus, we showed that a canonical VgrG can secrete a T6SS-unrelated domain when covalently fused.

### Grafting an Hcp-dependent effector on a canonical VgrG

A number of T6SS effectors were proposed to be delivered within the Hcp tube. In *P*. *aeruginosa* these are Tse1, Tse2 and Tse3 which are bound within the Hcp1 ring [23, 29]. Tse1 and Tse2 are thought to fold into small enough proteins to fit the Hcp1 ring cavity, while Tse3 adopts a lengthy structure to plug into the lumen of Hcp1 hexamers [47–50]. For our study, we chose the Hcp1-dependent effector Tse2, whose stability highly depends on its interactions with Hcp1 [29]. Thus, we challenged the VgrG shuttle concept by the attempt to deliver an Hcp-dependent T6SS effector as an extension domain of VgrG and thus uncouple the T6SS effector from its original Hcp vehicle.

**Fig 3:**
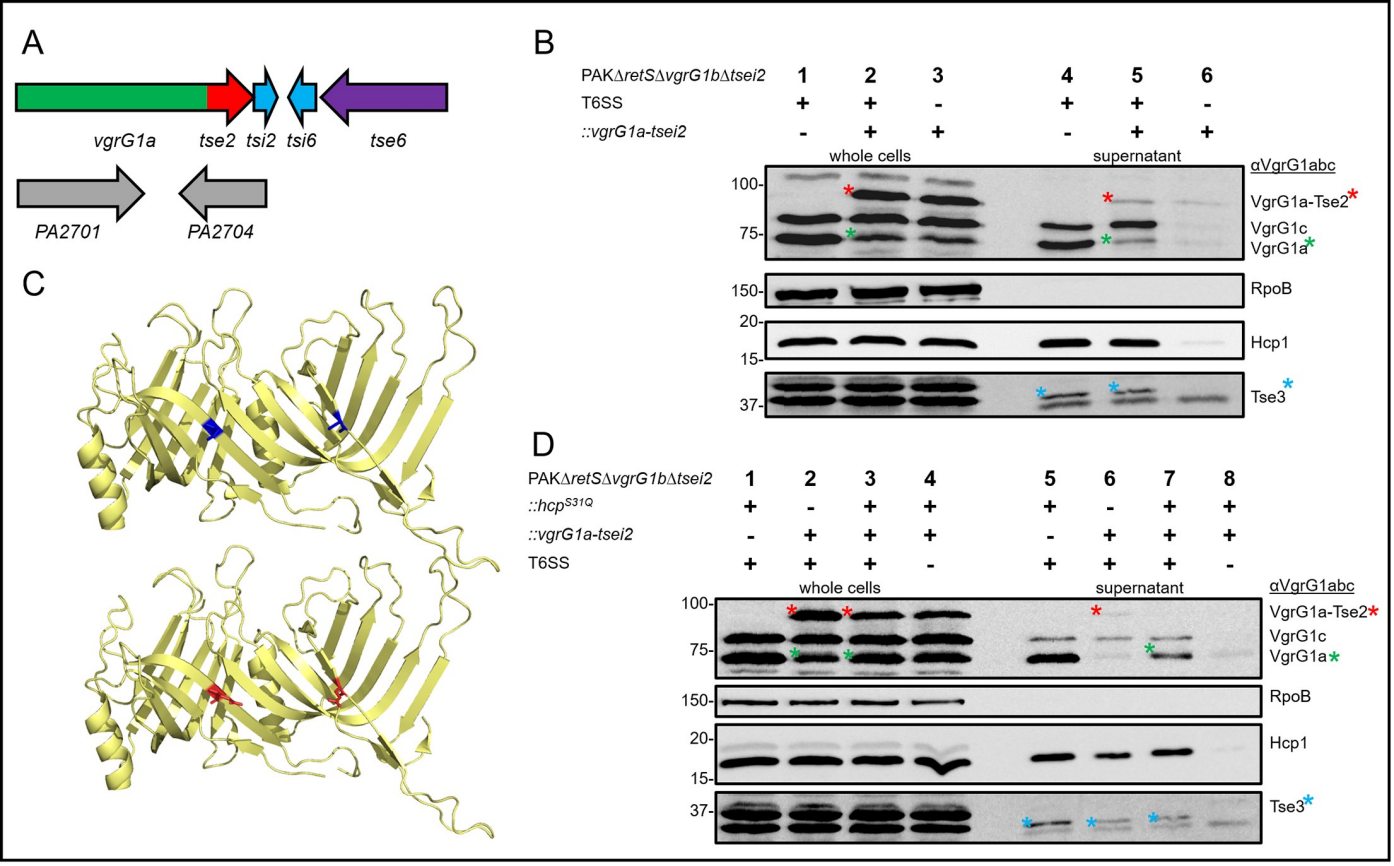
VgrG1a moderately secretes covalently fused Tse2. (A) Design of mutants in which the STOP codon of *vgrG1a* was replaced on the chromosome with the gene sequence encoding *tse2-tsi2* (red-cyan) while native *tse2-tsi2* (*PA2702-PA2703*) was deleted. (B, D) Representative western blots of secretion assays using PAKΔ*retS*Δ*vgrG1b*Δ*tsei2* expressing native VgrG1a (-) or the chimeric fusion VgrG1a-Tse2 (+). Strains in (B) produce an active (+) or inactive (-) H1-T6SS, while strains in (D) additionally encode native Hcp (-) or mutant Hcp^S31Q^ (+). Antibodies used (from top to bottom) are against VgrG1abc, RpoB, Hcp1 and the Hcp1-dependent effector Tse3 as indicated on the right. Highlighted with red asterisks are bands corresponding to VgrG1a-Tse2, while highlighted with green asterisks are degradation products likely corresponding to native VgrG1a and blue asterisks correspond to the Tse3 effector. (C) Hcp1 dimer (pdb: 1Y12) in yellow and highlighted inside the Hcp1 lumen in blue are the native serine (upper panel) or in red the mutated glutamine (bottom panel).

To monitor whether the canonical VgrG1a would be able to deliver Tse2 into the supernatant or bacterial preys, we followed the previous grafting strategy and fused *tse2* to *vgrG1a* by allelic replacement of the *vgrG1a* STOP codon directly on the chromosome (Fig 3A). To avoid toxicity against sister cells, the cognate immunity *tsi2* was also cloned so that this gene now lies immediately downstream of the *vgrG1a-tse2* allele in the *vgrG1a* cluster. At the same time, the native *tse2tsi2* (*tsei2*) locus was deleted (Fig 3A). The VgrG1a-Tse2 chimera has an expected molecular size of about 91 kDa, which would then be similar to VgrG1b. Because the VgrG1abc-antibody also recognises VgrG1b [37], the *vgrG1a-tse2* chimera was engineered in mutant strains lacking the *vgrG1b* gene.

Western blot analysis using an antibody against VgrG1abc shows that only VgrG1a and VgrG1c are detectable in whole cells of the parental strain (Fig 3B). For strains carrying the *vgrG1a-tse2* fusion, an additional band corresponding to VgrG1a-Tse2 is readily visible (Fig 3B, lane 2, red asterisk), while a band corresponding to the original VgrG1a is still observable (Fig 3B, lane 2, green asterisk). This suggests that the chimera VgrG1a-Tse2 is expressed, but likely prone to proteolytic cleavage. VgrG1a-Tse2 is further detectable in the supernatant of T6SS-active cells (Fig 3B, lane 5, red asterisk) suggesting chimera secretion, while a band corresponding to VgrG1a itself is also detectable in the supernatant fraction (Fig 3B, lane 5, green asterisk). However, it is not clear whether presence of VgrG1a in the supernatant results from cleavage of the chimera after secretion or, most likely, whether intracellularly cleaved VgrG1a is secreted. Importantly, in the supernatant fraction of T6SS-defective strains (Fig 3B, lane 6), which is lacking the essential component TssB1, the bands corresponding to VgrG1a-Tse2, VgrG1c, VgrG1a and Hcp1 are mostly absent although residual amount is detected. Yet, Tse3, another Hcp1-dependent effector, was used as an additional control for secretion (Fig 3B, bottom panel, upper band with blue asterisk), and was not detected in the supernatant of a T6SS inactive strain. Finally, the intracellular control RpoB (Fig 3B, second panel) is not detectable in the supernatant indicating that cell lysis is unlikely and presence/absence of proteins in the supernatant is thus T6SS-dependent.

A previous study reporting the interaction between Hcp1 and Tse2 [29] showed how stability and delivery of Tse2 is highly dependent on Hcp1 and pinpointed key interaction residues on the inside of the Hcp1 lumen (Fig 3C). A range of highly conserved residues within the Hcp1 lumen appeared to be essential for interactions with Tse2 and mutation in each of these residues confirmed that Tse2 stability and secretion were drastically impaired [29]. In particular substitution of Serine (S) at position 31 for Glutamine (Q), yielding Hcp1^S31Q^, resulted in a huge decrease in the amount of intracellular Tse2 [29] (Fig 3C). Here, we used a similar background to assess how this residue might impact secretion of the VgrG1a-Tse2 chimera so that we repeated all above secretion assays including mutants expressing Hcp1^S31Q^ (Fig 3D).

Secretion of VgrG1a, VgrG1c, Hcp1 or Tse3 was not affected when cells contain the *hcp1*^*S31Q*^ variant (Fig 3D, lane 5). This is in good agreement with the previous study, in which it was shown that Tse3 secretion was not affected by the *hcp1*^*S31Q*^ variant mutation [29]. In a context where *vgrG1a* was replaced with *vgrG1a-tse2* (Fig 3D, lane 2), the abundance of the band corresponding to VgrG1a-Tse2 is higher in a parental background as compared to the *hcp1*^*S31Q*^ mutation (Fig 3D, top panel, compare lanes 2 and 3, red asterisk). This is likely caused by the fact that Tse2 no longer interacts with Hcp1^S31Q^ leading to destabilisation of the effector with subsequent degradation. The reduced amount of VgrG1a-Tse2 is also reflected by the absence of detectable secretion of the full-length chimera (Fig 3D, lane 7, top panel) in contrast to the parental background (Fig 3D, lane 6, top panel, red asterisk). Yet, in strains containing the *hcp1*^*S31Q*^ mutation (Fig 3D, lane 7, top panel, green asterisk), a rather intense band corresponding to VgrG1a is visible in the supernatant fraction of *hcp1*^*S31Q*^ mutants, as compared to the wild-type background. This suggests that either the secreted chimera is unstable or that the truncated VgrG1a from the VgrG1a-Tse2 chimera in the cell is efficiently secreted.

Overall, we conclude that the VgrG1a-Tse2 chimera can be partially secreted in a T6SS-dependent manner but restricted by the chaperoning function of Hcp1 for Tse2. It remains further elusive whether binding of the Hcp1 component to the chimera and at the tip of the T6SS is occurring prior and during the secretion process.

### Effector domains fused to a canonical VgrG are not efficiently delivered into target cells

**Fig 4:**
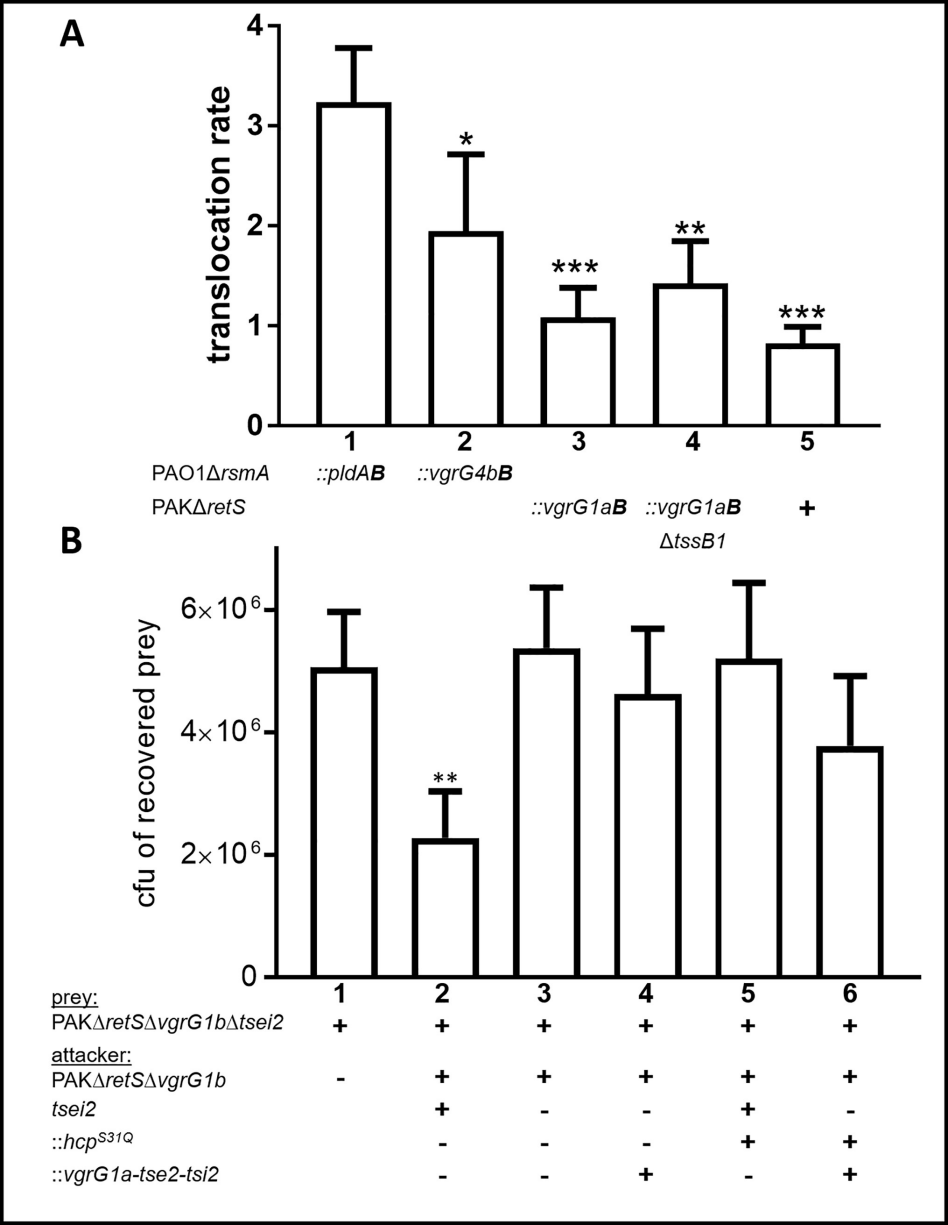
VgrG1a cannot deliver covalently fused effector domains into target cells. (A) HeLa cells were infected with *P*. *aeruginosa* strains for 3 h, washed and incubated with CCF2/AM substrate for 90 min. After extensive washing, emission of cells was measured. The translocation rate (emission of blue fluorescence/emission of green fluorescence) is expressed for each sample in relation to the emission ratio of uninfected cells [54]. A high translocation rate was observed for the positive control PldA-Bla_TEM-1_ (::*pldA****B***, lane 1), but not for the negative controls VgrG1a-Bla_TEM-1_ (::*vgrG1a****B***) from T6SS-deficient strains (lane 4) or when no chimera is present PAKΔ*retS* (+, lane 5).The positive control PldA-Bla_TEM-1_ (::*pldA****B***, lane 1) was translocated, but not the negative control VgrG1a-Bla_TEM-1_ (::*vgrG1a****B***) from T6SS-deficient strains (lane 4). An ordinary one-Way ANOVA analysis with Dunnett’s multiple comparisons test of three independent experiments was conducted on the data set obtained from the positive control with *** p<0.001. (B) Bacterial competition represented by plot from four independent experiments of recovered cfu of the prey strain PAKΔ*retS*Δ*vgrG1b*Δ*tsei2*::*lacZ* after contact with the attacker strain that encodes Tse2-Tsi2 (+), Hcp1^S31Q^ (+) or the chimera VgrG1a-Tse2 (+), as indicated. Spots were incubated for 5 h at 37°C in a 5:1 ratio. One-Way ANOVA analysis with Dunnett’s multiple comparisons test was conducted on data set obtained from recovered prey on their own with ** p<0.01.

We have shown that Bla_TEM-1_ can be secreted when fused to VgrG1a and VgrG4b, while Tse2 is not effectively released into the supernatant as a VgrG1a-Tse2 chimera. We further asked whether these fusions could be delivered into target cells. Bla_TEM-1_ is a common reporter enzyme to monitor translocation into eukaryotic cells [51, 52], while Tse2 is a toxin with its final destination being bacterial prey cells [53]. Hence, it was assessed whether VgrG1a or VgrG4b could fulfil a role that will drive Bla_TEM-1_ or Tse2 all the way from the producing bacteria into the cytosol of HeLa cells or into bacterial competitors (Fig 4).

The enzymatic activity of Bla_TEM-1_ is the readout for a cell-based translocation assay as described previously [55]. As a positive control for the experimental setup, a fusion between Bla_TEM-1_ and the T6SS effector PldA was engineered, since it has been shown before that a PldA-Bla_TEM-1_ fusion is translocated into infected HeLa cells in a T6SS-dependent manner [52], which we could confirm here (Fig 4A, lane 1). Our negative controls are either a mutant not encoding a Bla_TEM-1_ (Fig 4A, lane 5) or a non-deliverable VgrG1a-Bla_TEM-1_ produced in a T6SS-defective mutant (Fig 4A, lane 4). The test strains that express either VgrG4b-Bla_TEM-1_ (Fig 4A, lane 2) or VgrG1a-Bla_TEM-1_ (Fig 4A, lane 3) display a significant difference as compared to the positive control (* p < 0.5; *** p < 0.001) but no significance as compared to the negative controls, suggesting that no Bla_TEM-1_ translocation occurred. Results obtained here reflect that the fusion proteins were unable to puncture the eukaryotic cell membrane, or that the VgrG chimera falls out of the device before it could be engaged in such process. This could be a reasonable hypothesis for VgrG1a-Bla_TEM-1_, which is entirely artificial. However, in the case of VgrG4b-Bla_TEM-1_, it could have been expected to work since VgrG4b is the cognate VgrG for PldA, which we showed here is efficiently delivered as a PldA-Bla_TEM-1_ chimera (Fig 4A) [1, 24].

Next, we performed a bacterial competition assay [56] to evaluate whether Tse2 can reach its target in a T6SS-dependent manner but irrespective of its delivery mode. This will also demonstrate that a canonical VgrG, such as VgrG1a, is not only able to puncture the cell envelope of the producing cell but can also puncture bacterial prey cells. For effective intra-bacterial killing assays, *P*. *aeruginosa* prey cells were constructed that lack *vgrG1b* and were sensitive to Tse2 by lacking the effector gene *tse2* and its cognate immunity gene *tsi2*. The attacking strains expressed either VgrG1a or the VgrG1a-Tse2 fusion (Fig 4B). When the parental strain (PAKΔ*retS*Δ*vgrG1b*, Fig 4B, lane2) is the attacker, it effectively outcompeted the prey strain, however a strain expressing VgrG1a-Tse2 did not (Fig 4B, lane 4). This suggests that VgrG1a-Tse2, even though moderately released into the supernatant, cannot be driven as a chimera all the way into prey cells. Furthermore, strains that additionally harbour the *hcp*^*S31Q*^ mutation do not have a competitive advantage towards the prey cell (Fig 4B, lane 6), corroborating our results from the secretion assay. These results imply that neither VgrG4b nor VgrG1a can deliver their covalently fused extension domains into target cells.

## Discussion

Using bacterial protein secretion systems as a shuttle to deliver proteins in a targeted manner has been considered for many years. In particular, it was previously attempted to use the type III secretion system to deliver specific molecules into an appropriate environment. One example is the T3SS-dependent delivery of angiogenesis inhibitors by *Salmonella*, which proved significant anti-tumoral activity on mice colon carcinoma [57]. As such, bacterial secretion devices able to deliver proteins directly into the cytosol of human cells are considered the shuttles of choice when developing targeted medicine strategies [58]. The T6SS also injects toxins into bacterial preys and might thus be considered as a strategy to counteract pathogen infections if used by commensal bacteria present in an infected host [59]. Here, we developed this idea of the T6SS as a shuttle. The prerequisite to any future potential biotechnological applications, which is not the purpose of the present study, is to manipulate the T6SS and assess whether secretion/delivery of chosen effectors can be eased when these are connected to the VgrG tip. We could show that a canonical VgrG can be modified so that it is able to deliver an effector domain across the bacterial membrane.

Previous studies investigated secretion and translocation of evolved VgrGs (S3A Fig). Some studies used the β-lactamase as a reporter to follow the fate of VgrG chimera by monitoring translocation into eukaryotic cells [31, 35, 51]. In these cases, the β-lactamase domain was either used to extend an evolved VgrG or to substitute the extension domain thus mainly proving that the effector domain is not essential for VgrG delivery [31] (S1 Table). Another study previously showed that the covalent fusion of PldA to its cognate VgrG4b resulted in its secretion and delivery into target cells [24]. In the current study, we were able to corroborate these findings but expanded this knowledge to the concept that it is possible to extend a canonical VgrG with Bla_TEM-1_ or another effector domain and thus to create an artificial evolved VgrG.

**Fig 5:**
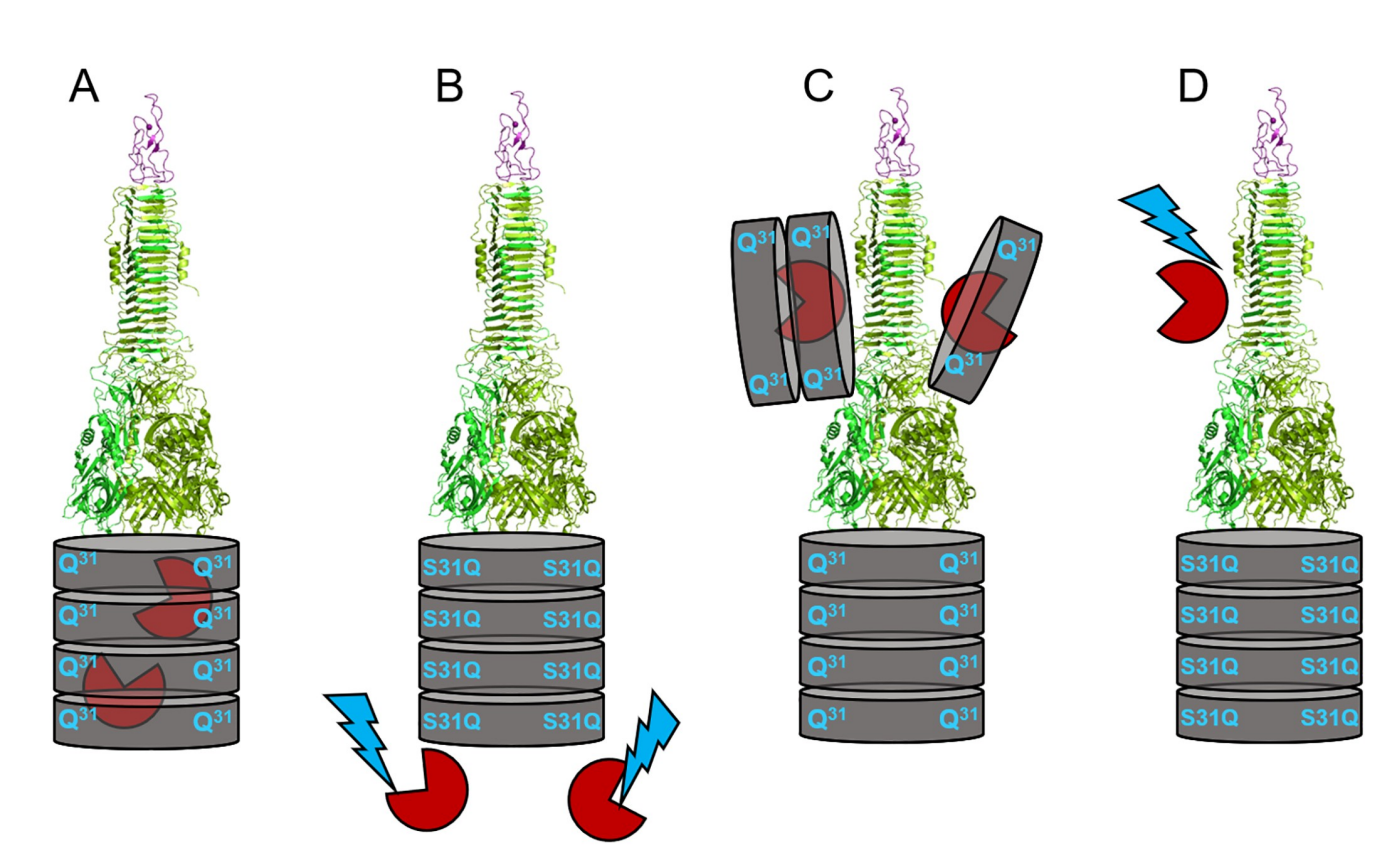
Model of interference of interactions between the Hcp1 ring and Tse2 as part of a VgrG1a-Tse2 shuttle. A: In the parental strain Tse2 (red) interacts with the key conserved residues (Q31 amongst others) within the lumen of Hcp1 (brown) leading to stabilisation and subsequent delivery of Tse2. A trimer of VgrG proteins (green, PDB: 4mtk) sits on top of the Hcp1 tube. B: One key residue within the Hcp1 lumen mediating interactions with Tse2 is mutated (S31Q). This prevents incorporation of Tse2 into the Hcp1 tube and renders it prone for proteolytic degradation (indicated by blue bolt). C: Tse2 is covalently fused to the canonical VgrG1a. Hcp1 molecules are recruited to the tip due to their high affinity for Tse2 leading to a bulky tip complex and blockage of the T6SS. D: One key residue is mutated within the Hcp1 lumen (S31Q) thus abolishing interactions with Tse2. This leads to destabilisation of Tse2 as C-terminal extension of VgrG1a, so that cleavage of Tse2 occurs and most likely degradation of the effector. WT VgrG1a is accumulated and can be incorporated into the T6SS complex and thus reaches the supernatant. This model is based on the data obtained in this current study and in the study by Silverman *et*. *al* [29].

In the attempt to modify the secretion route of Tse2, we corroborated previous evidence that Tse2 starkly requires Hcp1 for stability and for connecting it to the T6SS tip complex (Fig 5A and 5B) [29]. From our results, we could propose that Tse2, even when fused to another vehicle, like VgrG1a, still interacts with Hcp1. This may explain the low level of chimera secretion observed, likely due to steric hindrance when engaging into the secretion system and notably the T6SS baseplate and membrane complex. (Fig 5C). However, it seems that abolishing this interaction by mutating a conserved residue within the Hcp1 lumen still resulted in degradation of the chimera (Fig 5D). This fully supports the concept of Hcp1 being a chaperone and receptor for Tse2 [29] (S3B Fig) even when the latter is fused to a VgrG protein.

T6SS effectors were identified that connect to the T6SS tip *via* binding to C-terminal residues of a VgrG protein (S3C Fig) or to a transthyretin-like (TTR) domain within the C-terminus of a VgrG (S3D Fig). In many cases, binding to the last amino acids of a VgrG protein is mediated by a chaperone from the DUF4123 family, as for Tde1 from *A*. *tumefaciens* [23] or TseL in *V*. *cholerae* [25, 26]. Recent studies showed that Tle1 in *E*. *coli* as well as PldA and PldB in *P*. *aeruginosa* specifically interact with the C-terminal TTR-like domains of the respective upstream encoded VgrG1^*Eco*^, VgrG4b^*Paer*^ and VgrG5^*Paer*^ [19, 24]. Flaugnatti *et al*. also demonstrated that delivery of Tle1^*Eco*^ solely depends on the TTR-like domain of VgrG1^*Eco*^ [19]. Another recent study showed that by swapping the TTR-like domains between VgrG4b^*Paer*^ and VgrG5^*Paer*^, the specificity of the VgrG for their effectors would also be swapped [24].

In addition to loading an effector directly onto a VgrG protein, effectors can also be connected to PAAR proteins (S3E Fig). PAAR domains can be part of an effector and thus help loading the extended effector domain directly onto the spike complex [11]. The involvement of additional proteins was also shown, like DUF1795, namely Eag proteins [21, 27, 28, 60, 61] and DUF2169 proteins [23]. The Eag proteins EagR1 and EagR2 from *S*. *marcescens* were observed to specifically bind and likely stabilise the N-terminal regions of their cognate effectors Rhs1 and Rhs2, respectively [62] (S3E Fig). In contrast, the involvement of a DUF2169 protein in effector delivery was so far only shown for Tde2 delivery in *A*. *tumefaciens* [23]. However, the molecular function of a DUF2169 protein and its detailed role in mediating effector delivery remains elusive. Additionally, PAAR effectors were characterised that additionally contain a large Rhs core [20]. The structure of such Rhs core domain was solved and showed that it forms a shell-like shape enclosing the C-terminal effector domain [63]. Recently, it was also suggested that the C-terminal effector domain might be cleaved from the Rhs core before translocation [64]. However, the accommodation of this large protein around the T6SS tip complex and the molecular mechanisms of effector translocation remain to be further characterized.

The fact that effectors can be attached to the T6SS *via* different mechanisms highlights its versatility and suggested that it could be a useful potential vehicle to deliver a range of effectors into bacterial preys. However, details of the molecular delivery mechanisms are still unclear for many T6SS effectors and the diversity in branching effectors to the machine makes it also very complex in deciding what the best configuration for a vehicle/passenger could be. Here, we made use of the simplest known delivery mechanism by fusing effector domains to the structural component VgrG. Although we showed that this configuration is sufficient to secrete the effector domain outside of the producing cell, this occurs with variable efficacy while the delivery into a target cell is not guaranteed since none of our chimera could be efficiently injected into the intended target cell. It is not clear why this is the case, and one would need to try using other canonical VgrGs to assess whether it is a general problem or whether it is specific to VgrG1a. One possibility is that modifying the tip of the VgrG protein might destabilize the binding of the cognate PAAR protein [11]. Under such configuration, the PAAR association with the VgrG chimera might be preserved while the complex is embedded within the TssBC sheath/T6SS baseplate or the TssJLM membrane complex, and this would be sufficient to perforate the membrane of the producing cell. Yet, the VgrG/PAAR association might not be strong enough to allow penetration of the chimera into target cells once it is expelled from the attacker cell. In light of our data, the biotechnological applications of VgrG-shuttling are likely possible. As it stands, developing this type of approaches would require a better molecular understanding of the organization around the VgrG tip, so that further engineering may make effective the delivery into target cells.

## Supporting information

S1 Fig Strategy to construct chimeric *P*. *aeruginosa* mutants. A: Three DNA fragments were produced with primers exF and upR; upF and dnR; dnF and exR with upR and upF and dnR and dnF containing overlapping sequences. upF and dnR produce the fragment (blue) that would be introduced into the genome (yellow). B: Two consecutive overlap PCRs, ultimately using exF and exR, produce a fragment that was cloned into the suicide plasmid pKNG101 and introduced into the genome. After a double recombination event, presence of the chimeric gene was verified by PCR using exF and exR.
(TIF)

S2 Fig Design of VgrG-shuttles used in this study. A: Protein domains of the here used VgrG2b (PA0262), VgrG1a (PAK00309) and VgrG4b (PA3489). The N-terminal part of all three VgrGs are the gp5/gp27-like domains (greens) spanning the first approximately 610 aa. In VgrG2b, this region is followed by a DUF2345 and a transthyretin-like domain (grey-green) [33]. The C-terminal 170 aa comprise of the metallopeptidase domain (red). VgrG4b, as well, harbours a TTR-like domain [19]. B: Genetic environments of the *vgrG2b* and *vgrG1a* genes in *P*. *aeruginosa* PAO1 and PAK. The *vgrG2b* gene (green-red) in *P*. *aeruginosa* PAO1 is followed by the gene *PA0261* (cyan). In PAK, downstream of *vgrG1a* (green), *tse6* (dark red) and the immunity *tsi6* (cyan) are located.
(TIF)

S3 Fig Effector proteins can be connected to the T6SS tip *via* different mechanisms. (A) An effector domain is part of an evolved VgrG (green, model PDB: 4mtk) and is thus part of the VgrG spike tip. (B) T6SS effectors (red) are bound and stabilised by Hcp-hexamers (grey) and recruited towards the T6SS tip *via* assembly of the Hcp-tube. (C) An effector directly interacts with its cognate Tap protein (magenta) which stabilises it and recruits the effector to the T6SS tip. (D) An effector directly interacts with the C-terminal TTR-like domain (green arc) of a VgrG that both connects it to the T6SS tip and stabilises the effector protein. (E) An effector contains an N-terminal PAAR domain (purple) that associates at the VgrG trimer and the effector hydrophobic transmembrane domains are chaperones in the cytosol by specific Eag proteins (orange). In all cases, by propelling out the Hcp tube with the VgrG spike on top, the effector proteins or domains are translocated across the bacterial cell membranes.
(TIF)

S1 Information File RAW data of western blot images. The RAW images are present in the file in the order they occur in the document. The lanes are labelled with the strain names from which the samples derive. The antibody against which was blotted, is noted. Lanes that are not shown in the final document are marked.
(PDF)

S1 Table Overview of known evolved VgrGs from the literature and from the current study. Names of the VgrGs are adapted from the literature with superscripted abbreviations corresponding to the organism the VgrG is derived from (*Paer****—****P*. *aeruginosa; Vcho****–****V*. *cholerae; Ahyd****–****A*. *hydrophila; Bpse****–****B*. *pseudomallei; Ftul****–****Francisella tularensis*). If no organism is mentioned, its source is *P*. *aeruginosa*. Artificially chimeric VgrGs with fused effector domains are highlighted with a grey background. The table lists the enzymatic activities of the extension domains and their size. It further highlights whether the evolved VgrG was found secreted in the supernatant and whether translocation in either eukaryotic or prokaryotic cells was shown.
(DOCX)
