# Supplementary figures and images for: Manipulating the type VI secretion system spike to shuttle passenger proteins
Source: PLoS One. 2020 Feb 26;15(2):e0228941. doi: 10.1371/journal.pone.0228941 (PMC7043769; doi:10.1371/journal.pone.0228941)

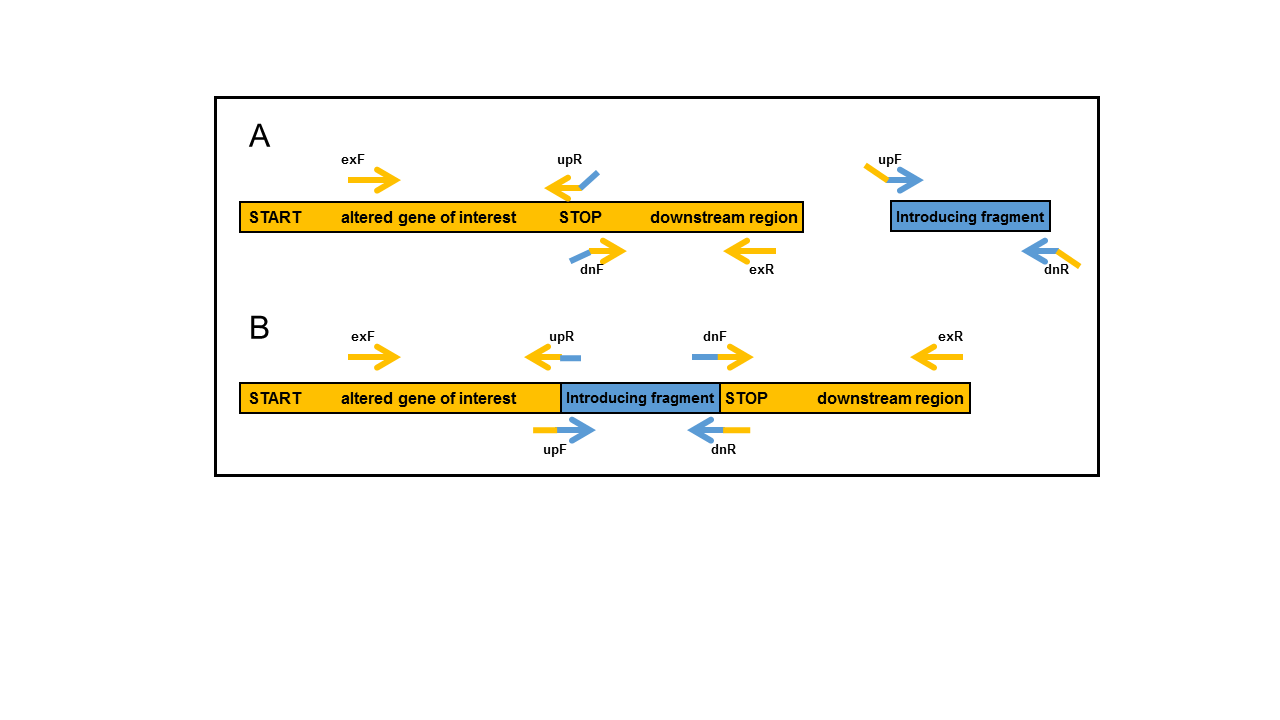

Supplement: S1 Fig — A: Three DNA fragments were produced with primers exF and upR; upF and dnR; dnF and exR with upR and upF and dnR and dnF containing overlapping sequences. upF and dnR produce the fragment (blue) that would be introduced into the genome (yellow). B: Two consecutive overlap PCRs, ultimately using exF and exR, produce a fragment that was cloned into the suicide plasmid pKNG101 and introduced into the genome. After a double recombination event, presence of the chimeric gene was verified by PCR using exF and exR. (TIF) [file pone.0228941.s001.tif]

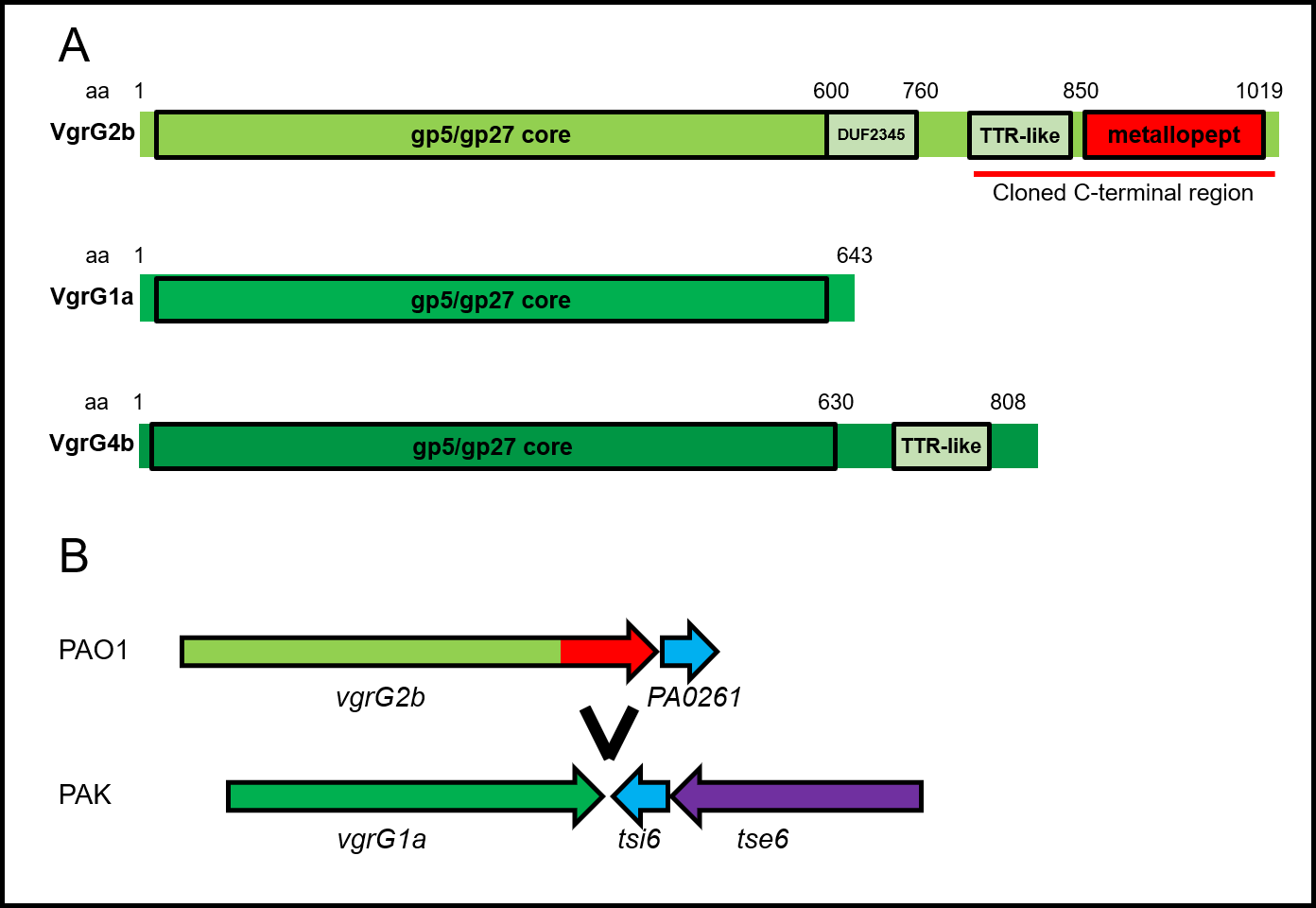

Supplement: S2 Fig — A: Protein domains of the here used VgrG2b (PA0262), VgrG1a (PAK00309) and VgrG4b (PA3489). The N-terminal part of all three VgrGs are the gp5/gp27-like domains (greens) spanning the first approximately 610 aa. In VgrG2b, this region is followed by a DUF2345 and a transthyretin-like domain (grey-green) [33]. The C-terminal 170 aa comprise of the metallopeptidase domain (red). VgrG4b, as well, harbours a TTR-like domain [19]. B: Genetic environments of the vgrG2b and vgrG1a genes in P. aeruginosa PAO1 and PAK. The vgrG2b gene (green-red) in P. aeruginosa PAO1 is followed by the gene PA0261 (cyan). In PAK, downstream of vgrG1a (green), tse6 (dark red) and the immunity tsi6 (cyan) are located. (TIF) [file pone.0228941.s002.tif]

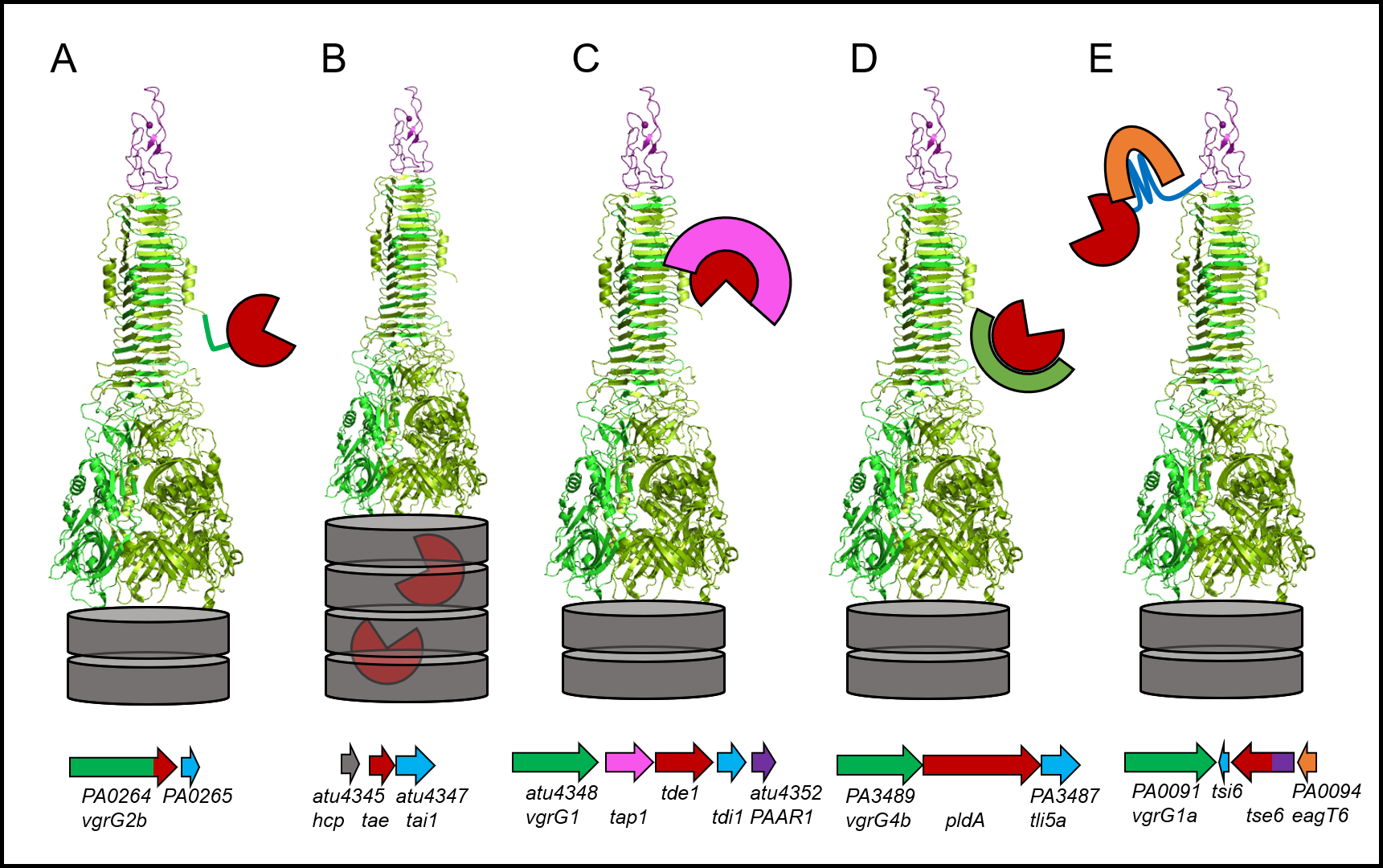

Supplement: S3 Fig — (A) An effector domain is part of an evolved VgrG (green, model PDB: 4mtk) and is thus part of the VgrG spike tip. (B) T6SS effectors (red) are bound and stabilised by Hcp-hexamers (grey) and recruited towards the T6SS tip via assembly of the Hcp-tube. (C) An effector directly interacts with its cognate Tap protein (magenta) which stabilises it and recruits the effector to the T6SS tip. (D) An effector directly interacts with the C-terminal TTR-like domain (green arc) of a VgrG that both connects it to the T6SS tip and stabilises the effector protein. (E) An effector contains an N-terminal PAAR domain (purple) that associates at the VgrG trimer and the effector hydrophobic transmembrane domains are chaperones in the cytosol by specific Eag proteins (orange). In all cases, by propelling out the Hcp tube with the VgrG spike on top, the effector proteins or domains are translocated across the bacterial cell membranes. (TIF) [file pone.0228941.s003.tif]
