# Supplementary material for: Manipulating the type VI secretion system spike to shuttle passenger proteins
Source: PLoS One. 2020 Feb 26;15(2):e0228941. doi: 10.1371/journal.pone.0228941 (PMC7043769; doi:10.1371/journal.pone.0228941)
Supplement: S1 Information File — The RAW images are present in the file in the order they occur in the document. The lanes are labelled with the strain names from which the samples derive. The antibody against which was blotted, is noted. Lanes that are not shown in the final document are marked. (PDF) [file pone.0228941.s004.pdf]

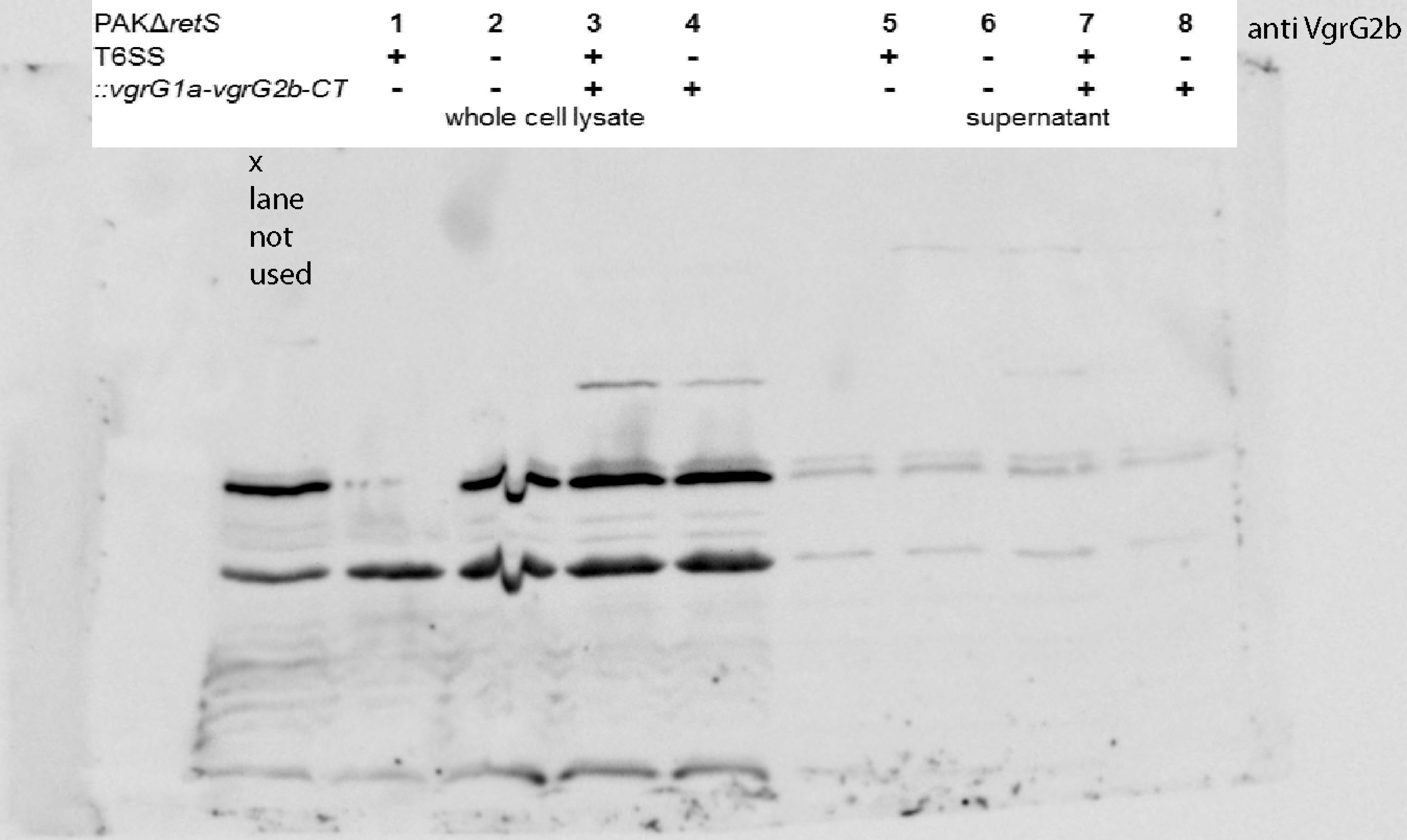

PAKΔretS 1 2 3 4 5 6 7 8
T6SS + - + - + - + -
::vgrG1a-vgrG2b-CT - - + + - - + +
whole cell lysate
supernatant
anti VgrG2b
x lane not used

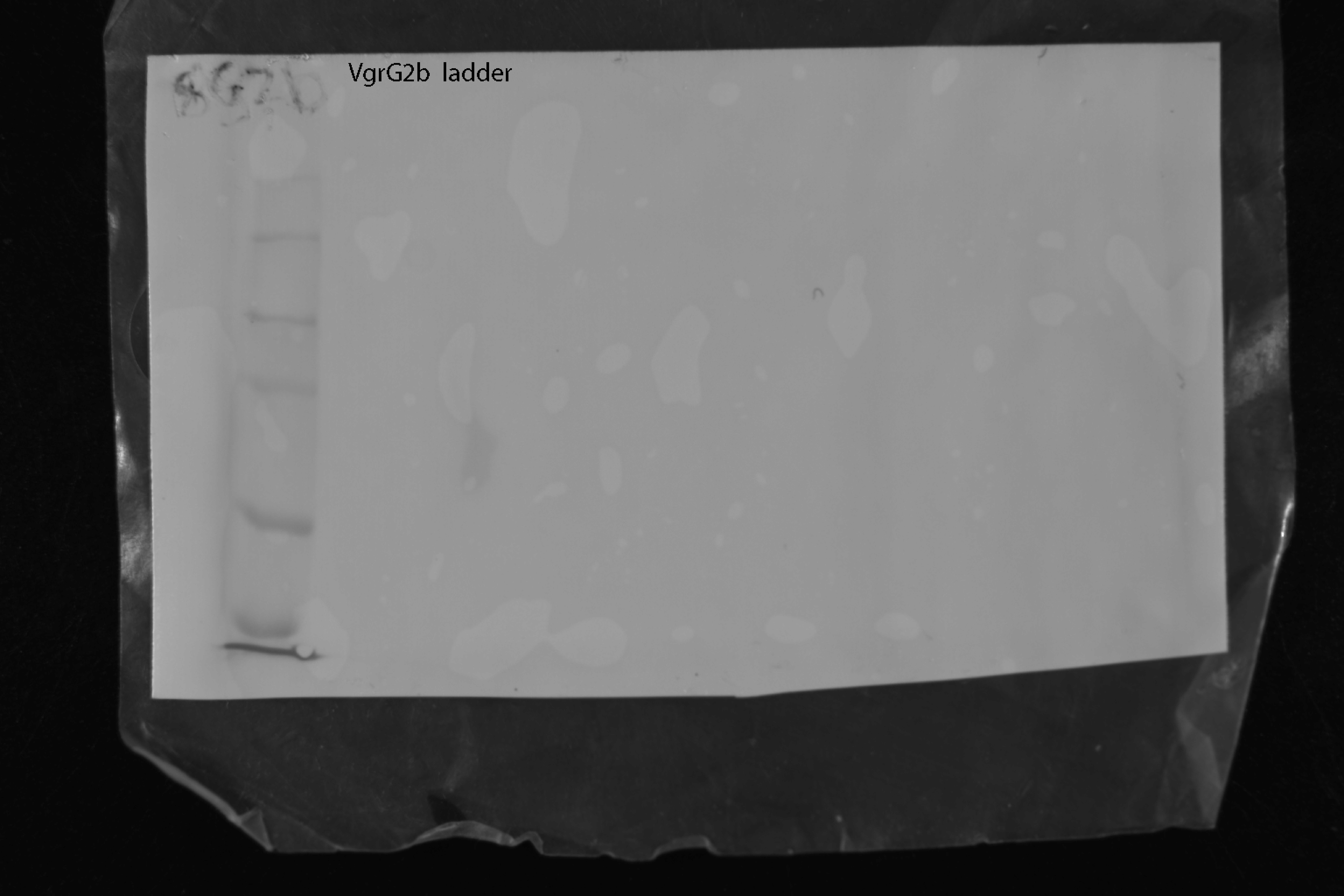
VgrG2b ladder

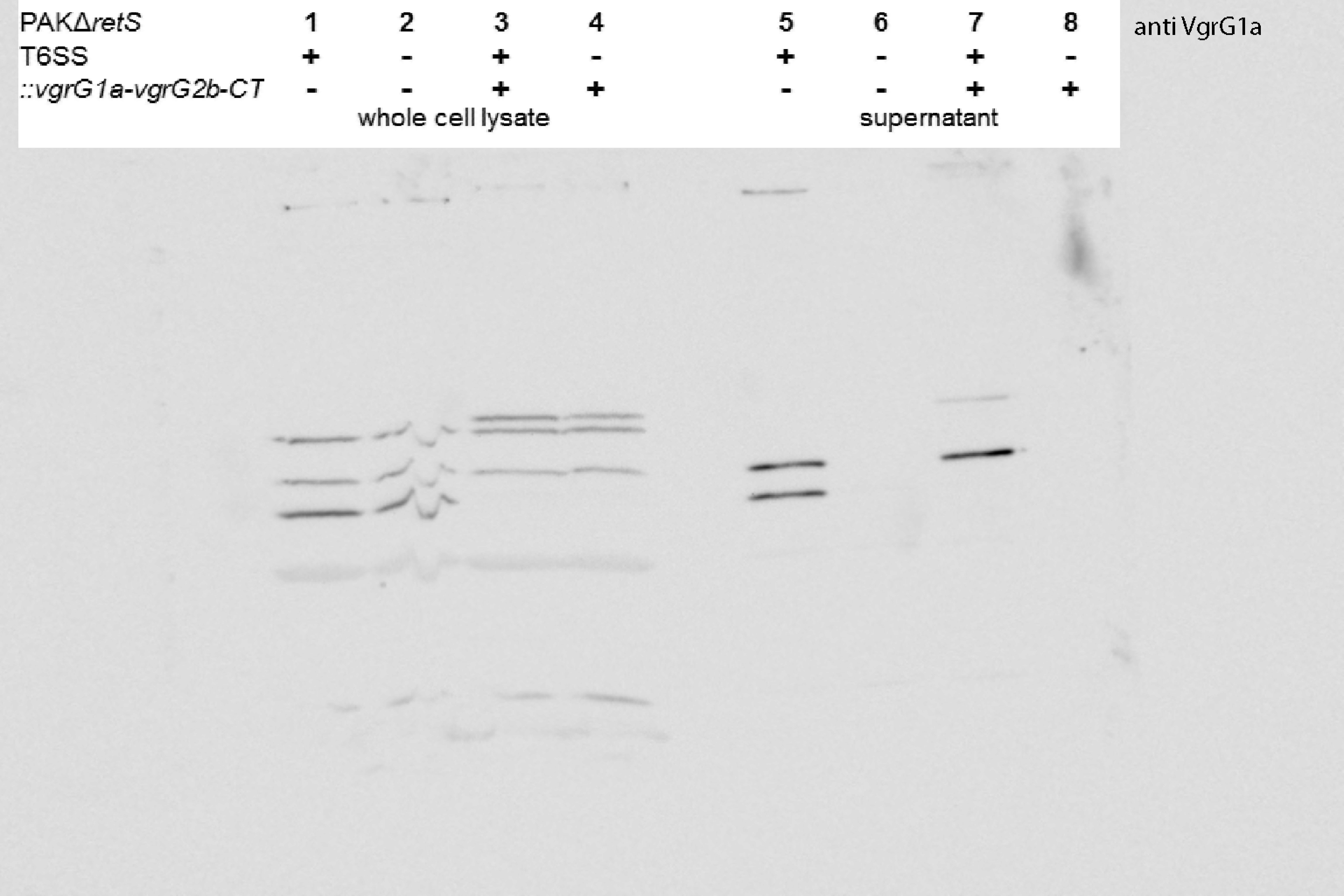
PAKΔretS 1 2 3 4 5 6 7 8
T6SS + - + - + - + -
::vgrG1a-vgrG2b-CT - - + + - - + +
whole cell lysate
supernatant
anti VgrG1a

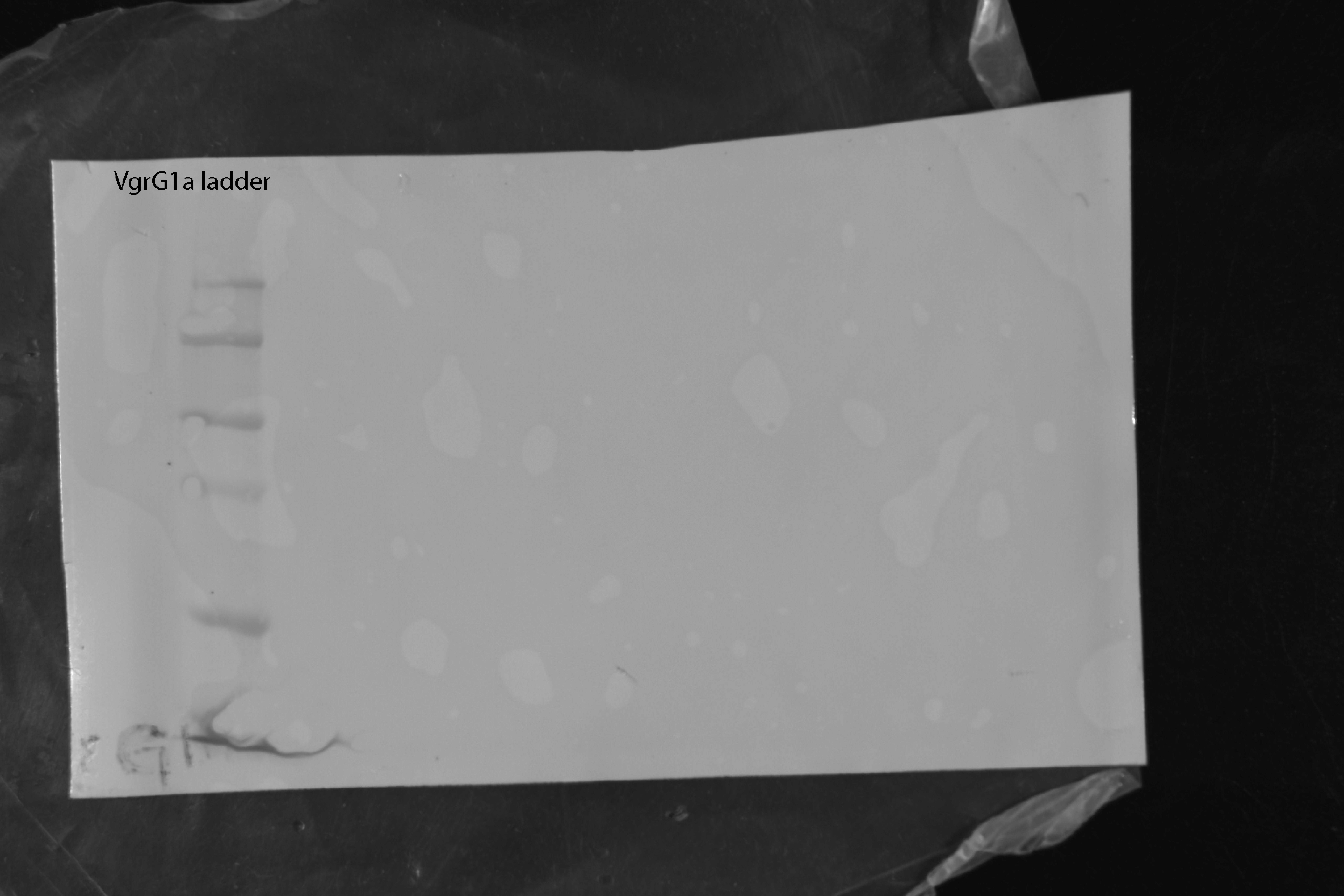
VgrG1a ladder

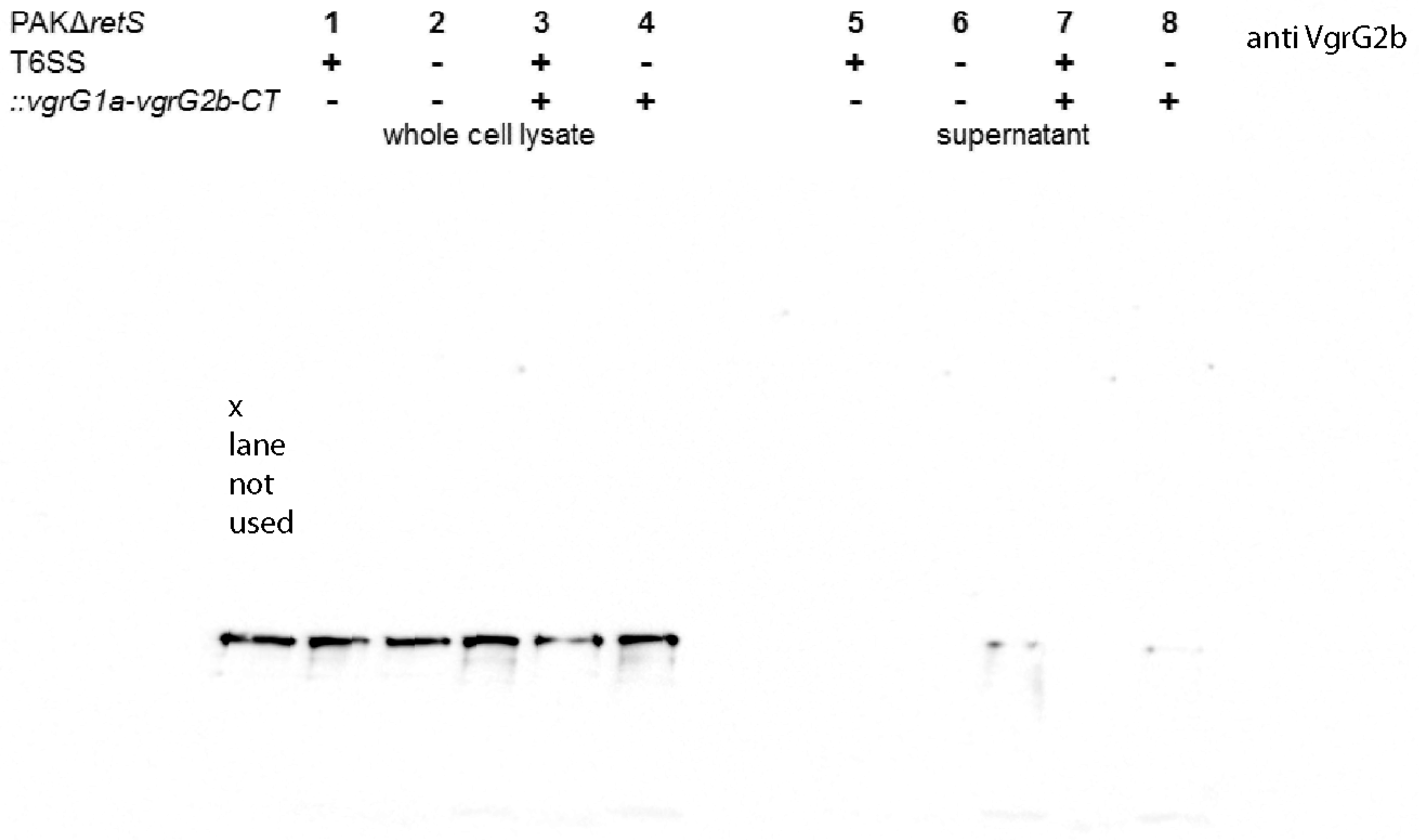
PAKΔretS
T6SS
::vgrG1a-vgrG2b-CT
1 2 3 4 5 6 7 8
+ - + - + - + -
- - + + - - + +
whole cell lysate
supernatant
anti VgrG2b
x
lane
not
used

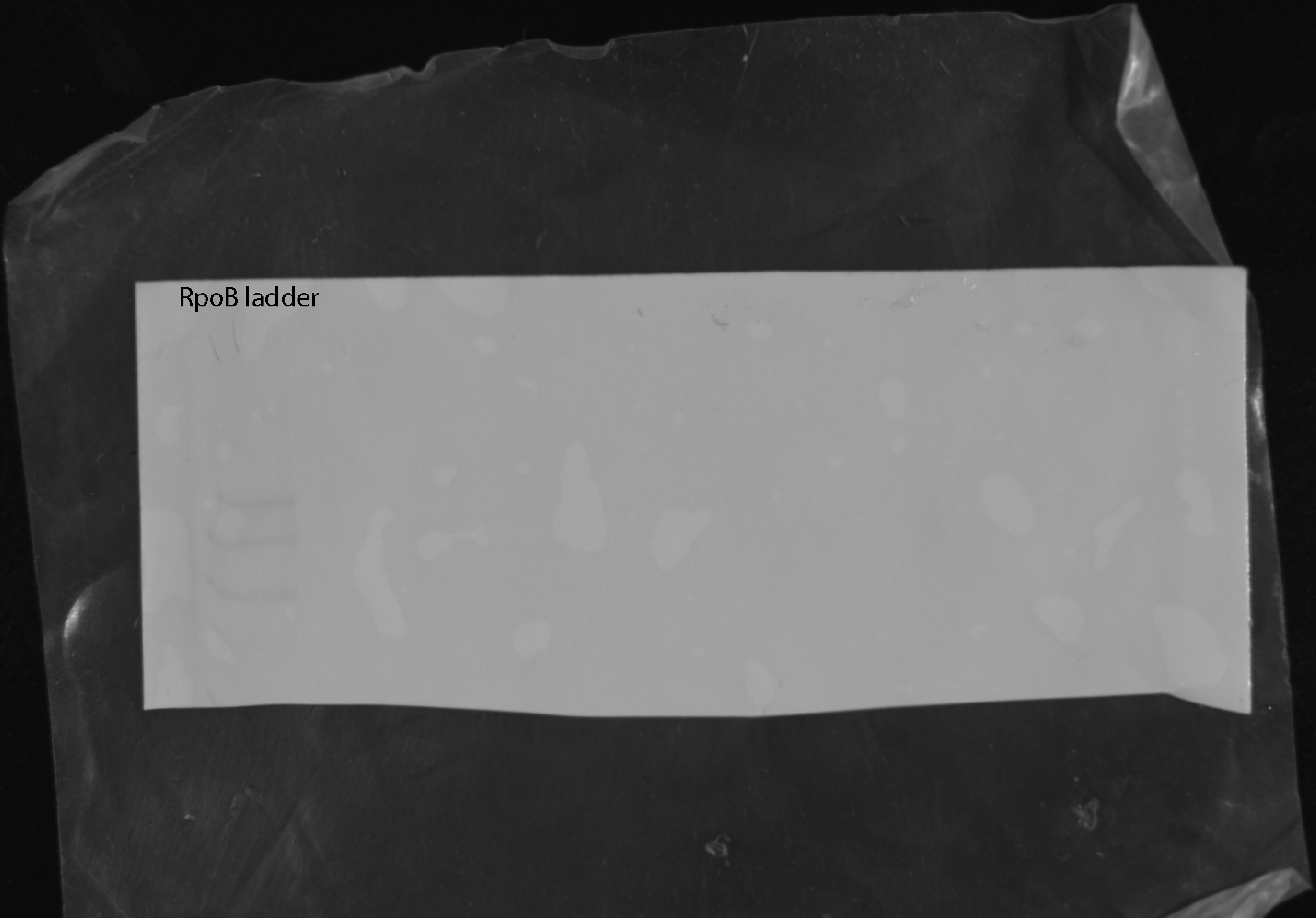
RpoB ladder

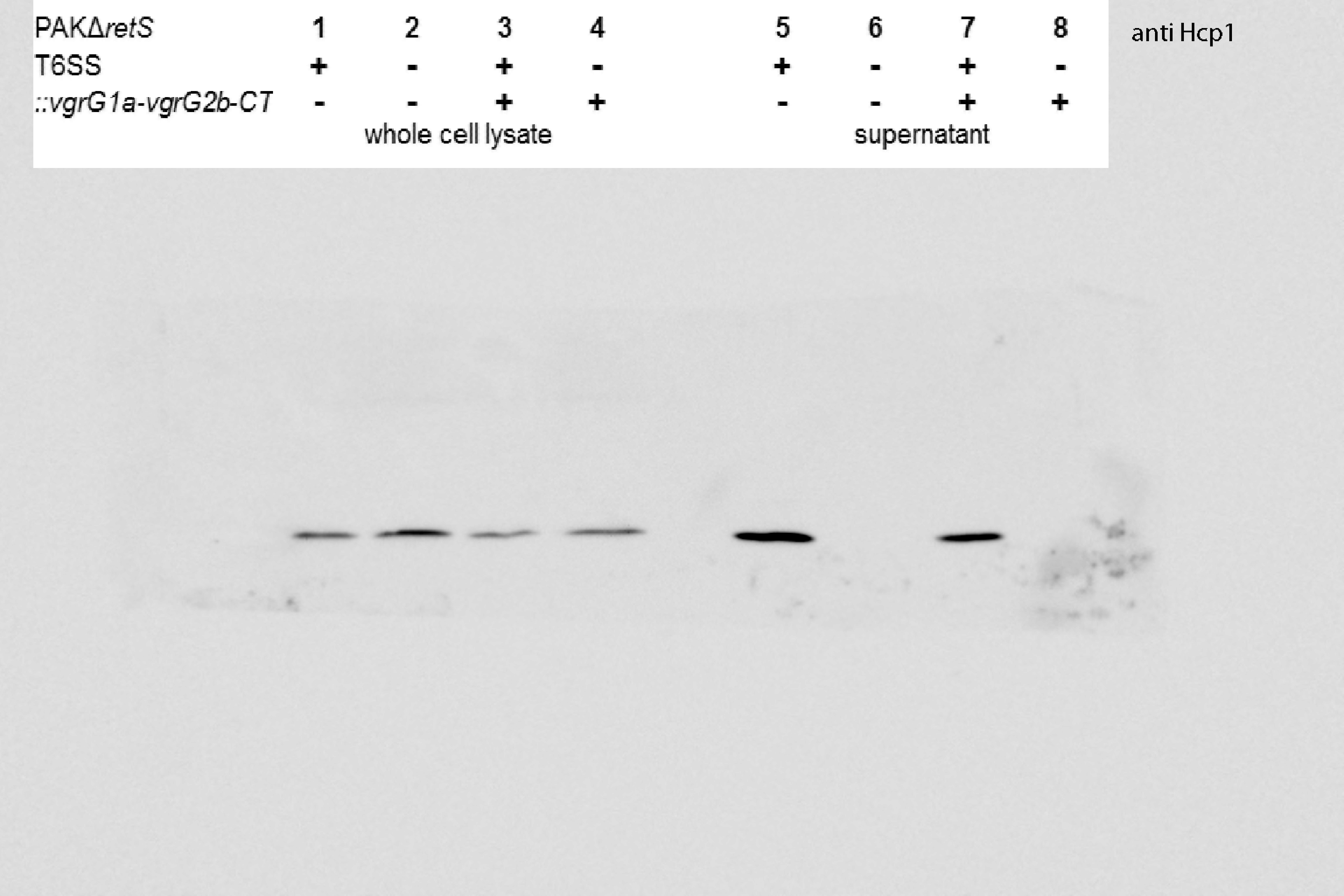
PAKΔretS 1 2 3 4 5 6 7 8
T6SS + - + - + - + -
::vgrG1a-vgrG2b-CT - - + + - - + +
whole cell lysate
supernatant
anti Hcp1

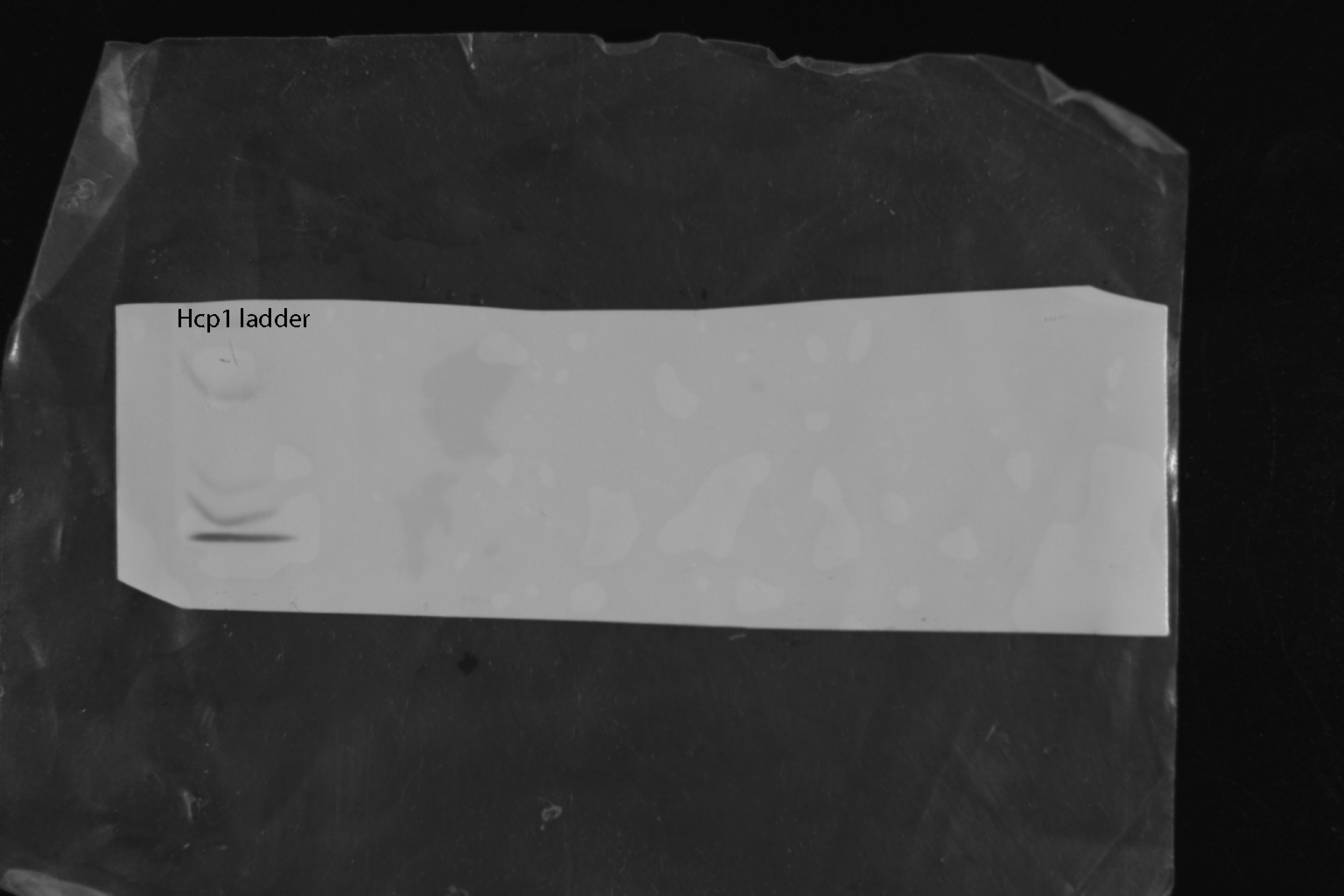
Hcp1 ladder

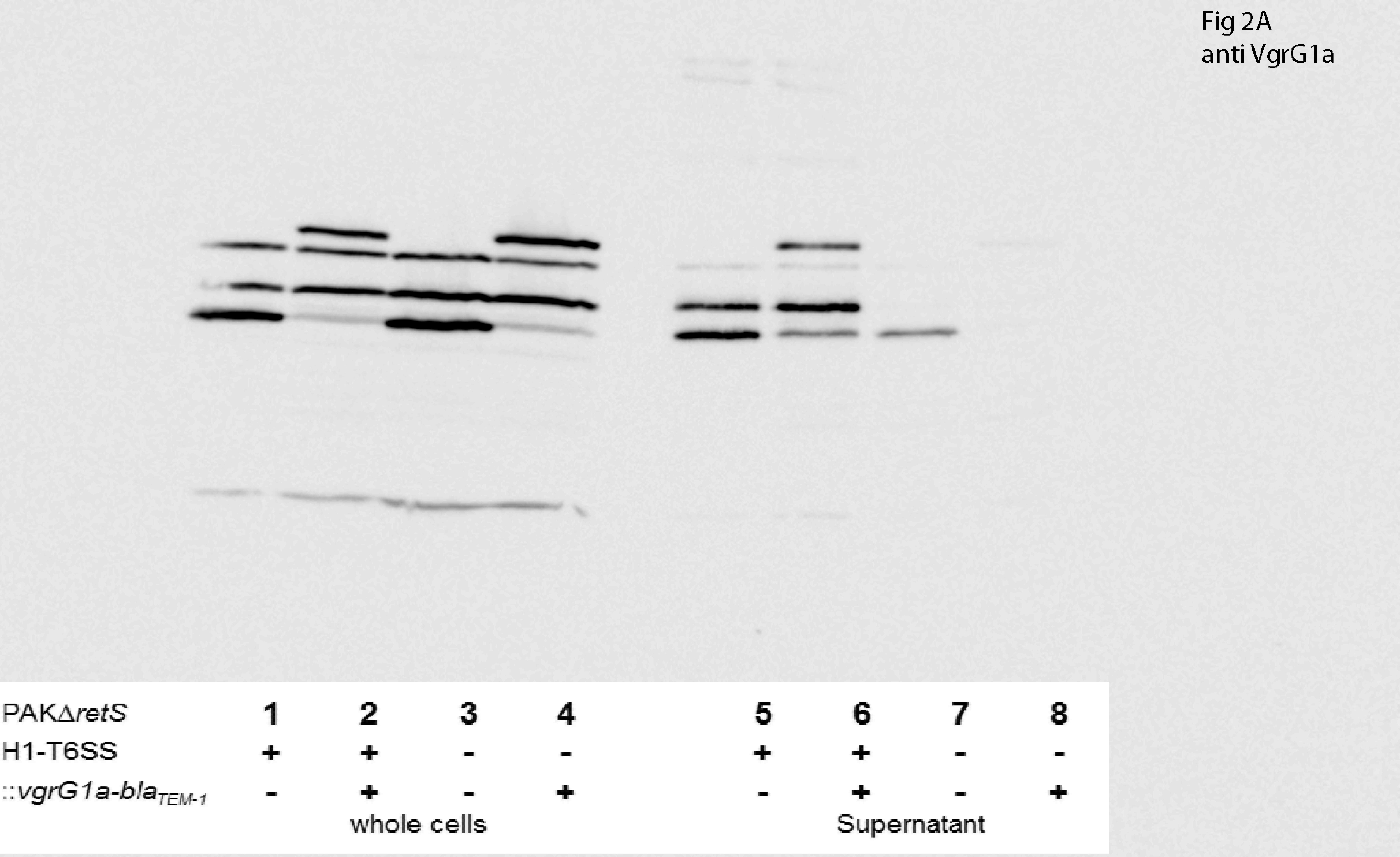
Fig 2A
anti VgrG1a
PAKΔretS 1 2 3 4 5 6 7 8
H1-T6SS + + - - + + - -
::vgrG1a-bla TEM-1 - + - + - + - +
whole cells
Supernatant

Fig 2A
VgrG1a ladder

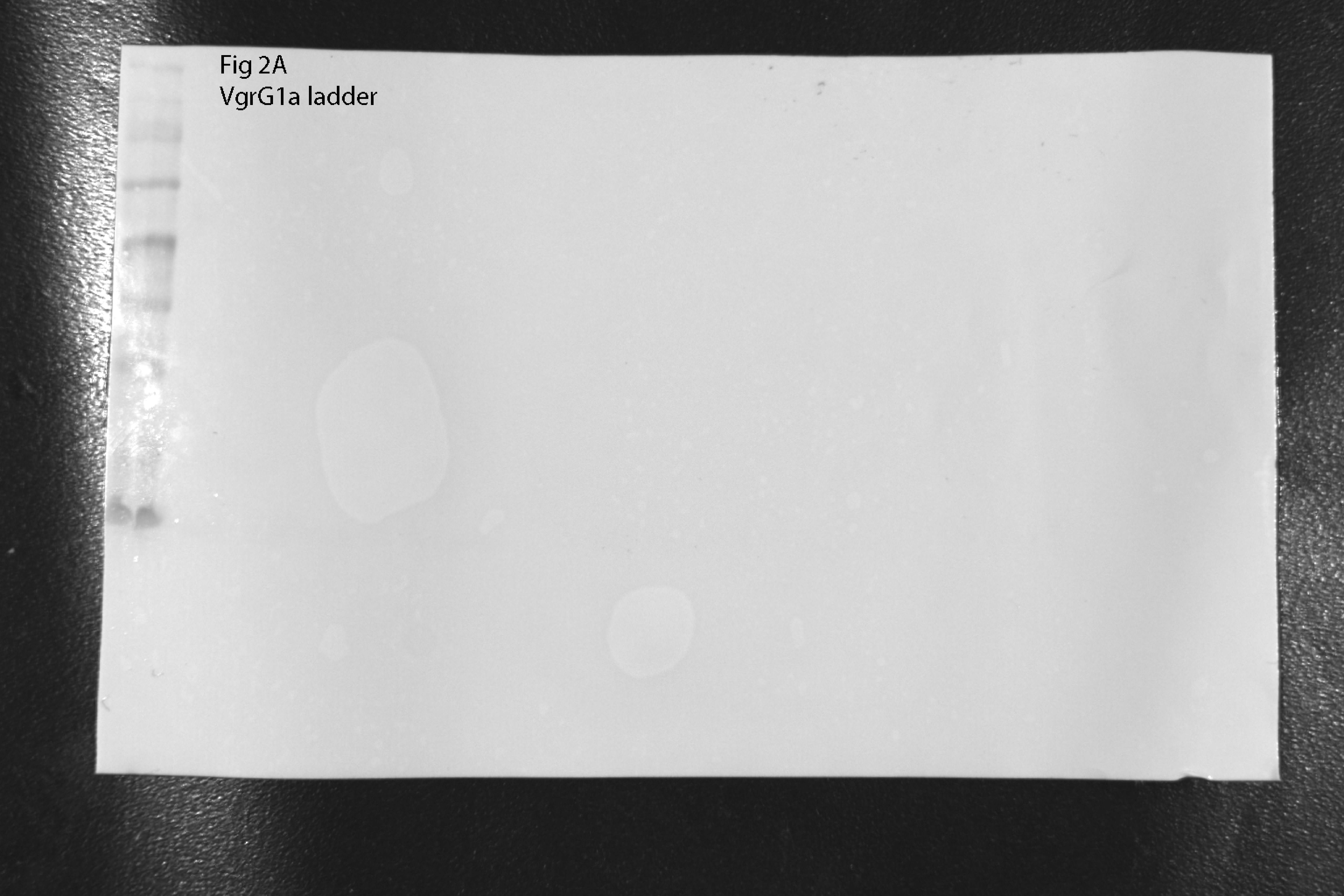

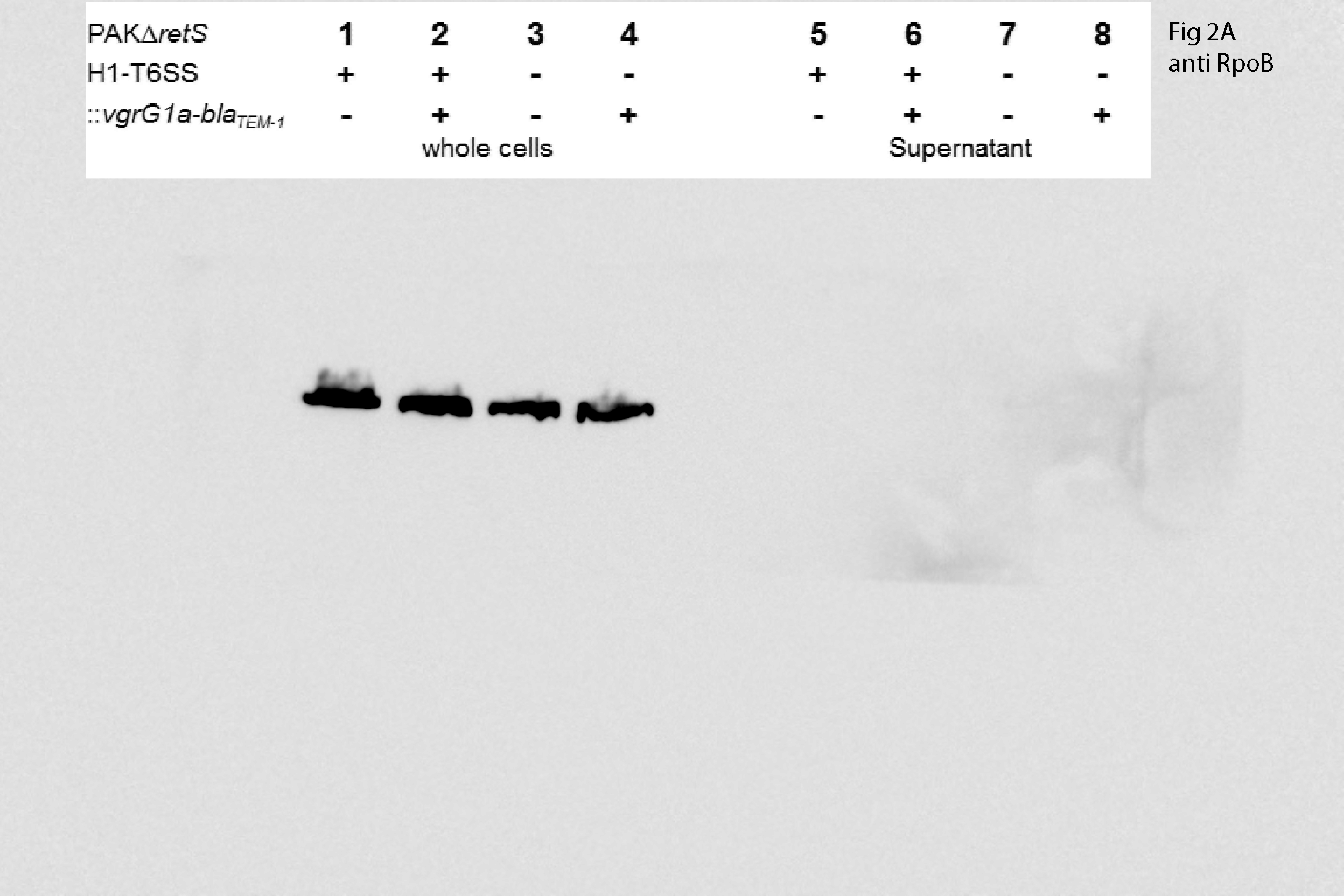
PAKΔretS 1 2 3 4 5 6 7 8
H1-T6SS + + - - + + - -
::vgrG1a-bla$_{TEM-1}$ - + - + - + - +
whole cells
Supernatant
Fig 2A
anti RpoB

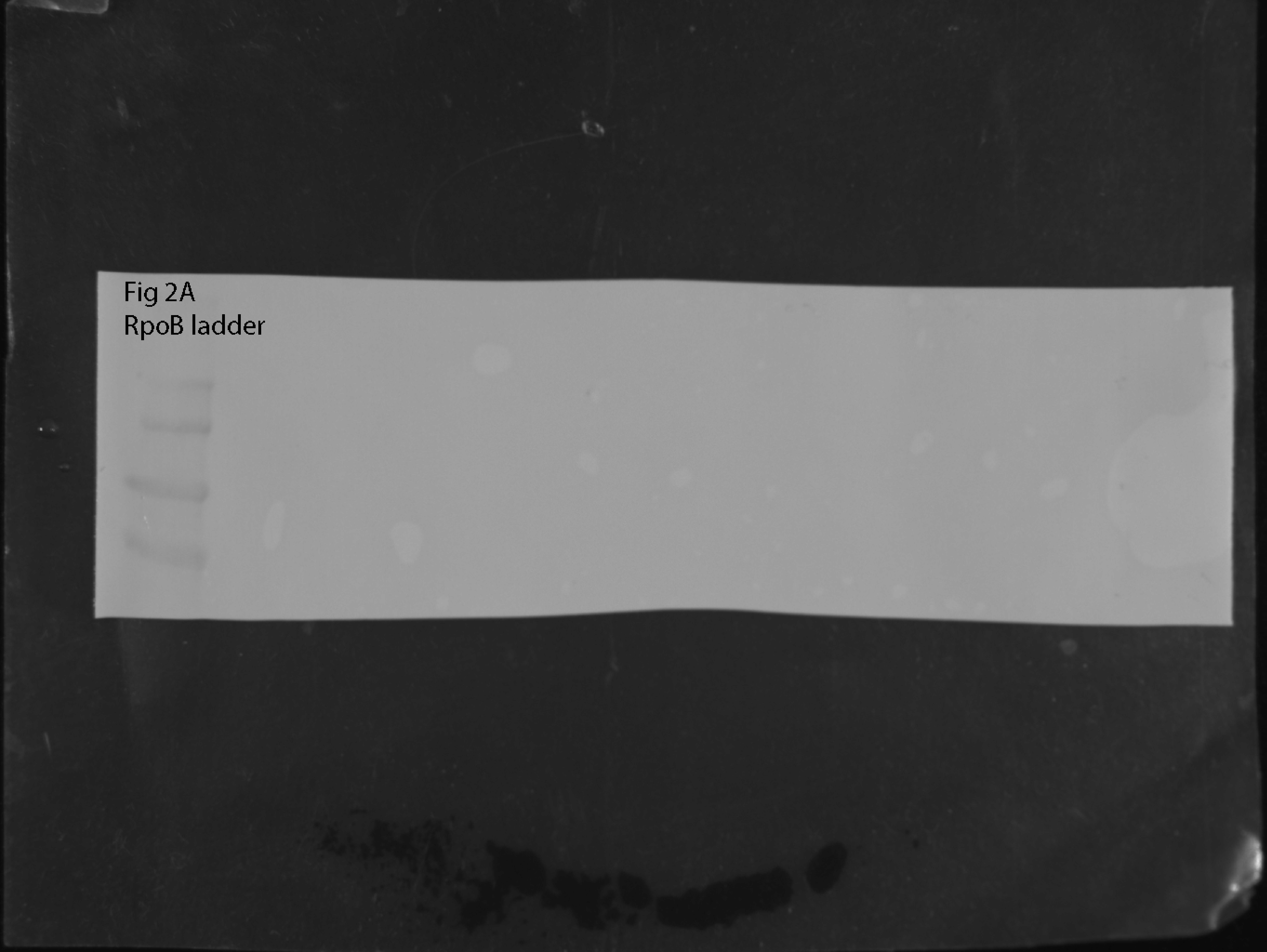
Fig 2A
RpoB ladder

Fig 2A
Hcp1 ladder

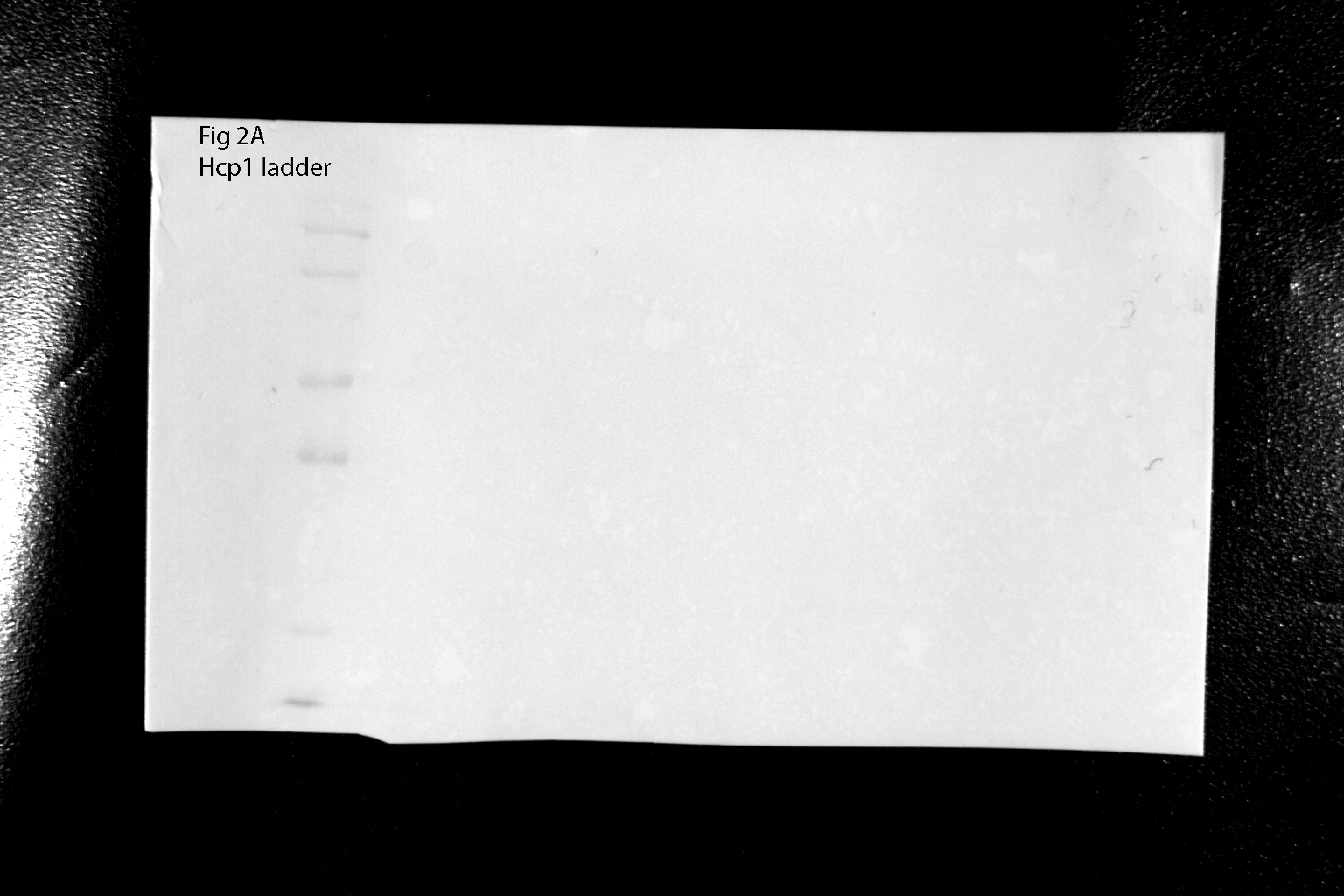

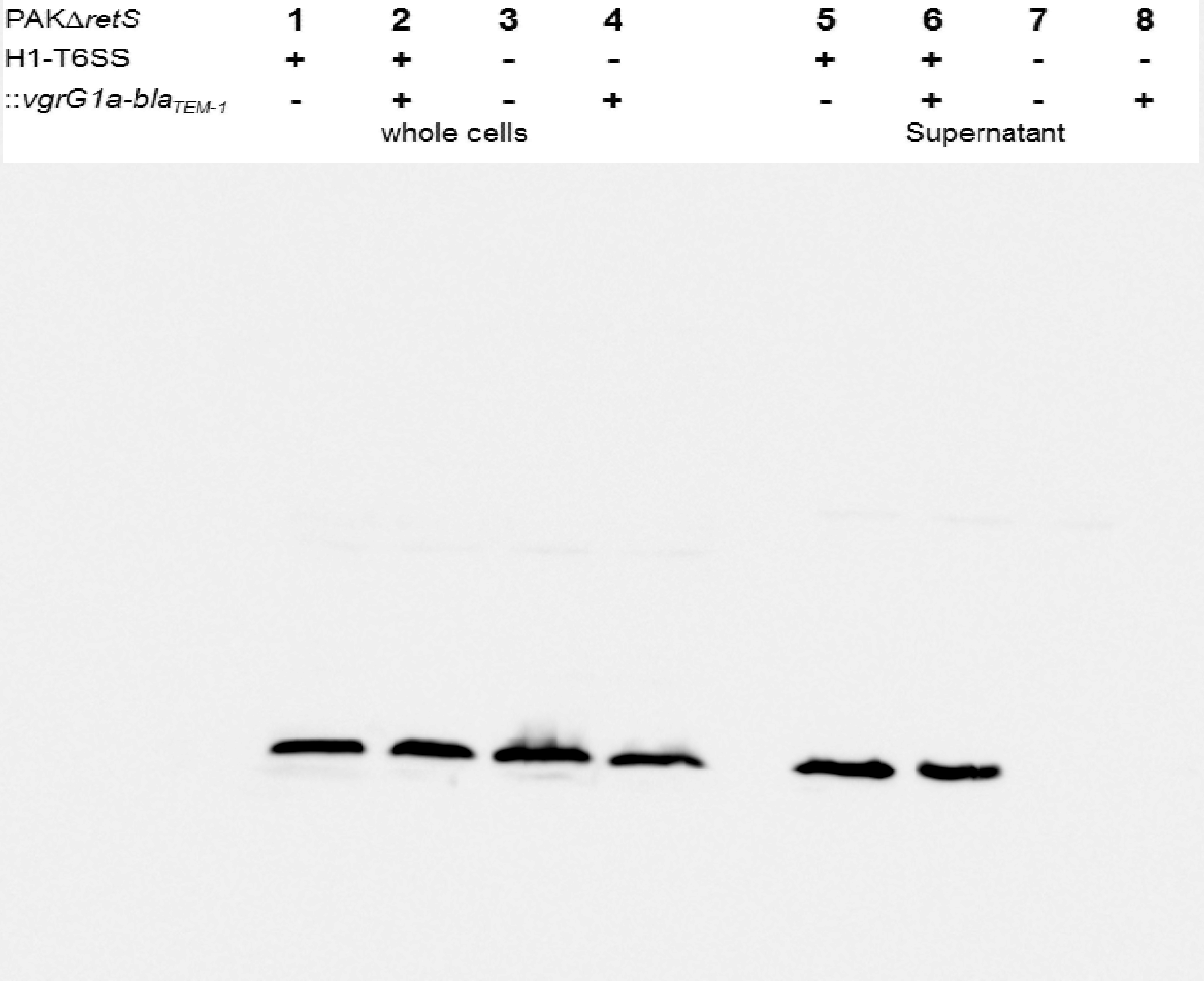


Fig 2A
anti Hcp1

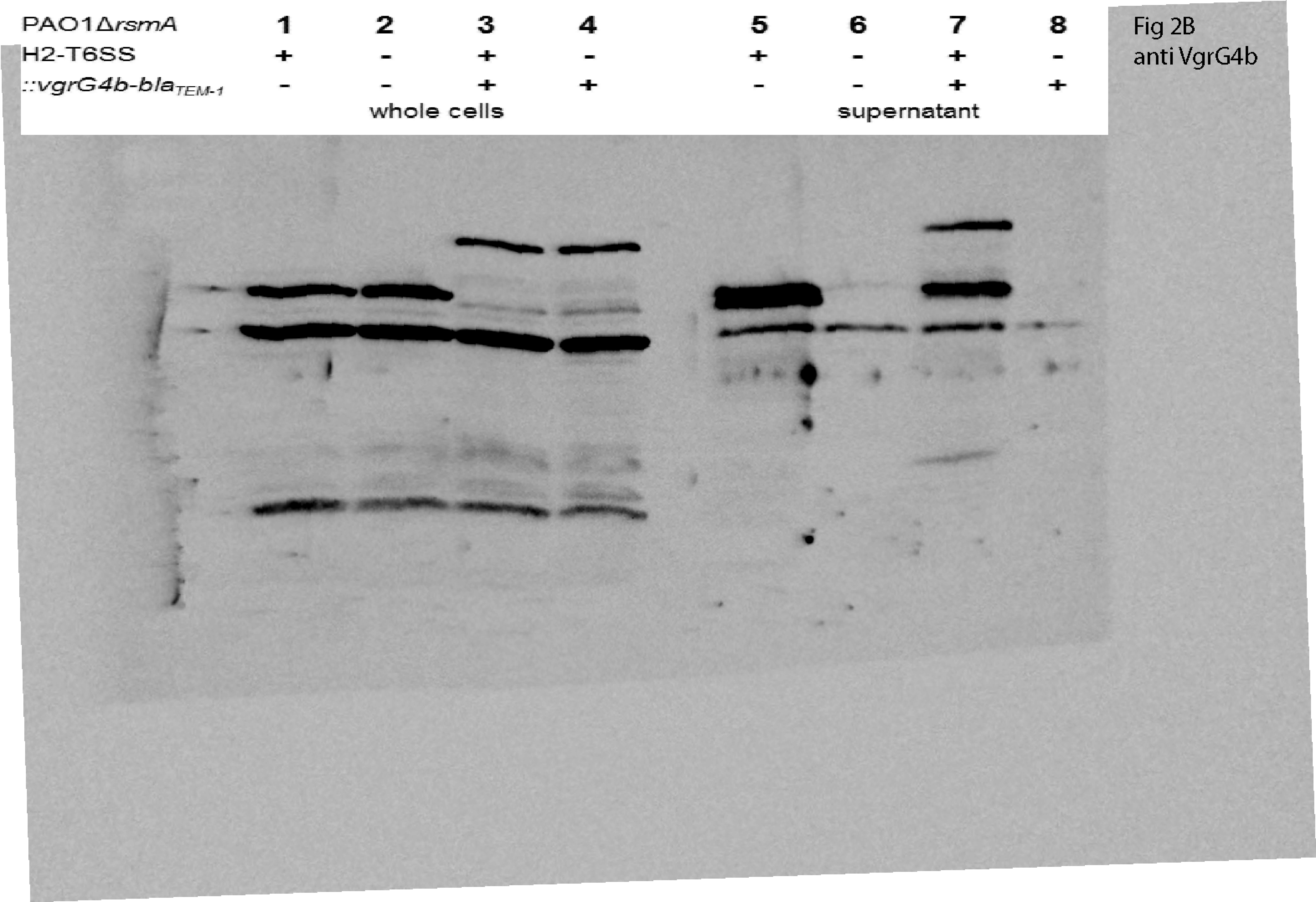
PAO1ΔrsmA
H2-T6SS
::vgrG4b-blaTEM-1
1 2 3 4 5 6 7 8
+ - + - + - + -
- - + + - - + +
whole cells
supernatant
Fig 2B
anti VgrG4b

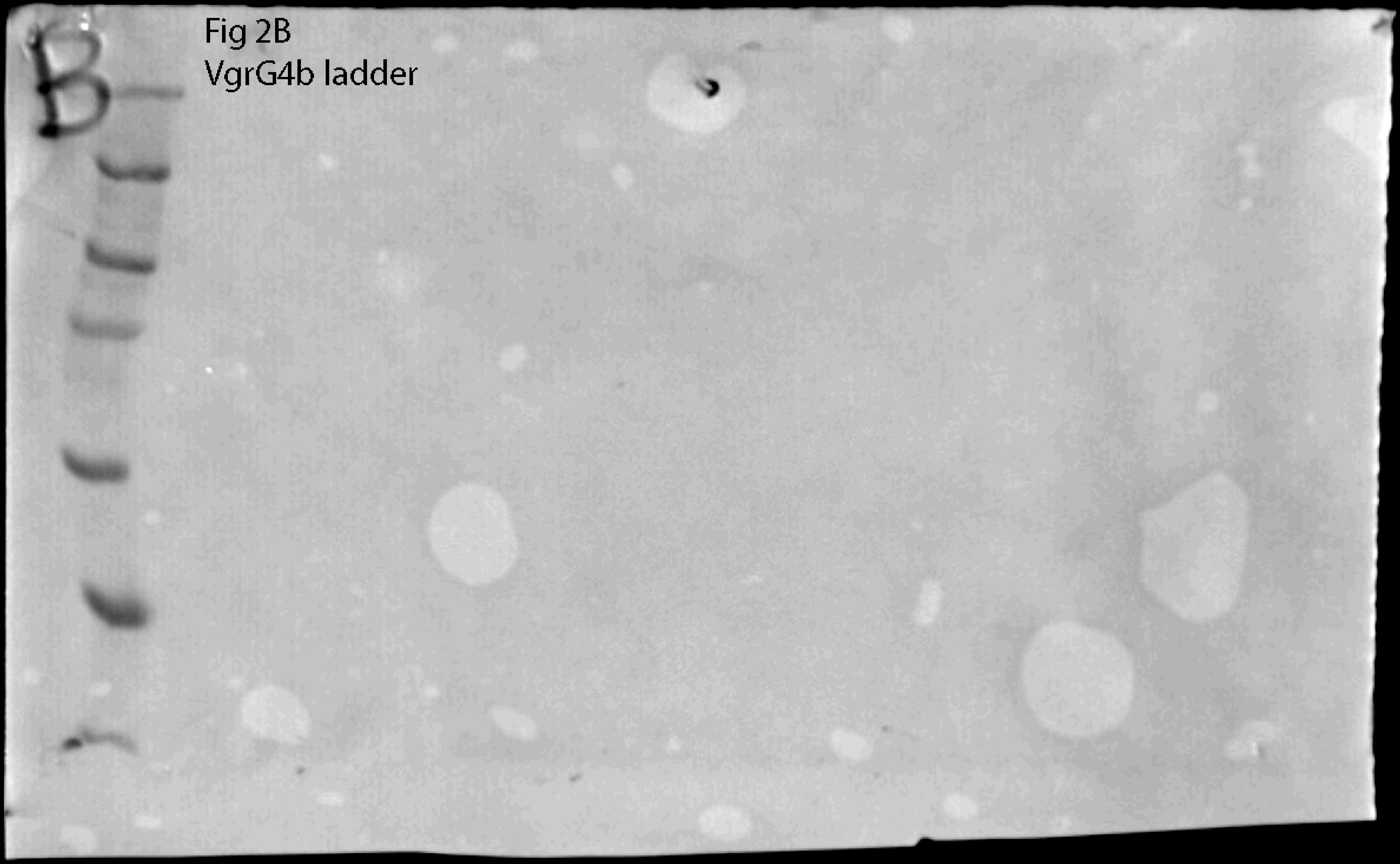
B
Fig 2B
VgrG4b ladder

Fig 2B
anti RpoB

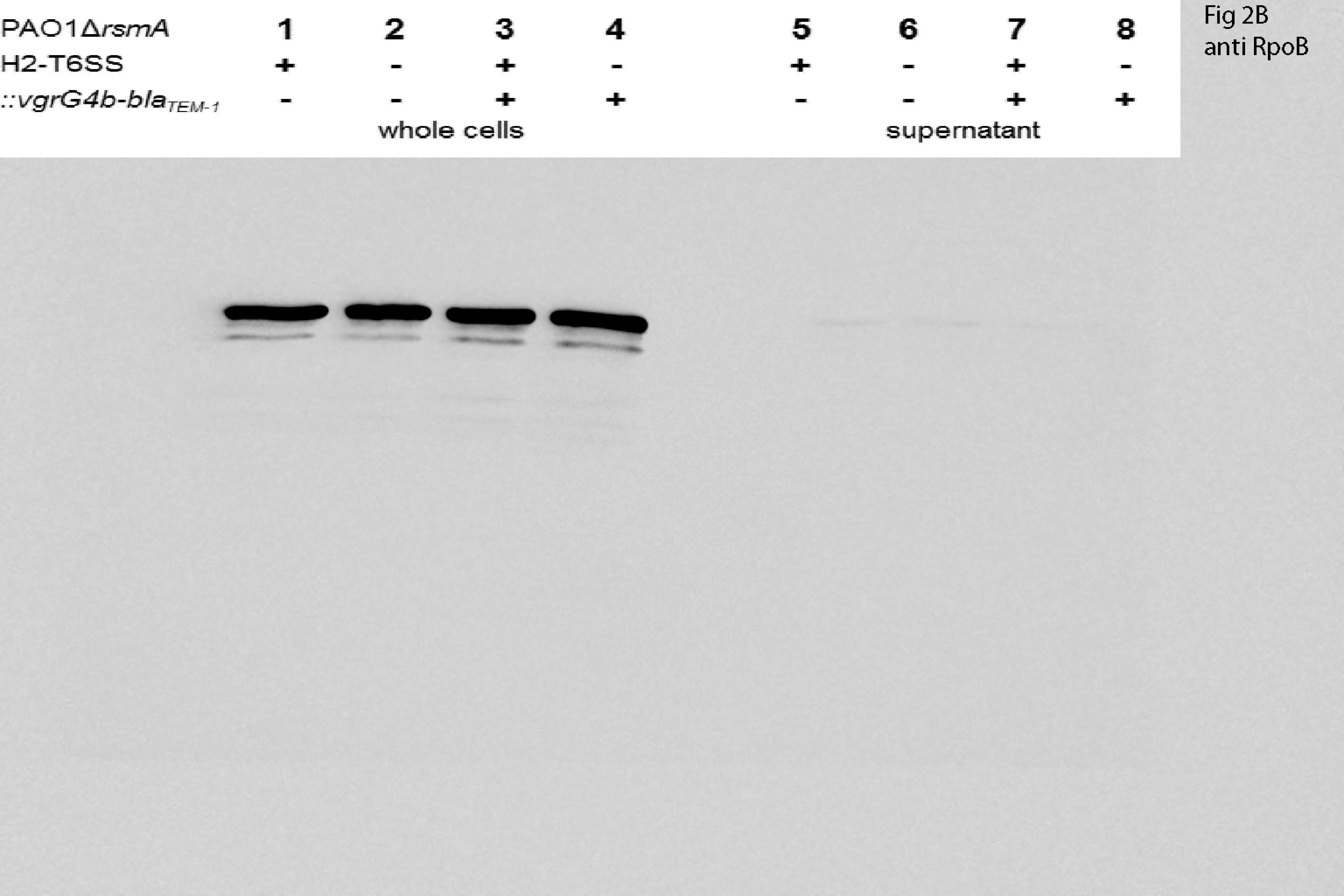

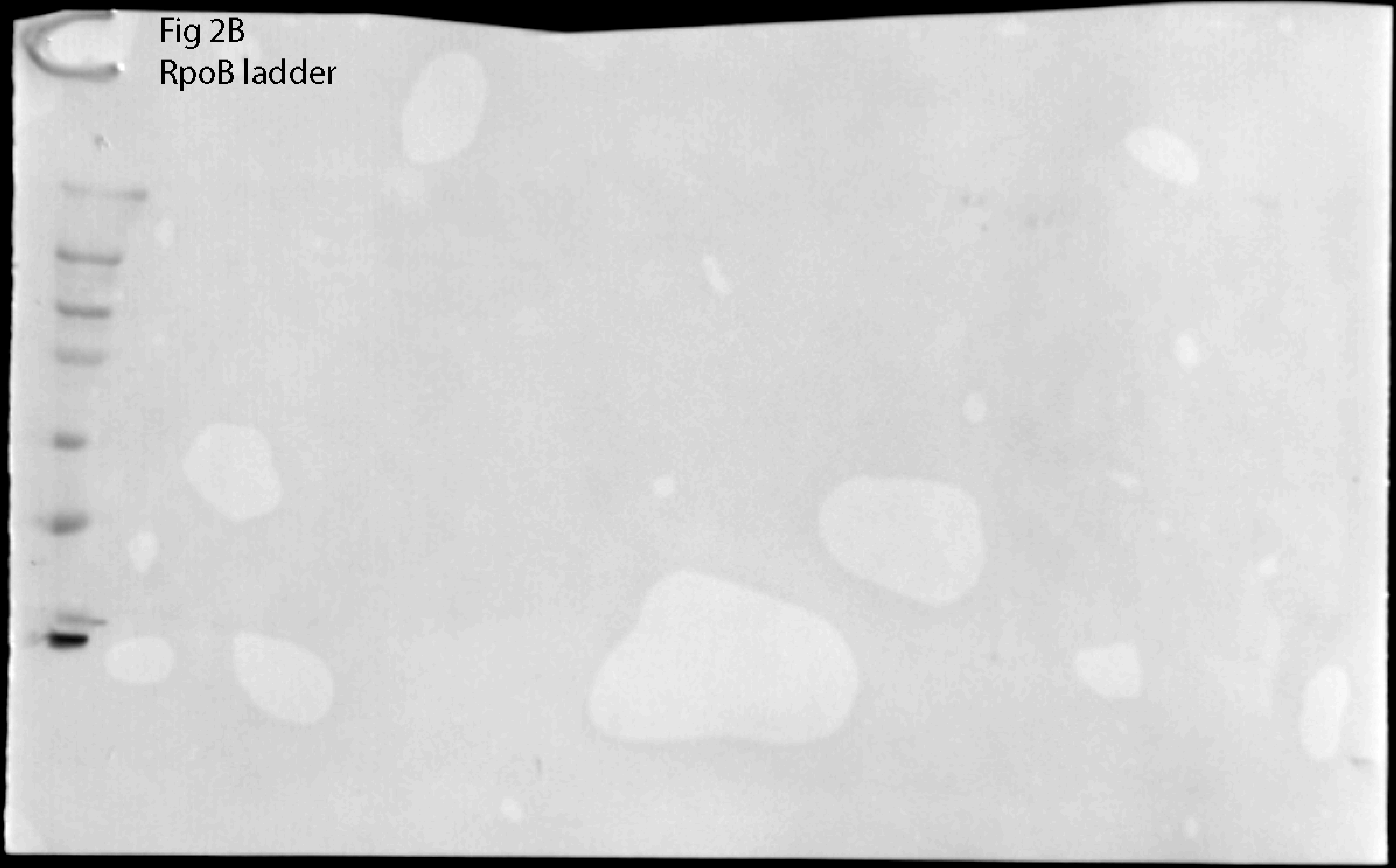
Fig 2B
RpoB ladder

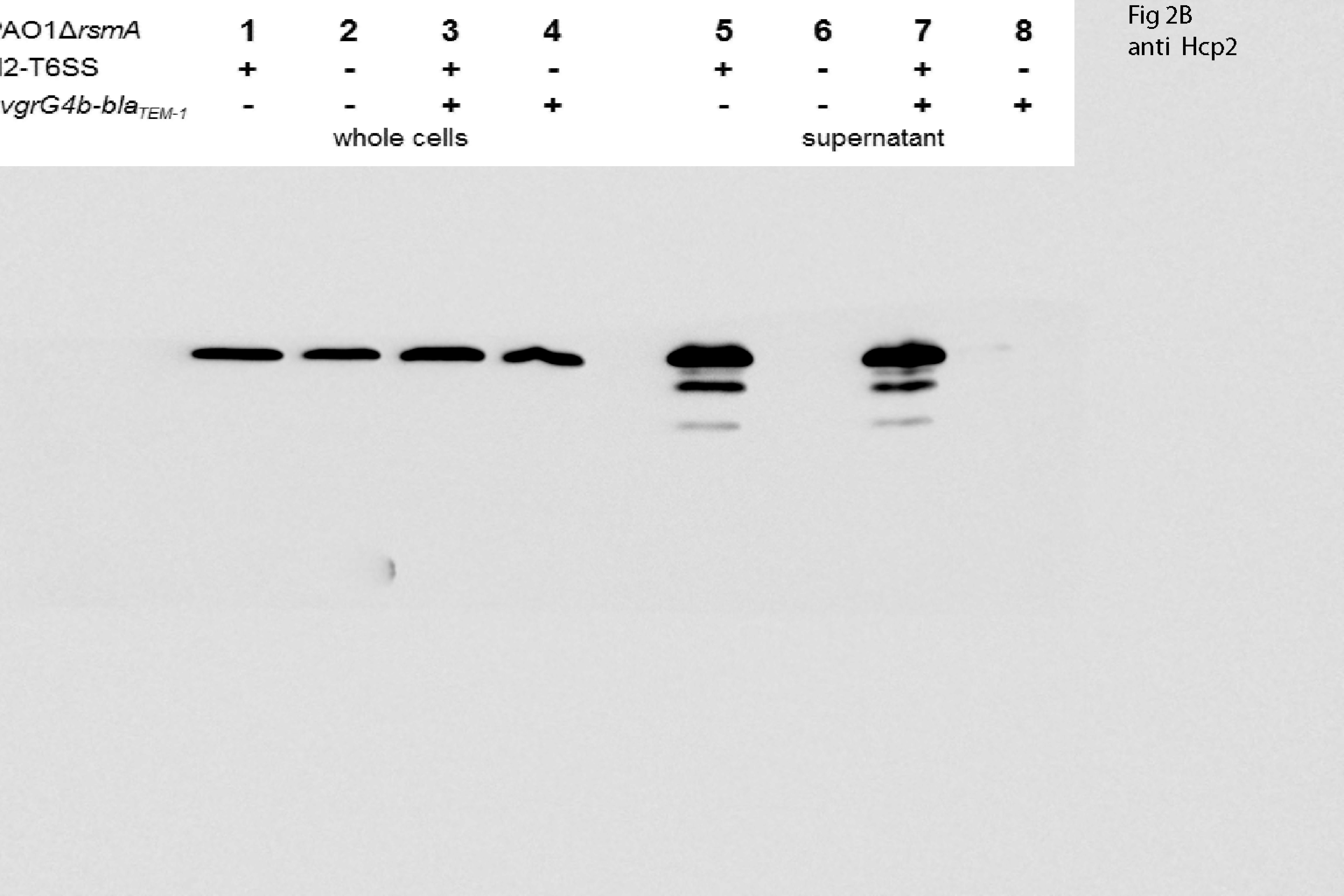


Fig 2B
anti Hcp2

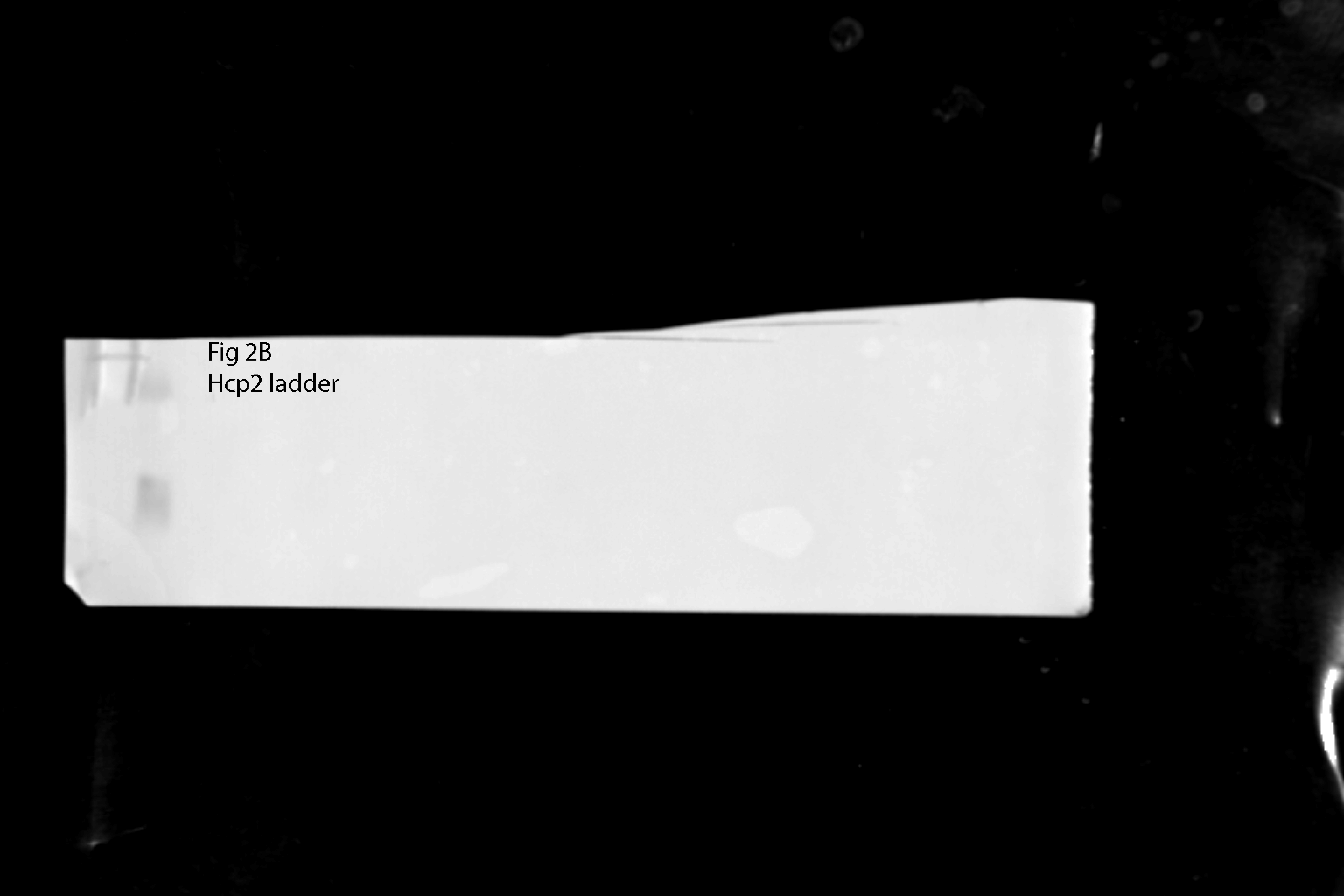

Fig 2B
Hcp2 ladder

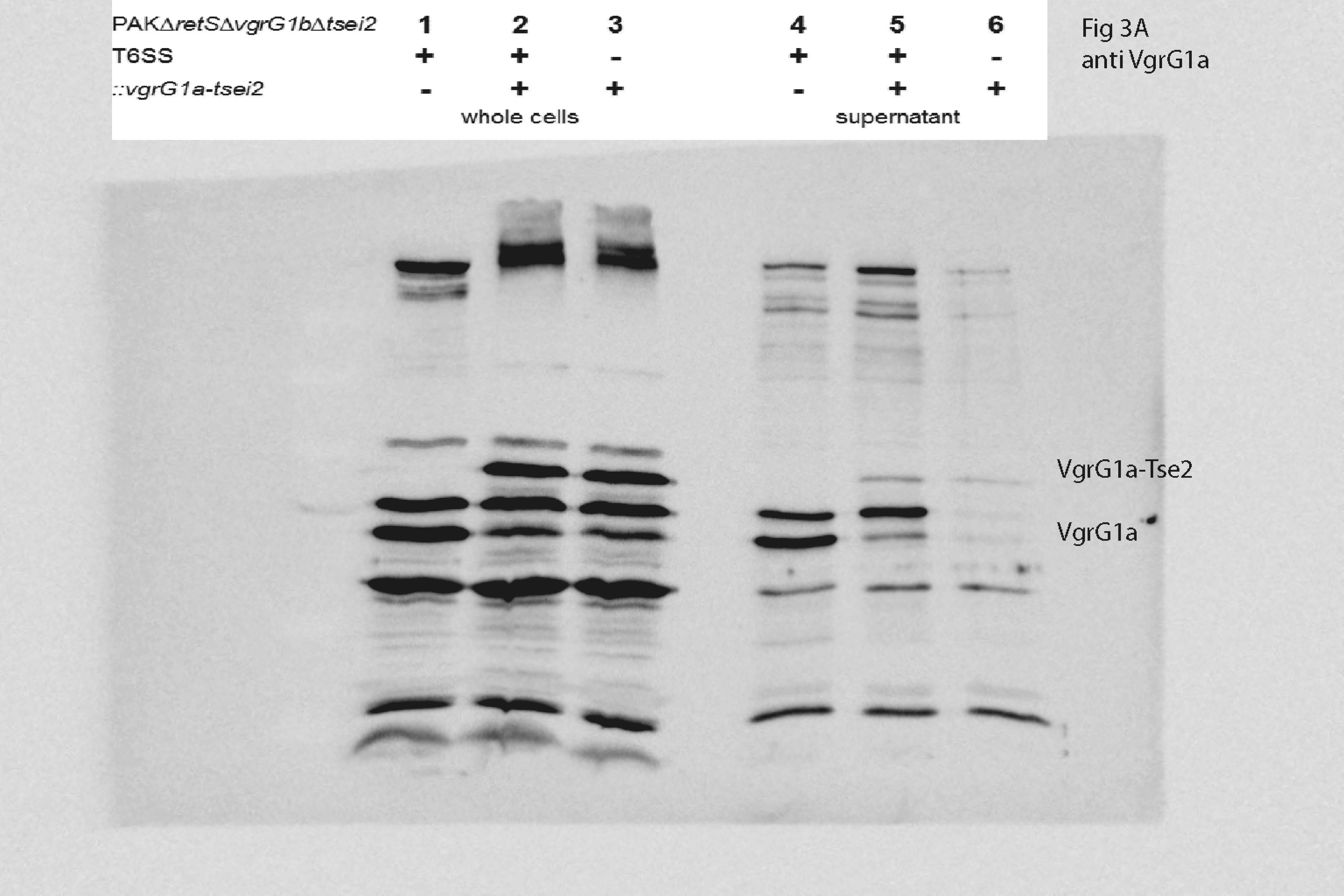
PAKΔretSΔvgrG1bΔtsei2
T6SS
::vgrG1a-tsei2
1 2 3 4 5 6
+ + - + + -
- + + - + +
whole cells
supernatant
Fig 3A
anti VgrG1a
VgrG1a-Tse2
VgrG1a

Fig 3A
VgrG1a ladder

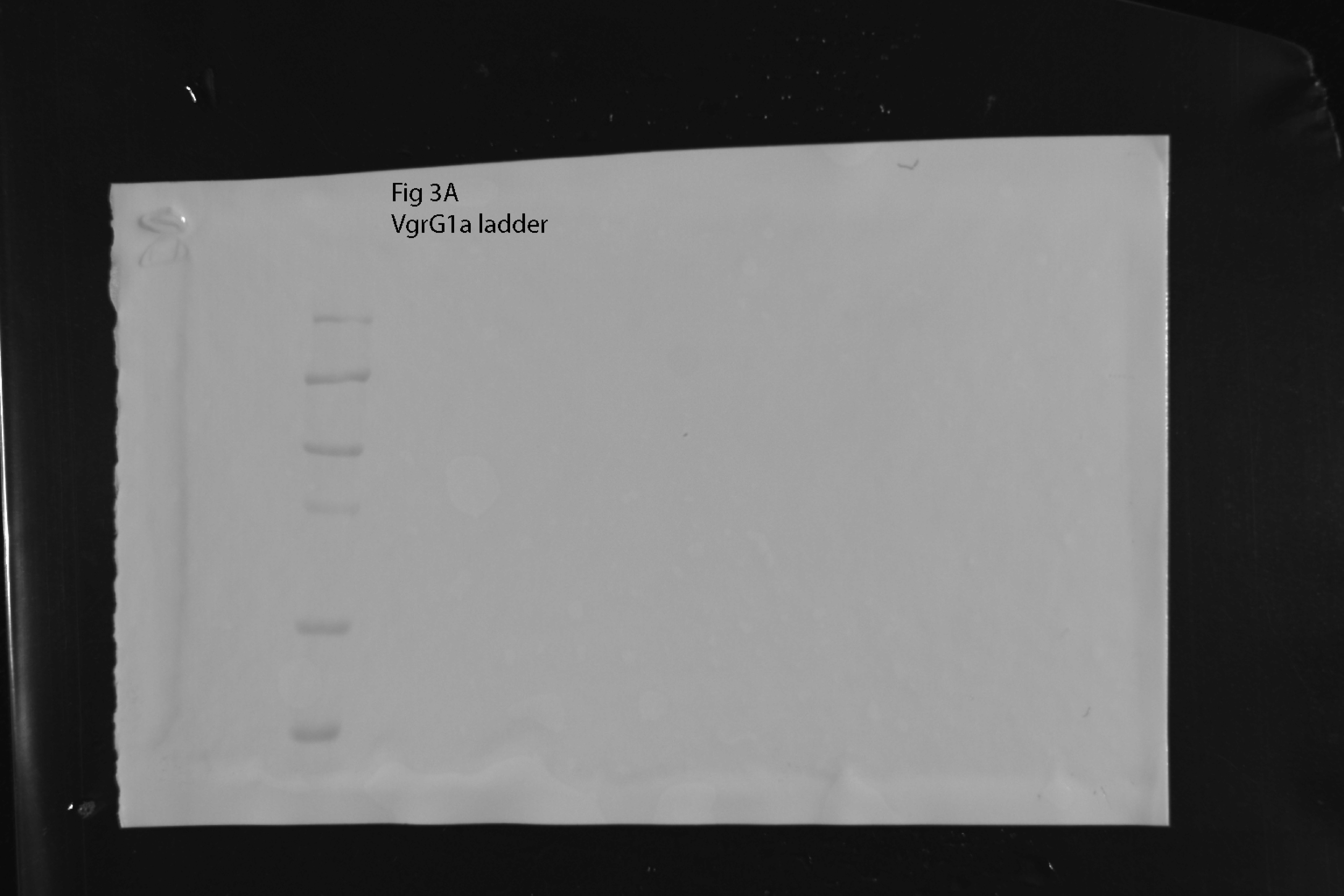

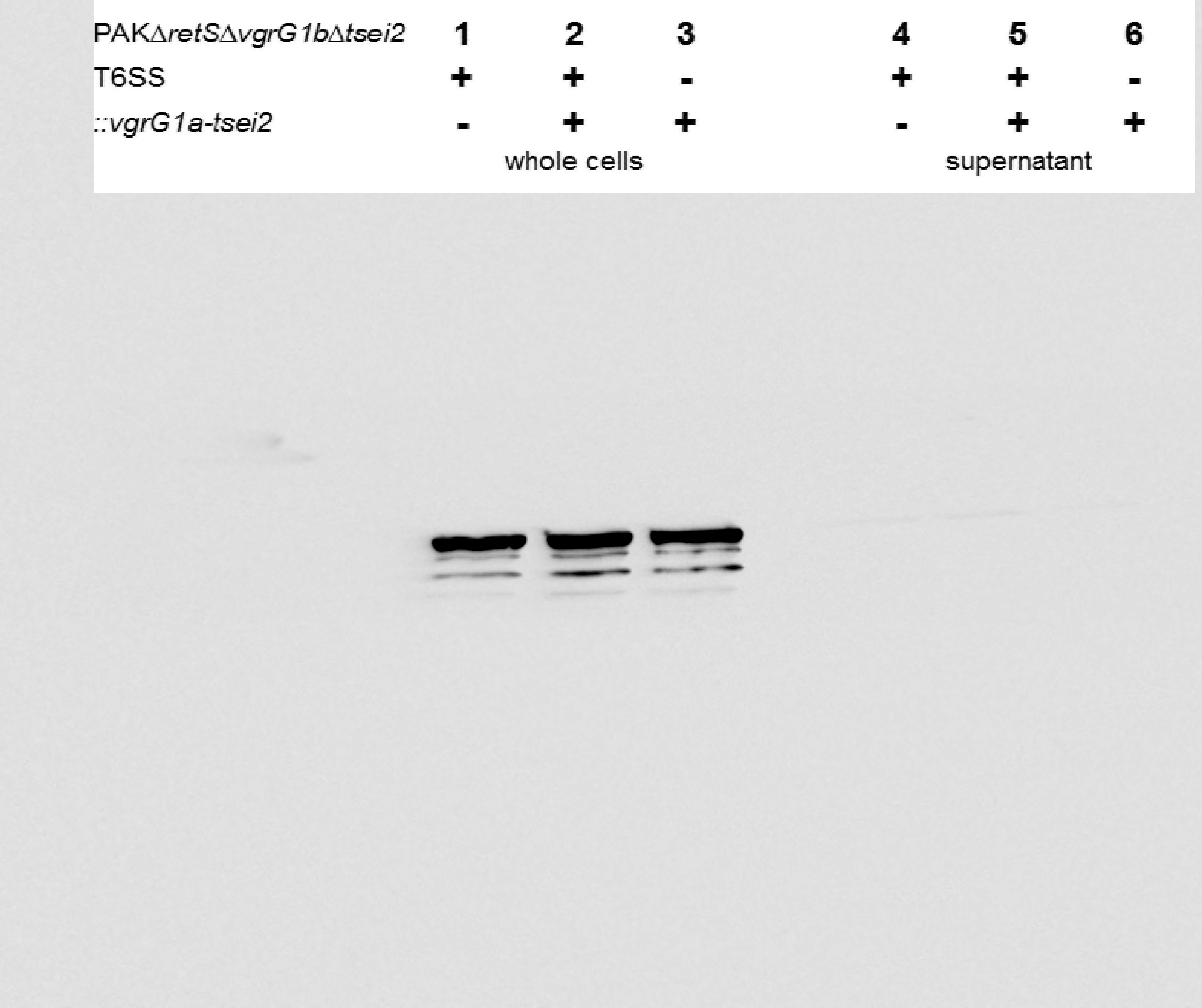


Fig 3A
anti RpoB

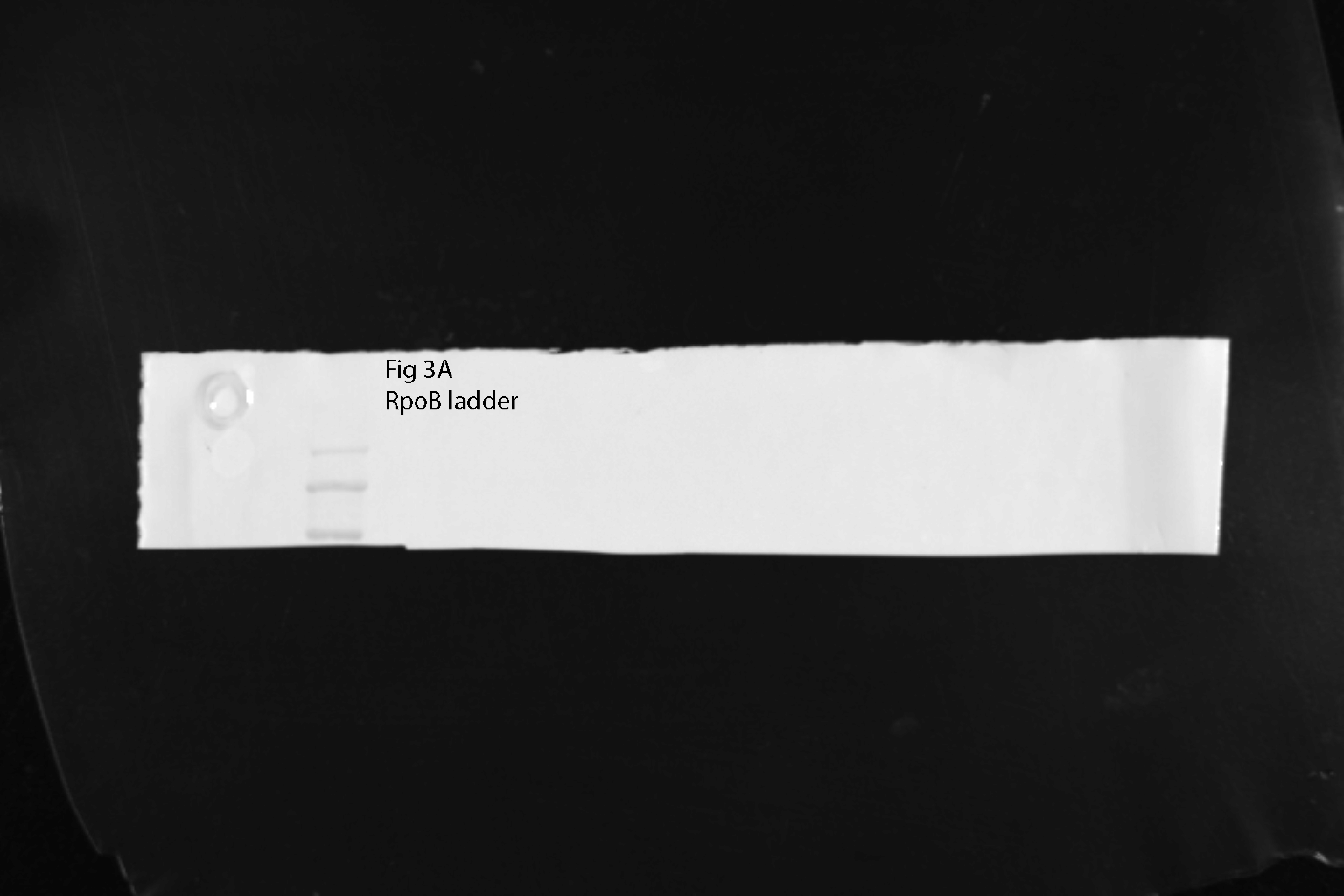
Fig 3A
RpoB ladder

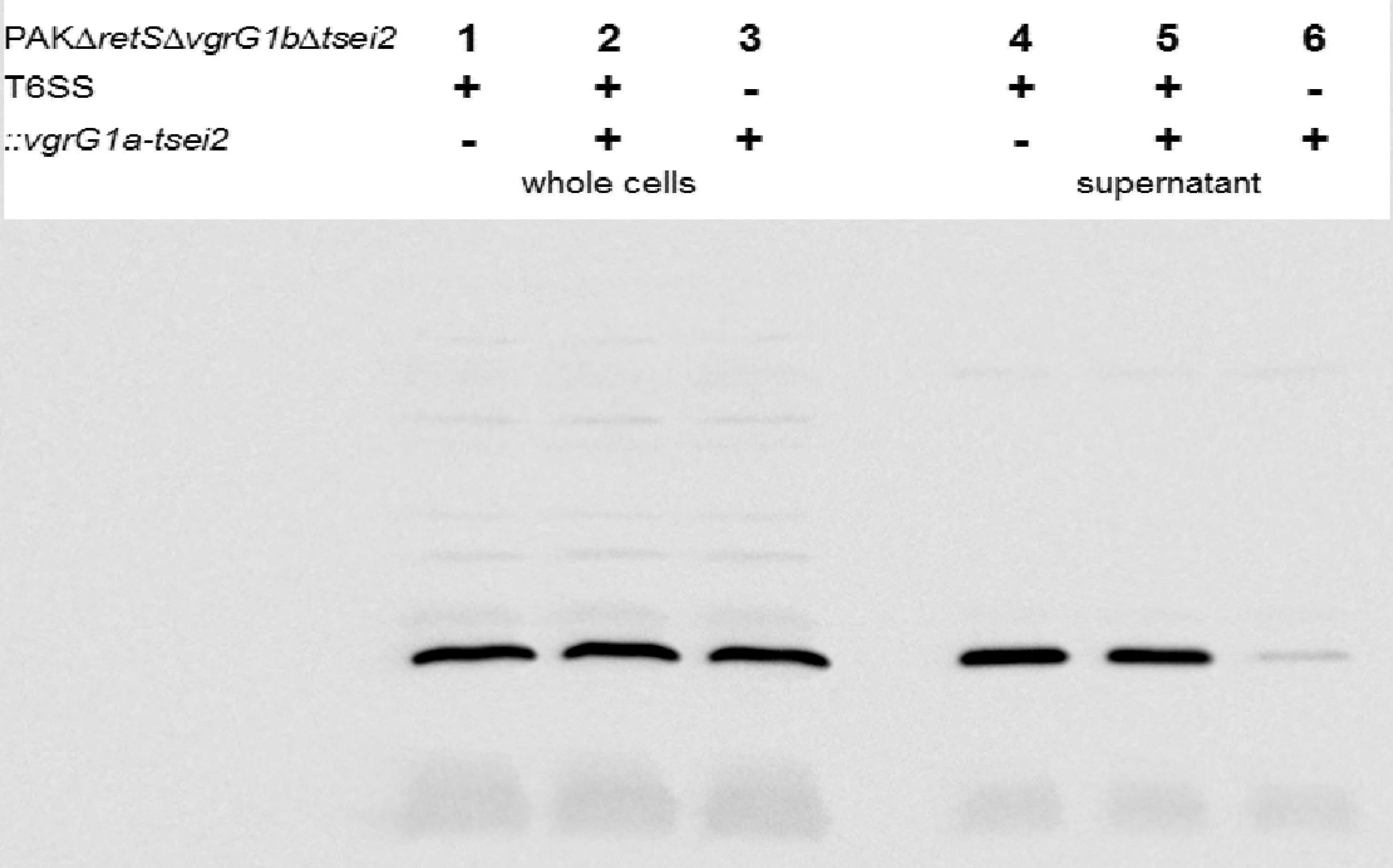

Fig 3A
anti Hcp1

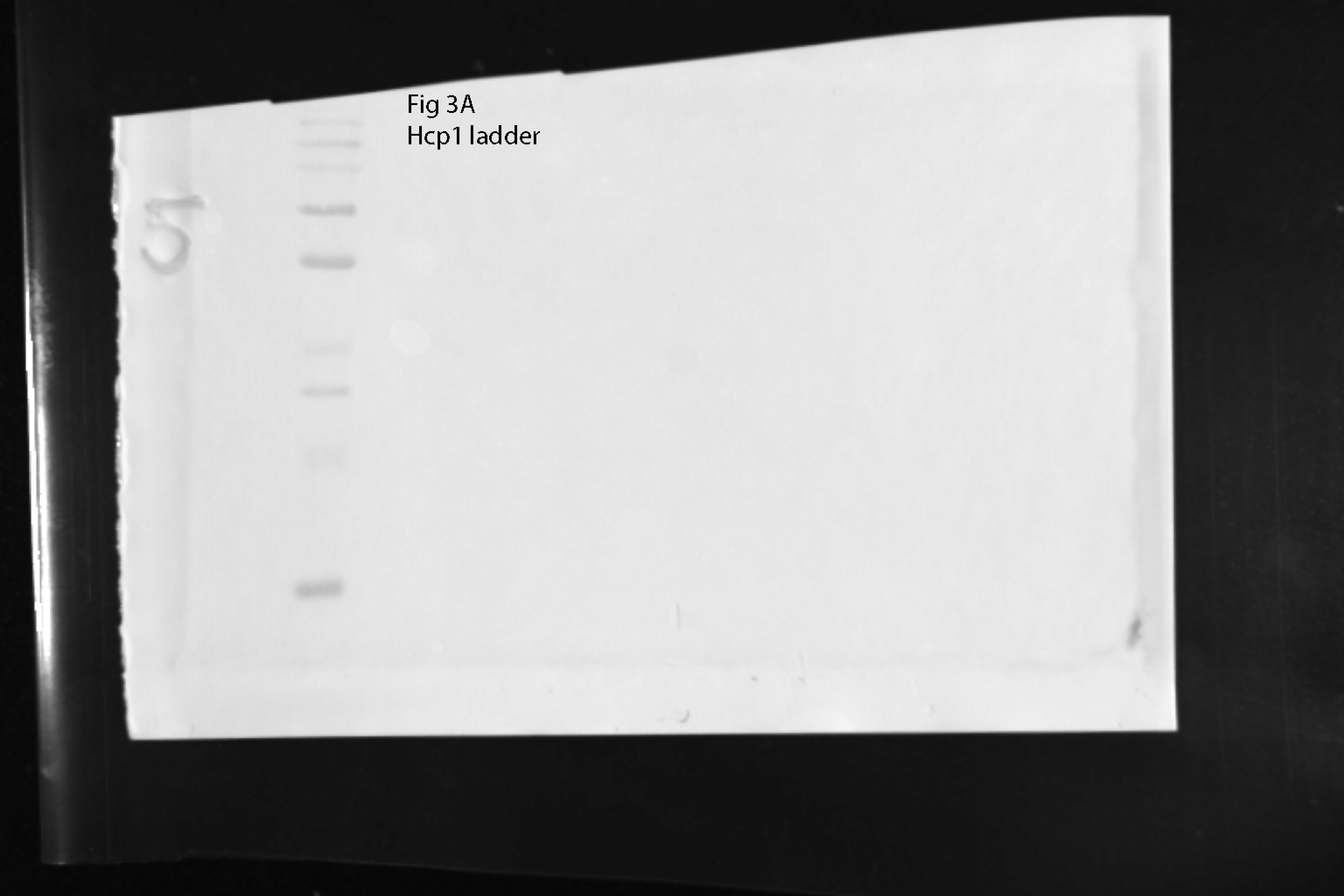
Fig 3A
Hcp1 ladder

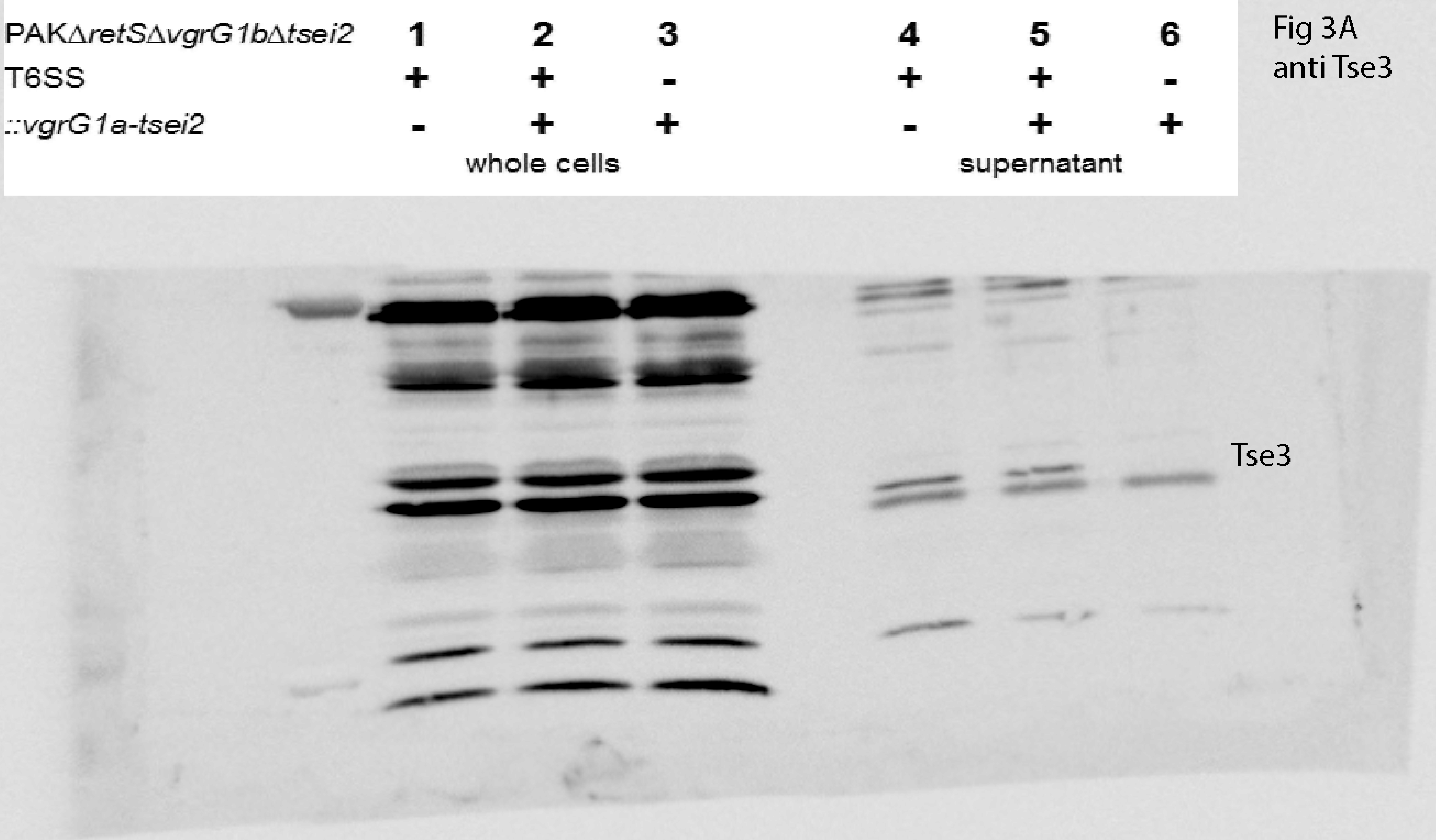
PAKΔretSΔvgrG1bΔtsei2
1
2
3
4
5
6
T6SS
+
+
-
+
+
-
::vgrG1a-tsei2
-
+
+
-
+
+
whole cells
supernatant
Fig 3A
anti Tse3
Tse3

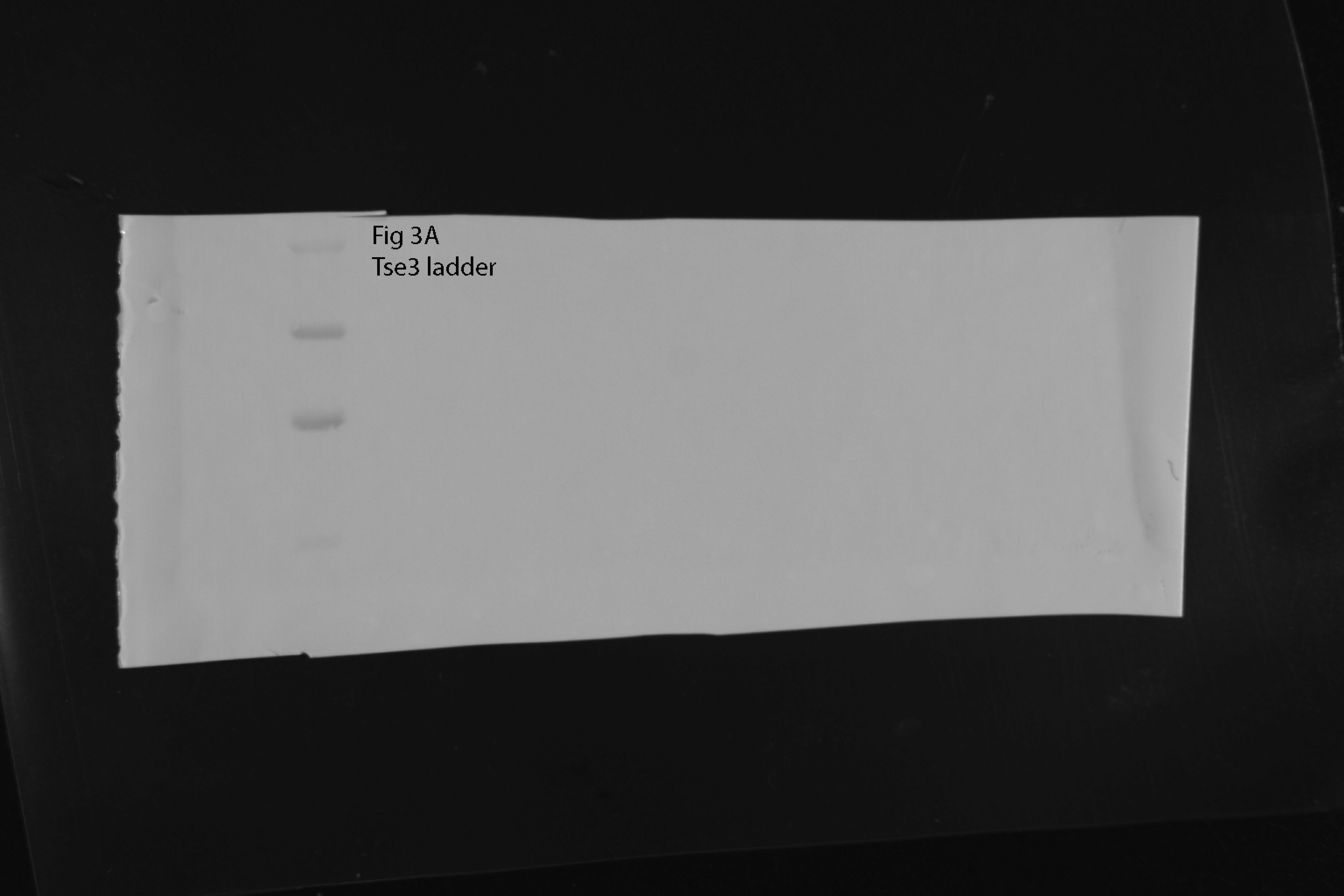
Fig 3A
Tse3 ladder

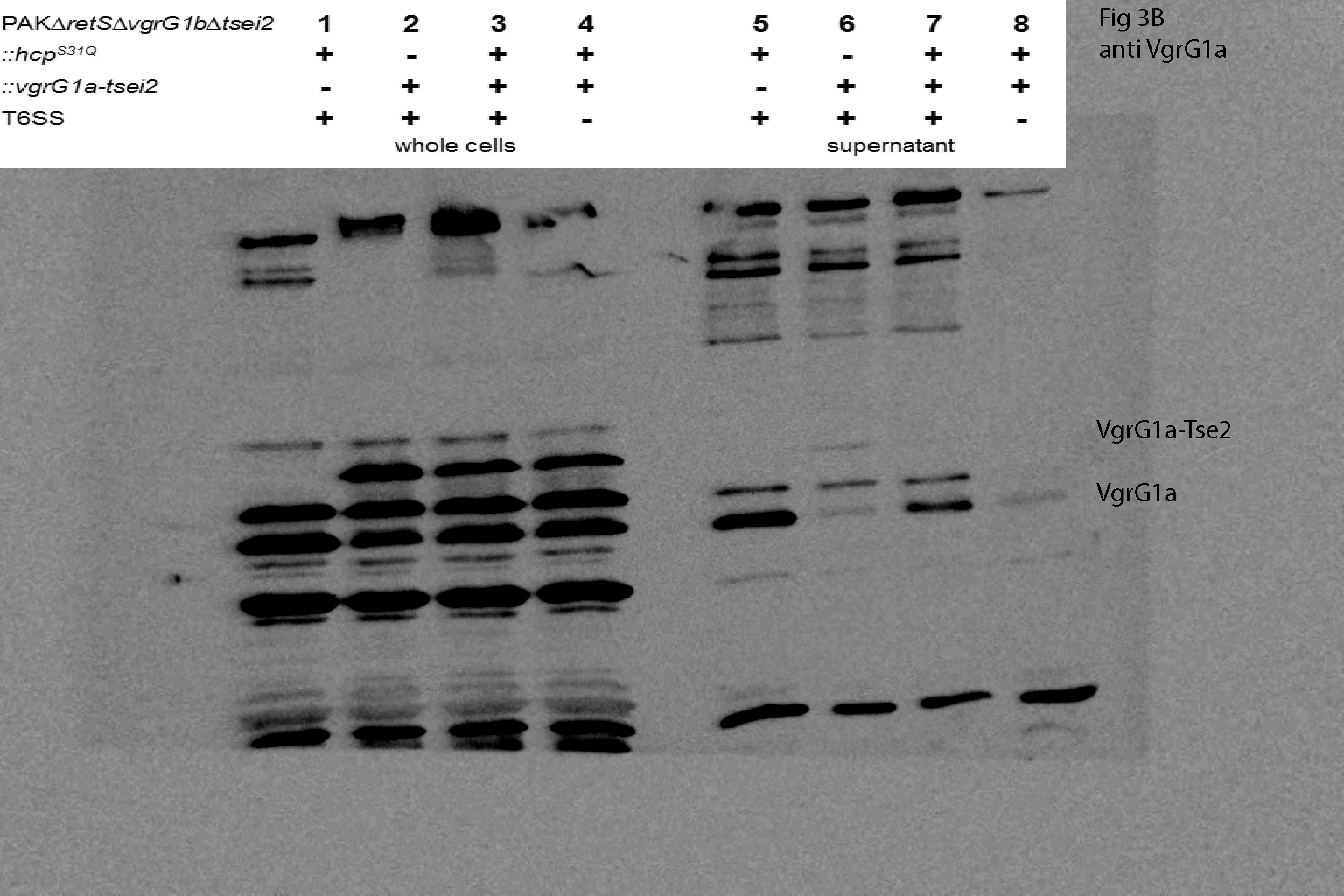
Fig 3B
anti VgrG1a
PAKΔretSΔvgrG1bΔtsei2
::hcp^S31Q
::vgrG1a-tsei2
T6SS
1 2 3 4 5 6 7 8
+ - + + + - + +
- + + + - + + +
+ + + - + + + -
whole cells
supernatant
VgrG1a-Tse2
VgrG1a

Fig 3B
VgrG1a ladder

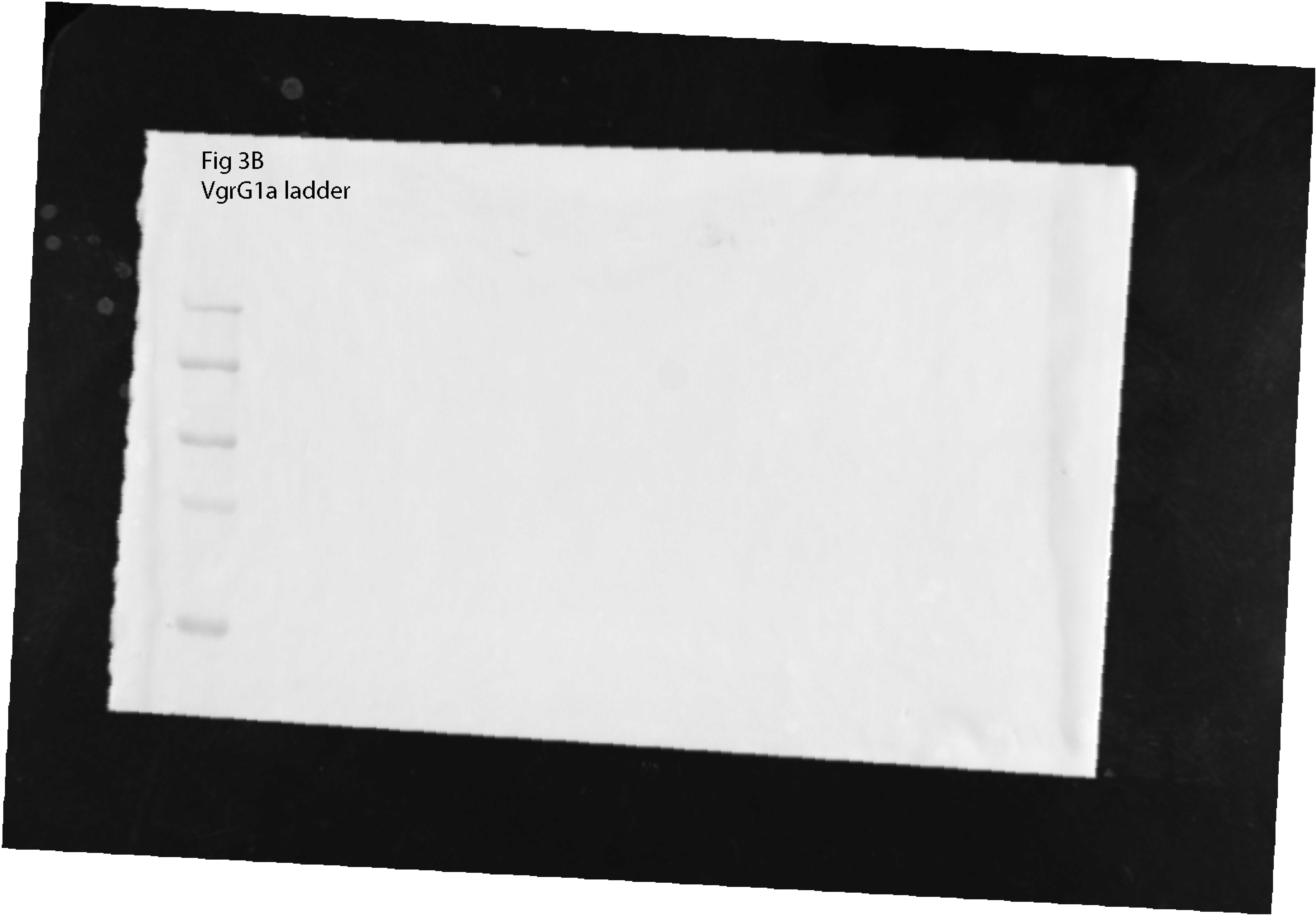

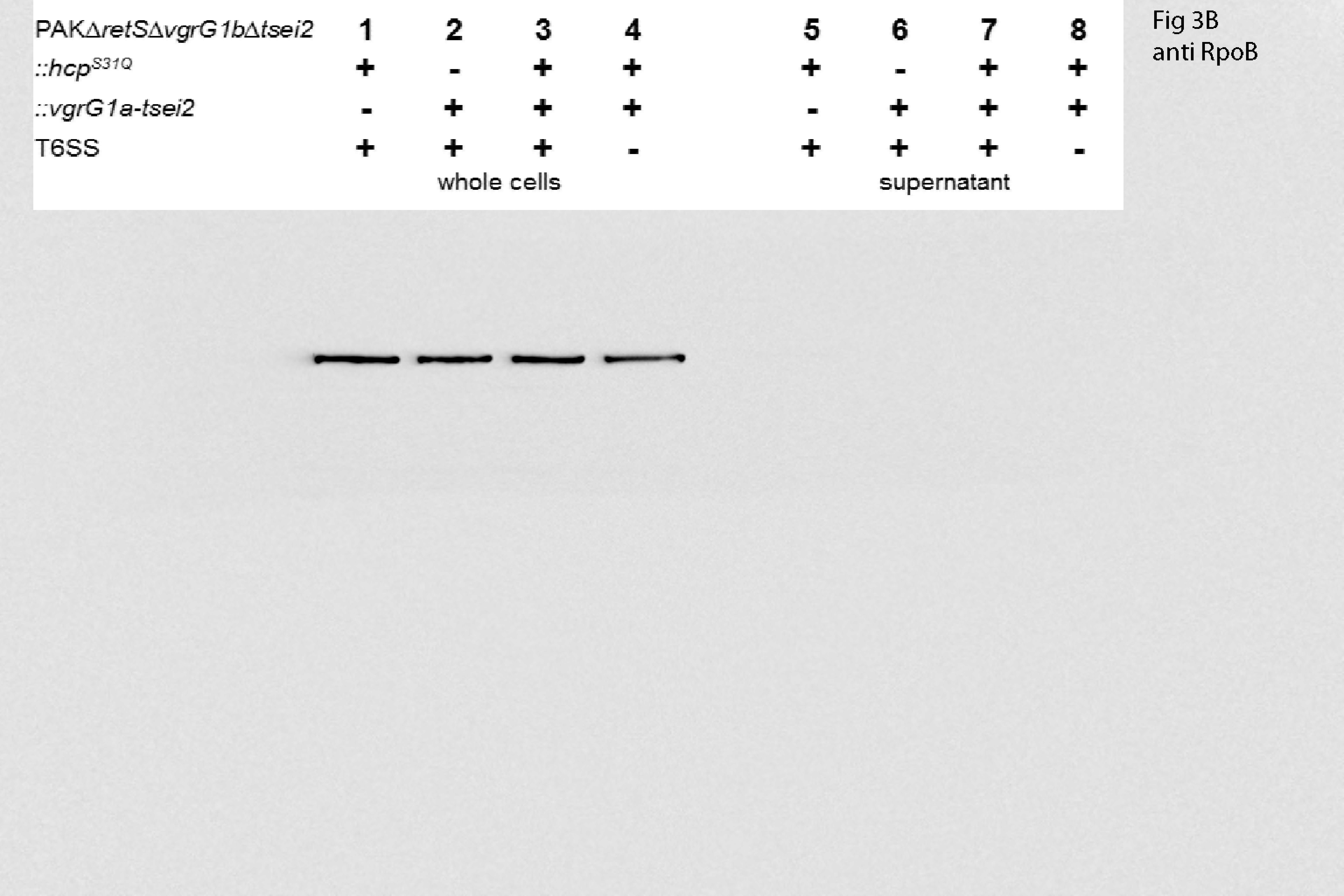

Fig 3B
anti RpoB

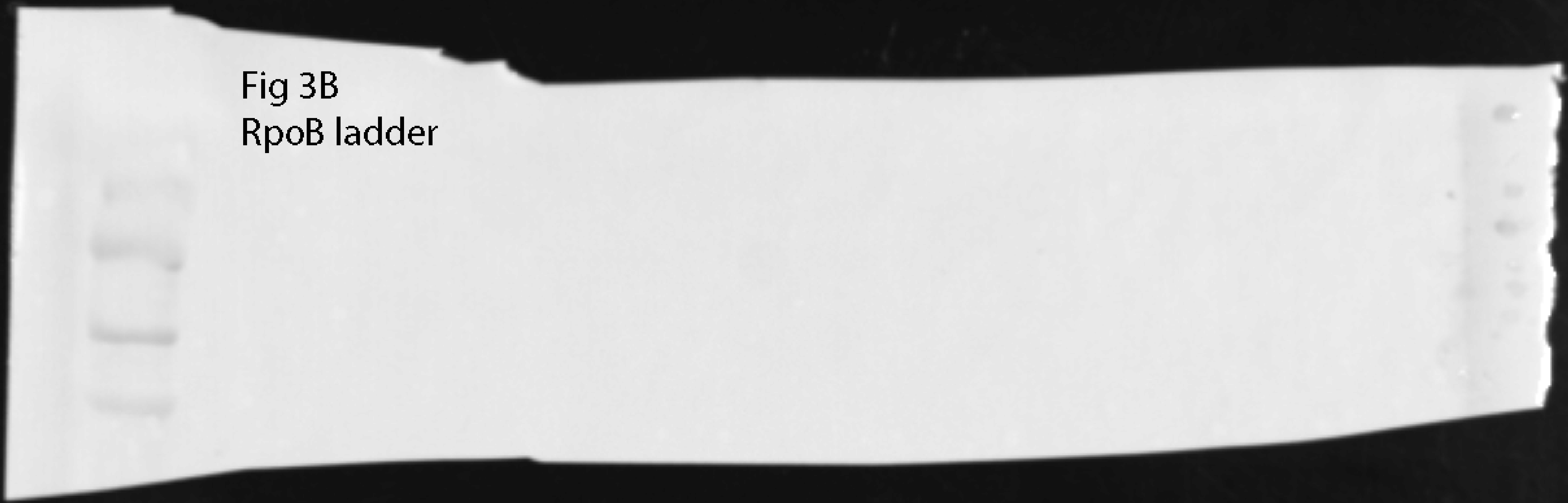

Fig 3B
RpoB ladder

Fig 3B
anti Hcp1

| PAKΔretSΔvgrG1bΔtsei2 | 1 | 2 | 3 | 4 | 5 | 6 | 7 | 8 |
|---|---|---|---|---|---|---|---|---|
| ::hcp$^{S31Q}$ | + | - | + | + | + | - | + | + |
| ::vgrG1a-tsei2 | - | + | + | + | - | + | + | + |
| T6SS | + | + | + | - | + | + | + | - |
| | whole cells | | | | supernatant | | | |

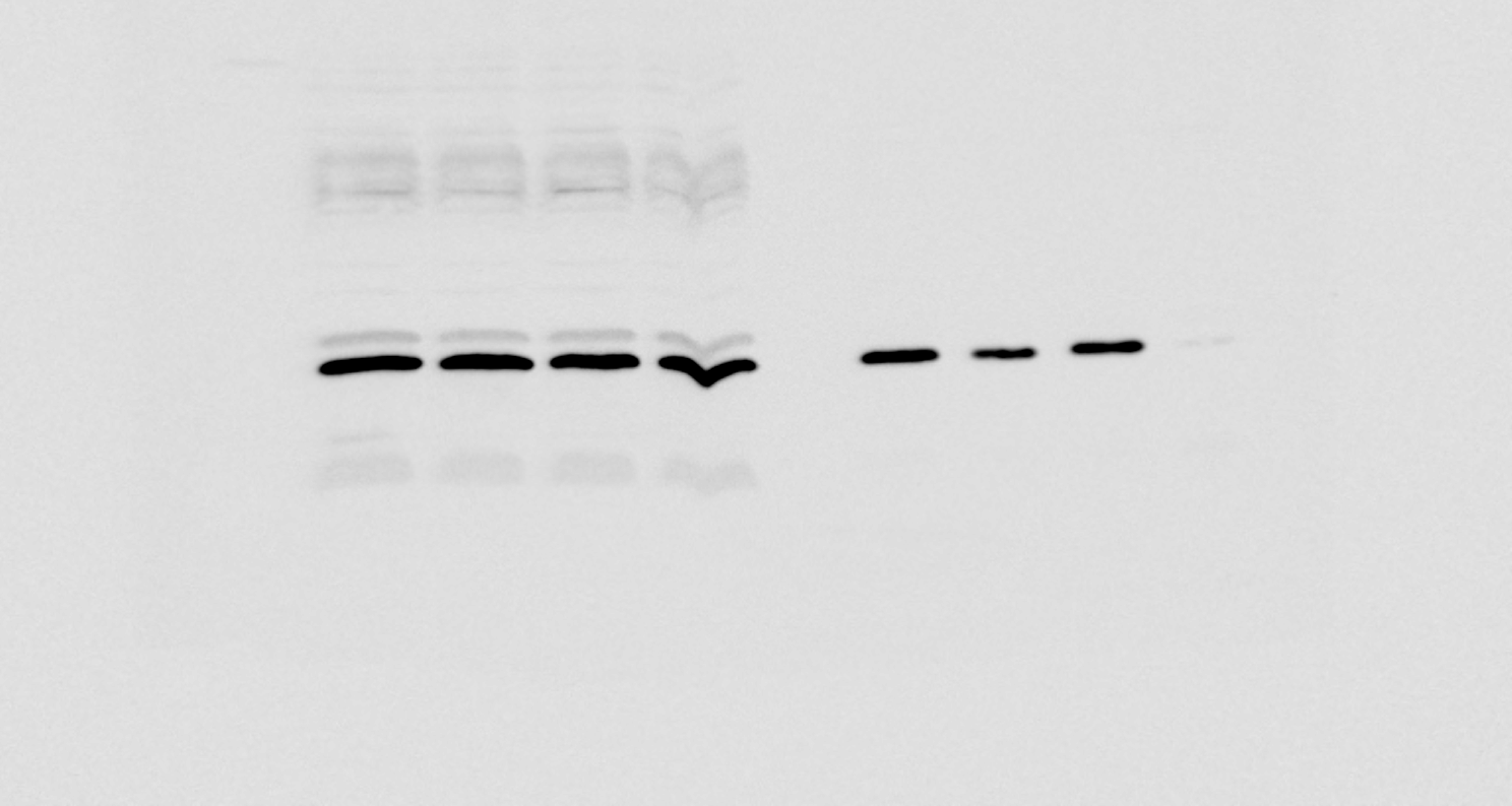

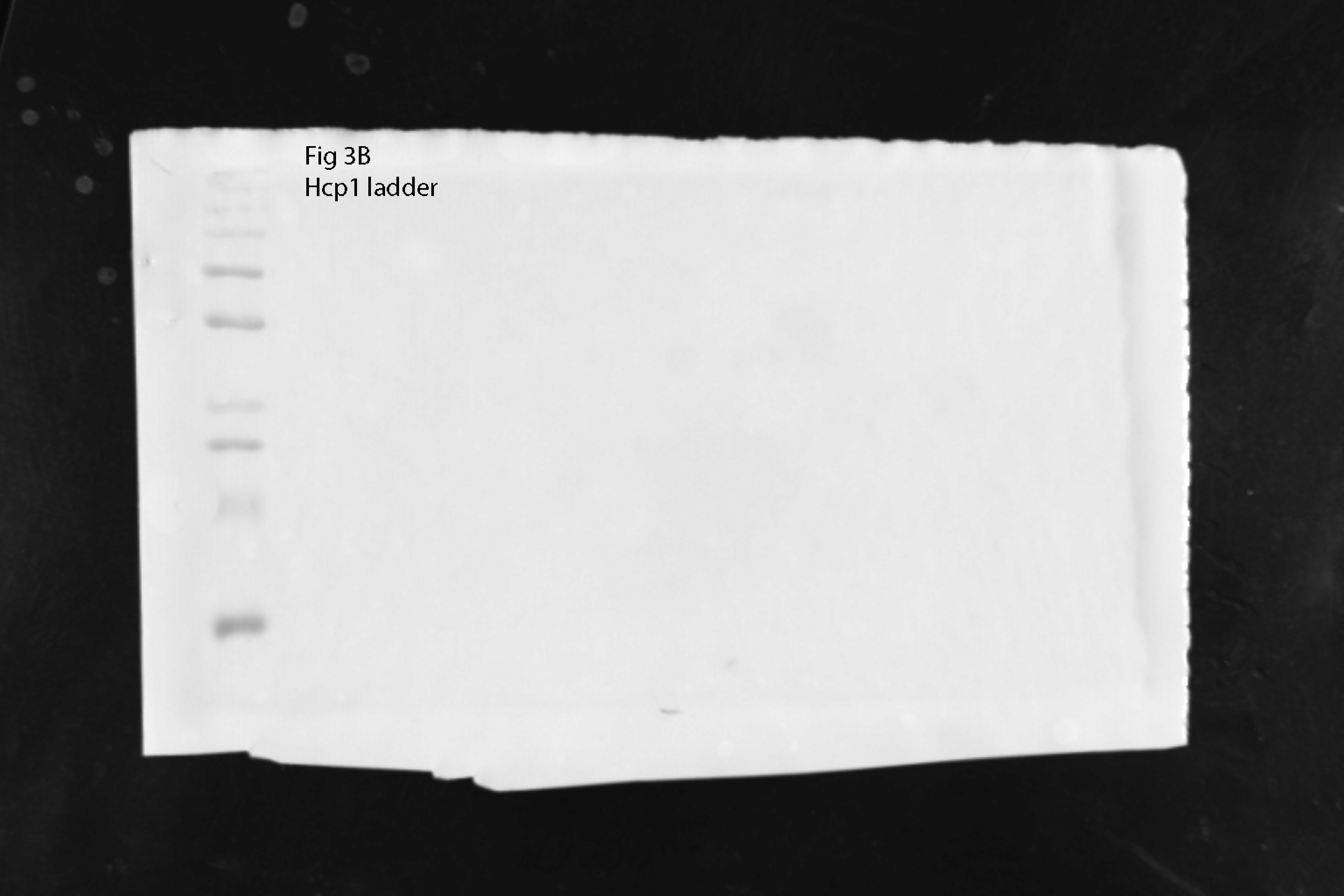
Fig 3B
Hcp1 ladder

Fig 3B
anti Tse3

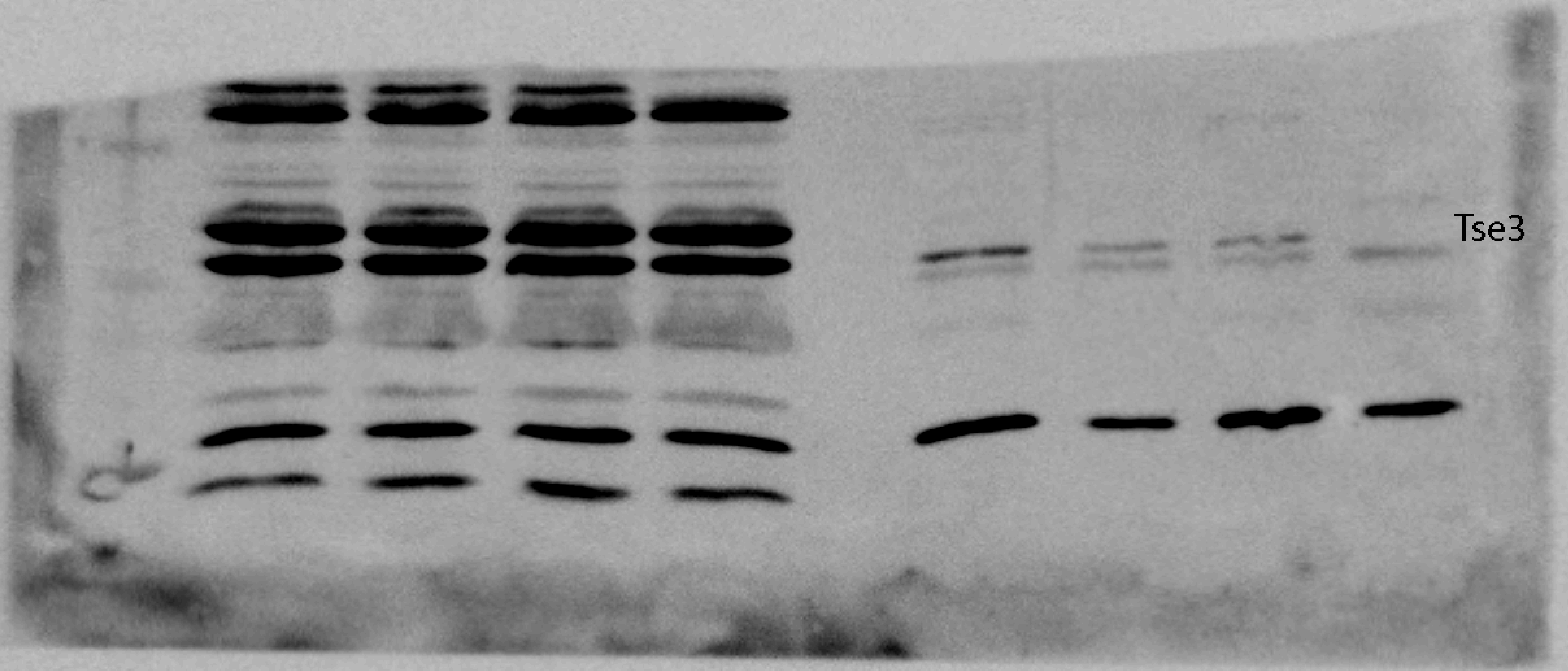


| | 1 | 2 | 3 | 4 | 5 | 6 | 7 | 8 |
|---|---|---|---|---|---|---|---|---|
| PAKΔ*retS*Δ*vgrG1b*Δ*tsei2* | 1 | 2 | 3 | 4 | 5 | 6 | 7 | 8 |
| ::*hcp*$^{S31Q}$ | + | - | + | + | + | - | + | + |
| ::*vgrG1a-tsei2* | - | + | + | + | - | + | + | + |
| T6SS | + | + | + | - | + | + | + | - |
| | | whole cells | | | | supernatant | | |

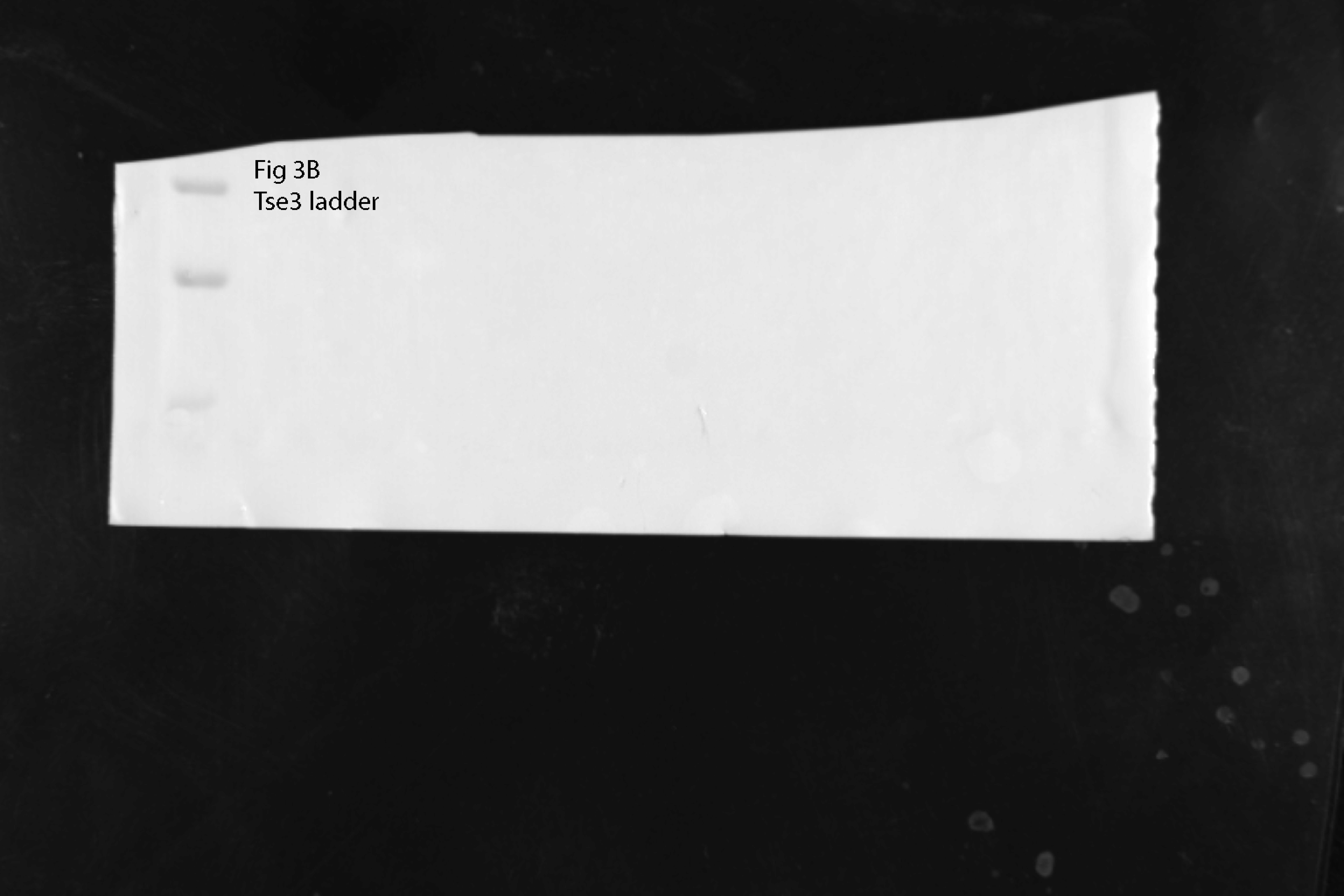
Fig 3B
Tse3 ladder
