# Supplementary material for: Manipulating the type VI secretion system spike to shuttle passenger proteins
Source: PLoS One. 2020 Feb 26;15(2):e0228941. doi: 10.1371/journal.pone.0228941 (PMC7043769; doi:10.1371/journal.pone.0228941)
Supplement: S1 Table — Names of the VgrGs are adapted from the literature with superscripted abbreviations corresponding to the organism the VgrG is derived from (Paer—P. aeruginosa; Vcho–V. cholerae; Ahyd–A. hydrophila; Bpse–B. pseudomallei; Ftul–Francisella tularensis). If no organism is mentioned, its source is P. aeruginosa. Artificially chimeric VgrGs with fused effector domains are highlighted with a grey background. The table lists the enzymatic activities of the extension domains and their size. It further highlights whether the evolved VgrG was found secreted in the supernatant and whether translocation in either eukaryotic or prokaryotic cells was shown. (DOCX) [file pone.0228941.s005.docx]

| evolved VgrG | effector domain | size | secreted? | delivered? | ref. |
| --- | --- | --- | --- | --- | --- |
| VgrG2b^Paer^ | metallo-peptidase | 27 kDa | yes | HeLa cells | [1] |
| VgrG-1^Vcho^ | actin cross-linking | 42 kDa | yes | J774 cells, CHO cells | [2-4] |
| VgrG-1^715Vcho^-Bla | β-lactamase | 30 kDa | yes | J774 cells | [5] |
| VgrG-1^Vcho^-Bla | actin cross-linking β-lactamase | 42 kDa 30 kDa | yes | J774 cells, CHO cells | [5] |
| VgrG-3^Vcho^ | peptidoglycan degrading | 33 kDa | yes | prey cells | [6, 7] |
| VgrG3^708Vcho^-NucSe1 | nuclease | 52 kDa |  | prey cells | [7] |
| VgrG1^Ahyd^ | actin ADP-ribosylating | 23 kDa | yes | HeLa cells | [8] |
| VgrG1^Ahyd^-Bla | actin ADP-ribo-sylating β-lactamase | 23 kDa 30 kDa | yes | HeLa cells | [8] |
| VgrG5^Bpse^ | membrane fusion | 37 kDa | yes |  | [9] |
| VgrG^Ftul^-Bla | β-lactamase | 30 kDa | yes | macrophages | [10] |
| VgrG1a-VgrG2b-CT | metallo-peptidase | 27 kDa | yes |  | this study |
| VgrG1a-Bla_TEM-1_ | β-lactamase | 30 kDa | yes | not HeLa | this study |
| VgrG4b-Bla_TEM-1_ | β-lactamase | 30 kDa | yes | not HeLa | this study |
| VgrG1a-Tse2 | putative ADP-ribosylating | 18 kDa | minor | (prey cells) | this study |
| VgrG4b-PldA | lipase | 122 kDa | yes | prey cells | [11] |

**References**

1. Sana TG, Baumann C, Merdes A, Soscia C, Rattei T, Hachani A, et al. Internalization of Pseudomonas aeruginosa Strain PAO1 into Epithelial Cells Is Promoted by Interaction of a T6SS Effector with the Microtubule Network. MBio. 2015;6(3). doi: 10.1128/mBio.00712-15. PubMed PMID: 26037124.

2. Pukatzki S, Ma AT, Revel AT, Sturtevant D, Mekalanos JJ. Type VI secretion system translocates a phage tail spike-like protein into target cells where it cross-links actin. Proceedings of the National Academy of Sciences of the United States of America. 2007;104(39):15508-13. doi: 10.1073/pnas.0706532104. PubMed PMID: 17873062; PubMed Central PMCID: PMC2000545.

3. Ma AT, Mekalanos JJ. In vivo actin cross-linking induced by Vibrio cholerae type VI secretion system is associated with intestinal inflammation. Proc Natl Acad Sci U S A. 2010;107(9):4365-70. doi: 10.1073/pnas.0915156107. PubMed PMID: 20150509; PubMed Central PMCID: PMC2840160.

4. Durand E, Derrez E, Audoly G, Spinelli S, Ortiz-Lombardia M, Raoult D, et al. Crystal structure of the VgrG1 actin cross-linking domain of the Vibrio cholerae type VI secretion system. J Biol Chem. 2012;287(45):38190-9. Epub 2012/08/18. doi: 10.1074/jbc.M112.390153. PubMed PMID: 22898822; PubMed Central PMCID: PMCPMC3488088.

5. Ma AT, McAuley S, Pukatzki S, Mekalanos JJ. Translocation of a Vibrio cholerae type VI secretion effector requires bacterial endocytosis by host cells. Cell host & microbe. 2009;5(3):234-43. doi: 10.1016/j.chom.2009.02.005. PubMed PMID: 19286133; PubMed Central PMCID: PMC3142922.

6. Brooks TM, Unterweger D, Bachmann V, Kostiuk B, Pukatzki S. Lytic activity of the Vibrio cholerae type VI secretion toxin VgrG-3 is inhibited by the antitoxin TsaB. J Biol Chem. 2013;288(11):7618-25. doi: 10.1074/jbc.M112.436725. PubMed PMID: 23341465; PubMed Central PMCID: PMC3597803.

7. Ho BT, Fu Y, Dong TG, Mekalanos JJ. Vibrio cholerae type 6 secretion system effector trafficking in target bacterial cells. Proceedings of the National Academy of Sciences of the United States of America. 2017;114(35):9427-32. doi: 10.1073/pnas.1711219114. PubMed PMID: 28808000; PubMed Central PMCID: PMCPMC5584461.

8. Suarez G, Sierra JC, Erova TE, Sha J, Horneman AJ, Chopra AK. A type VI secretion system effector protein, VgrG1, from Aeromonas hydrophila that induces host cell toxicity by ADP ribosylation of actin. J Bacteriol. 2010;192(1):155-68. doi: 10.1128/JB.01260-09. PubMed PMID: 19880608; PubMed Central PMCID: PMC2798274.

9. Toesca IJ, French CT, Miller JF. The Type VI secretion system spike protein VgrG5 mediates membrane fusion during intercellular spread by pseudomallei group Burkholderia species. Infection and immunity. 2014;82(4):1436-44. doi: 10.1128/IAI.01367-13. PubMed PMID: 24421040; PubMed Central PMCID: PMC3993413.

10. Bröms JE, Meyer L, Sun K, Lavander M, Sjöstedt A. Unique substrates secreted by the type VI secretion system of *Francisella tularensis* during intramacrophage infection. PLoS One. 2012;7(11):e50473. doi: 10.1371/journal.pone.0050473. PubMed PMID: 23185631; PubMed Central PMCID: PMC3502320.

11. Wettstadt S, Wood TE, Fecht S, Filloux A. Delivery of the Pseudomonas aeruginosa Phospholipase Effectors PldA and PldB in a VgrG- and H2-T6SS-Dependent Manner. *Front Microbiol*. 2019;10:1718. doi: 10.3389/fmicb.2019.01718. PubMed PMID: 31417515; PubMed Central PMCID: PMCPMC6684961.
